# Atypical B cells and inflammatory profiles delineate immunity to influenza vaccination in First Nations and non-Indigenous people with chronic multimorbidity

**DOI:** 10.1038/s41467-026-73988-z

**Published:** 2026-06-05

**Authors:** Morgan J. Skinner, Lukasz Kedzierski, Ruth A. Purcell, Mark Mayo, Bianca F. Middleton, Lilith F. Allen, Ruth R. Hagen, Alexandra Hinchcliff, Matilda Clark, Caitlin Kent, Malet Aban, Heidi Peck, Hayley A. McQuilten, Arnold Reynaldi, Ashleigh I. Holloway, Angelica Tan, Vanessa Rigas, Erin Gargen, Miles P. Davenport, Stephen J. Kent, Ian Barr, Hyon-Xhi Tan, Adam K. Wheatley, Amy W. Chung, Jane Nelson, Adrian Miller, Thi H. O. Nguyen, Jane Davies, Louise C. Rowntree, Katherine Kedzierska

**Affiliations:** 1 Department of Microbiology and Immunology, University of Melbourne, Peter Doherty Institute for Infection and Immunity, Melbourne Victoria, Australia; 2 Menzies School of Health Research, Darwin, Northern Territory Australia; 3 WHO Collaborating Centre for Reference and Research on Influenza, Melbourne, Victoria, Australia; 4 Kirby Institute, University of New South Wales, Sydney, New South Wales Australia; 5 Melbourne Sexual Health Centre, Infectious Diseases Department, Alfred Health, Central Clinical School, Monash University, Melbourne, Victoria, Australia; 6 Indigenous Engagement, CQUniversity, Townsville, Queensland Australia; 7 Center for Influenza Disease and Emergence Response (CIDER), Athens, GA USA

**Keywords:** Influenza virus, Immunological memory

## Abstract

Indigenous people are disproportionately impacted by influenza viruses and chronic multimorbidity. Yet, the impact of comorbidities on immunity towards influenza vaccination is unknown. We recruited Australian First Nations and non-Indigenous people vaccinated with seasonal inactivated influenza vaccines and assessed their humoral and cellular responses in the context of comorbidities at baseline and after immunisation. Our study highlights prevalence of multimorbidity in First Nations people, associated with elevated baseline cellular activation, pro-inflammatory cytokines and agalactosylated IgG. Following vaccination, all groups had increased antibody titres and haemagglutinin-specific IgD^-^ B-cell frequencies as compared to baseline. However, we reveal increased prevalence of pro-inflammatory atypical B cells within influenza haemagglutinin-specific IgD^-^ B cells and lack of significant circulating T follicular helper type-1 cell activation in individuals with comorbidities, correlating with multimorbidity-associated baseline inflammatory features. Our findings thus reveal that vaccinees with comorbidities, both Australian First Nations and non-Indigenous participants, can mount antibody responses following influenza vaccination, although their cellular immune features, haemagglutinin-specific IgD^-^ B cells and cT_FH_1 compartments, display features of perturbed humoral axis functionality linked to multimorbidity-associated inflammation and IgG glycosylation patterns at baseline. Our study supports influenza vaccination for individuals with comorbidities, especially relevant to Indigenous populations with prevalent multimorbidity.

## Abstract (web summary)

Skinner et al. dissects immune responses to seasonal influenza vaccination in a high-risk comorbid population of Australian First Nations and non-Indigenous people, highlighting significant baseline inflammation and atypical memory B cells.

## Introduction

Indigenous people globally experience disproportionate rates of morbidity and mortality from seasonal and pandemic influenza viruses^1–5^. This has been well-documented in Australia, home to 789,300 self-identifying First Nations people^6^. During the 1918–1919 H1N1 influenza pandemic, Australian First Nations communities experienced up to 50% mortality, in contrast to <1% mortality reported for the rest of Australia^5^. In the more recent 2009 H1N1 influenza pandemic, First Nations people accounted for 16% of total hospitalisations, while representing only ~2.5% of Australia’s total population^4^. A recent systematic review of influenza hospitalisations and mortality, found Australian First Nations people are three times more likely to be hospitalised from seasonal influenza compared to non-Indigenous Australians^2^. Similar observations have been made among Indigenous populations in New Zealand, Canada, Brazil and the United States^2,3,7,8^. While epidemiological data clearly demonstrate the health gap that exists for Indigenous people, immunological studies investigating perturbations in anti-viral immunity in this vulnerable population are generally lacking.

Vaccination is the key strategy for prevention of severe influenza disease. In many countries, including Australia, USA and Canada, Indigenous populations are prioritised for seasonal and pandemic influenza vaccinations^1,9–13^. Despite this, Indigenous people globally face higher viral-respiratory rates and influenza-related hospitalisations^1,2,14,15^. Immunity towards seasonal inactivated influenza vaccination (IIV) in Indigenous populations remains understudied. Our recent studies showed healthy Australian First Nations people produce prototypical responses to IIV and SARS-CoV-2 mRNA vaccination, comparable to non-Indigenous participants^16,17^. Studies of Indigenous Canadians also report robust haemagglutinin (HA) inhibition (HAI) antibody responses towards pandemic H1N1 influenza vaccination^18^. However, our investigations of SARS-CoV-2 mRNA vaccine responses revealed First Nations and non-Indigenous people living with chronic diseases (renal disease, diabetes, inflammatory bowel syndrome) had reduced SARS-CoV-2 antibody titres, spike-specific B cells and follicular helper T (T_FH_) cells, linked to high baseline inflammation^17^. As Indigenous populations globally have profound overrepresentations of chronic diseases, including renal disease, cardiac disease, diabetes mellitus and liver disease^19^, it is of key importance to understand the impact of comorbidities on immunity toward seasonal influenza vaccination.

We recruited 194 First Nations and 150 non-Indigenous participants vaccinated with a quadrivalent IIV during the 2022, 2023 and/or 2024 Southern Hemisphere influenza seasons. Exhibiting a range of comorbidity presentations, this cohort presented a rare opportunity to define features of perturbed baseline immunity and evaluate vaccine responsiveness in co- and multimorbidity affected individuals of both Indigenous and non-Indigenous ethnicity. We measured concentrations of plasma cytokines and chemokines, assessed bulk IgG glycosylation patterns and frequencies of activated immune cells in pre-vaccination peripheral blood samples. Our baseline analyses revealed a multimorbidity-associated inflammatory signature comprised of increased pro-inflammatory cytokines including MCP-1, IL-6 and IL-18, agalactosylated IgG antibodies and activated NK cells, ${{{\rm{\gamma }}}}{{{\rm{\delta }}}}$, CD4^+^ and CD8^+^ T cells. Post-vaccination, First Nations and non-Indigenous vaccinees produced HAI antibody responses against all vaccine components and had increased HA-specific IgD^-^CD10^-^ B cells populations, irrespective of comorbidities. Phenotypic analysis, however, revealed higher inflammation-associated CD27^- ^CD21^-^ atypical lineage B cell phenotypes and lack of significant circulating (c)T_FH_1 activation in individuals with comorbidities, correlating with multimorbidity-associated inflammatory features. Our study thus demonstrates that First Nations and non-Indigenous participants with multiple chronic diseases can mount antibody responses towards influenza vaccination, however their HA-specific B cell and cT_FH_1 compartments display distinct perturbed features post-IIV, linked to multimorbidity-associated inflammatory signatures and IgG glycosylation patterns at baseline.

## Results

### High prevalence of morbidity in Australian First Nations participants

**Fig. 1:**
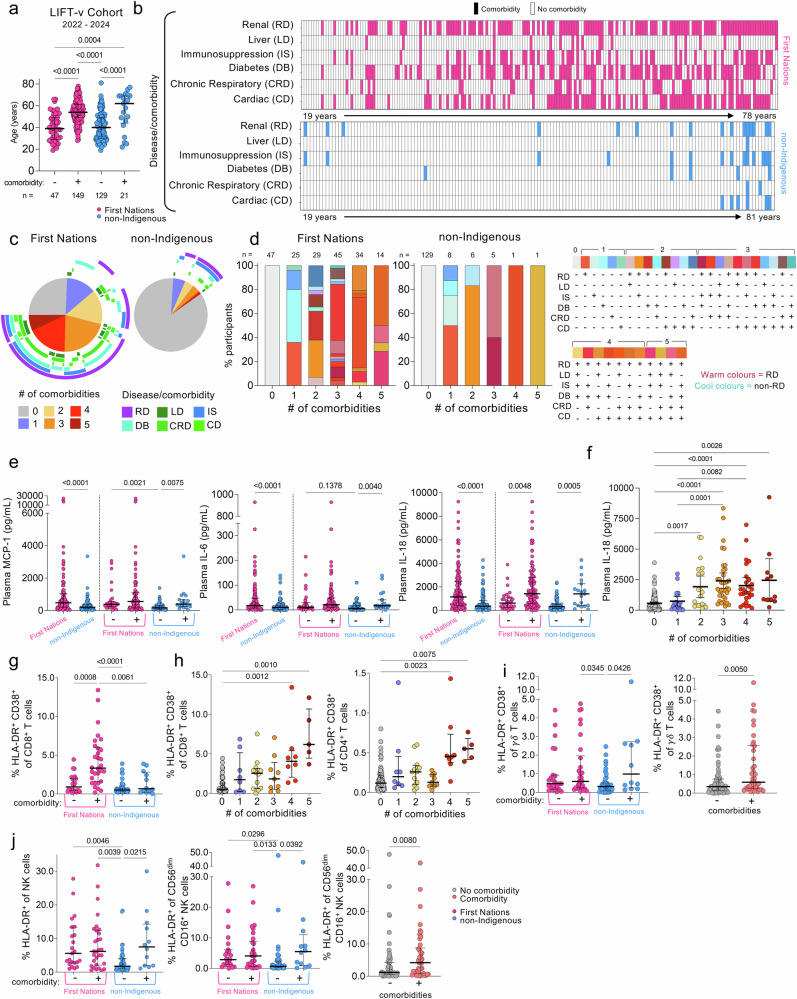
Multimorbidity is over-represented in Australian First Nations participants and is associated with increased baseline inflammatory plasma cytokines and immune hyperactivation. **a** Participant numbers and age range for First Nations and non-Indigenous cohorts across three-year sample collection period. **b** Categorical heat map ordered according to age, where each column represents a single participant, depicting presence of disease/comorbidity conditions among First Nations (pink) and non-Indigenous (blue) cohorts. **c** Comparison of frequency of multimorbidity effected individuals between First Nations (left) and non-Indigenous (right) cohorts. Pie chart segments represent proportions of participants in each multimorbidity group. Coloured outer rings represent presence of specific comorbidities within groups. **d** Specific comorbidity presentation profiles of First Nations (left) and non-Indigenous (right) individuals according to number of comorbidities. Presentations which include renal disease (RD) have been shown in warm toned colours, while non-renal disease groups are shown in cool toned colours. **e** Plasma concentration of MCP-1 (left), IL-6 (middle) and IL-18 (right) between First Nations (pink, *n* = 125) and non-Indigenous (blue, *n* = 99) cohorts, with (+) (n_FN_=95, n_NI_ = 19) and without (-) (n_FN_=30, n_NI_ = 80) comorbidities. **f** Level of plasma IL-18 by number of comorbidities (n_0_ = 110, n_1_ = 20, n_2_ = 20, n_3_ = 36, n_4_ = 26, n_5_ = 11) **g** Frequency of HLA-DR^+^CD38^+^ activated CD8^+^ T cells among First Nations (*n* = 54) and non-Indigenous (*n* = 55) people with (n_FN=_30, n_NI=_12) and without (n_FN_=24, n_NI_ = 43) comorbidities. **h** Frequency of HLA-DR^+^CD38^+^ CD8^+^ (left) and CD4^+^ (right) by number of comorbidities (n_0_ = 67, n_1_ = 8, n_2_ = 12, n_3_ = 9, n_4_ = 8, n_5_ = 5). **i** Activated HLA-DR^+^CD38^+^ ${{{\rm{\gamma }}}}{{{\rm{\delta }}}}$ T cell frequency grouped by ethnicity (n_FN_=54, n_NI_ = 55) and comorbidities (left) and comorbidity status only (right) (n_NCM_=67, n_CM_ = 42). **j** Frequency of HLA^-^DR^+^ total NK cells (left) and CD56^dim^CD16^+^ NK cells (middle) between First Nations (*n* = 54) and non-Indigenous (*n* = 55) with (n_FN=_30, n_NI=_12) and without (n_FN_=24, n_NI_ = 43) comorbidities, and CD56^dim^CD16^+^ NK cells grouped by only comorbidity status (right) (n_NCM_=67, n_CM_ = 42). **e**–**j** Bolded line represents median and error bars depict interquartile range (IQR). Statistical significance (**a**, **e**–**j**) was determined by two-sided Mann-Whitney t test corrected with Dunn’s test for multiple comparisons. Legend abbreviations refer to renal disease (RD), diabetes (DB), chronic respiratory disease (CRD), immunosuppression (IS), cardiac disease (CD) and liver disease (LD).

During the 2022-2024 Southern Hemisphere influenza seasons, 194 First Nations and 150 non-Indigenous adults were recruited via the Menzies School of Health Research in Darwin, and the University of Melbourne, both in Australia (Fig. 1a). Our cohorts were sex-matched between Australian First Nations and non-Indigenous participants, with 57% and 54% female participants, respectively (Supplementary Table 1). Comorbidity-affected individuals were older than those without comorbidities for both First Nations (39 years without comorbidities, 54 years with comorbidities, p < 0.0001) and non-Indigenous cohorts (40 years without comorbidities and 62 years with comorbidities, p < 0.0001) (Fig. 1a). Notably, the median age of First Nations participants with comorbidities trended lower compared to non-Indigenous participants also with comorbidities, although this was not significant (54 and 62 years, respectively) (Fig. 1a). This is consistent with epidemiological data which demonstrate Indigenous peoples in Australia and globally are disproportionately impacted by co- and multimorbidity at younger ages and have shorter predicted lifespans compared to non-Indigenous individuals^19–21^ In our cohorts, one or more chronic diseases were noted in 75% of First Nations and 14% of non-Indigenous participants (Fig. 1b, c). The prevalence of multimorbidity was also more pronounced in the First Nations cohort, with 62.9% (122 of 194) of First Nations individuals having 2 or more comorbidities, compared to 8.6% (13 of 150) in the non-Indigenous cohort (Fig. 1c, d). Strikingly, 24.7% of First Nations participants had 4 or 5 co-morbidities. Key co-morbidities included renal disease (39.2%), diabetes (29.1%), cardiac disease (27.6%) and immunosuppression (21.8%) (Fig. 1b–d; and Supplementary Table 1).

### Increased baseline pro-inflammatory cytokines in participants with comorbidities

Since we previously linked high baseline inflammation in First Nations people with comorbidities with reduced antibody responses following COVID-19 vaccination^17^, we first measured plasma inflammatory mediators prior to influenza vaccination in our cohort (Fig. 1e, f, and Supplementary Fig. 1). MCP-1, IL-6 and IL-18 were higher in First Nations people (Fig. 1e), while IL-1${{{\rm{\beta }}}}$ and TNF were higher in non-Indigenous participants (Supplementary Fig. 1a, b). IL-18 was higher in First Nations and non-Indigenous people with comorbidities (Fig. 1e). Plasma concentrations of IL-18 were higher in participants with 2 or more comorbidities compared to those with 1 or no preexisting conditions (Fig. 1f). Non-Indigenous participants with comorbidities also had higher concentrations of IL-6 compared to those without comorbidities (Fig. 1e). First Nations people without comorbidities displayed higher concentrations of MCP-1 compared to non-Indigenous participants without comorbidities (Fig. 1e). As pre-existing comorbidities substantially impact plasma levels of inflammatory mediators, we assessed vaccine responses in the context of comorbidities and inflammation.

### Baseline immune hyperactivation in First Nations and non-Indigenous people with comorbidities

As pro-inflammatory cytokine milieu can reflect bystander-mediated hyperactivation of immune cells in viral infections and non-communicable chronic diseases^22–25^, we next assessed baseline activation of CD4^+^ and CD8^+^T cells by measuring activation markers: CD38, HLA-DR, ICOS and PD-1 (Fig. 1g, h, and Supplementary Fig. 2a). We detected a higher proportion of double-positive CD38^+^HLA-DR^+^ CD8^+^ T cells, previously associated with influenza disease severity^22,24,26,27^, in First Nations with comorbidities compared to First Nations without comorbidities and non-Indigenous participants regardless of comorbidity status (Fig. 1g). Higher frequencies of activated CD38^+^HLA-DR^+^ CD4^+^ and CD8^+^ T cells were found in participants with increasing number of comorbidities, with the highest frequencies of these activated T cells detected in participants with 4 or 5 comorbidities compared to participants with no comorbidities (Fig. 1h). Our analyses of innate cells (Fig. 1i, j, and Supplementary Fig. 2b) showed higher proportion of CD38^+^HLA-DR^+^ ${{{\rm{\gamma }}}}{{{\rm{\delta }}}}$ T cells in people with comorbidities, regardless of ethnicity (Fig. 1i). Additionally, in First Nations people, HLA-DR^+^ cytotoxic CD56^dim^CD16^+^ NK cells were higher in participants with comorbidities (Fig. 1j). Thus, our data provide evidence for the baseline immune cellular hyperactivation in our Australian First Nations cohort, primarily associated with comorbidities.

### Pro-inflammatory IgG glycoforms in First Nations and non-Indigenous participants with comorbidities

**Fig. 2:**
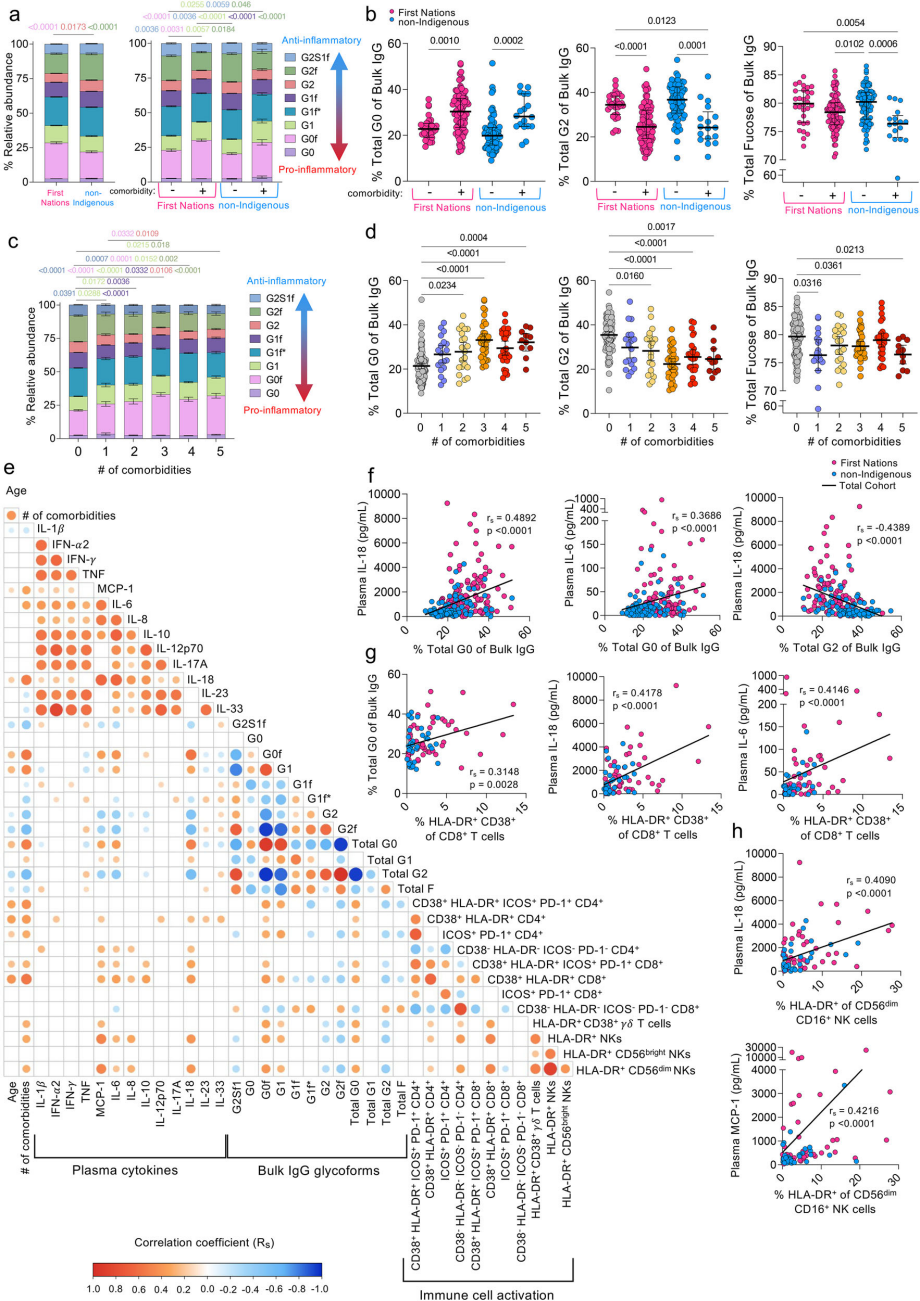
Pro-inflammatory IgG glycoforms increase with multimorbidity and contribute to multifaceted comorbidity-associated inflammatory signatures. **a** Comparison of baseline IgG glycan profiles between First Nations (*n* = 125) and non-Indigenous (*n* = 97) people (left) with (+) (n_FN_=95, n_NI_ = 18) and without (-) (n_FN_=30, n_NI_ = 79) comorbidities (right). **b** Total G0 (left, G0 + G0f), G2 (middle, G2 + G2f + G2S1f) and fucosylated IgG (right, G0f + G1f + G1f* + G2f + G2S1f) antibodies in First Nations and non-Indigenous people (n_FN_=95, n_NI_ = 18) and without (-) (n_FN_=30, n_NI_ = 79) comorbidities. **c** Stacked plot displaying relative abundance of bulk IgG glycoforms of total cohort participants by number of comorbidities (n_0_ = 109, n_1_ = 19, n_2_ = 21 n_3_ = 36, n_4_ = 26, n_5_ = 11). **d** Total G0, G2 and fucosylated IgG antibodies in total cohort by number of comorbidities (n_0_ = 109, n_1_ = 19, n_2_ = 21 n_3_ = 36, n_4_ = 26, n_5_ = 11). **e** Correlation matrix of spearman correlations of baseline inflammatory features in total study cohort including plasma cytokines, bulk IgG glycans and hyperactivated immune cells. Only significant correlations shown. **f** Representative plots of significant Spearman correlations between pro-inflammatory plasma cytokines (IL-18 or IL-6) and bulk IgG glycans (Total G0 or G2) (*n* = 222). **g** Representative plots of significant Spearman correlations between Total G0 or plasma cytokines (IL-18 or IL-6) and HLA-DR^+^CD38^+^ CD8^+^ T cells (*n* = 88). **h** Representative plots of significant Spearman correlations between plasma cytokines (IL-18 or MCP-1) and HLA-DR^+^ CD56^dim^CD16^+^ NK cells (*n* = 86). Symbols (**f–h**) indicate First Nations (pink) and non-Indigenous (blue) participants. **a**, **c** Mean and standard error of mean (SEM) are shown. On scatter dot plots (**b**, **d**) the bolded line represents the median and error bars depict IQR. Statistical significance of IgG glycoform abundances between First Nations, non-Indigenous, comorbidity and non-comorbidity groups (**a**, **c**) was calculated by two-way ANOVA with two-sided Tukey’s test for multiple comparisons. Statistical significance (**b**, **d**) was determined by two-sided Mann-Whitney t test corrected with Dunn’s test for multiple comparisons. Correlations (**e**–**h**) calculated using two-sided Spearman correlation.

Differential glycosylation patterns of the Fc portion of IgG antibodies can act as a biomarker of inflammation, disease progression and severity, and more recently, the potential for effective vaccine-induced respiratory virus humoral immunity^17,28–38^. Post translational glycosylation (enzymatic addition of polysaccharide chains) of IgG is a key regulator of antibody Fc function^39^, including enhanced clearance of pathogens and virally-infected cells^40^. Differential glycosylation patterns can also modify Fc receptor engagement^39,41^. They also influence inflammation in the lungs regardless of viral replication^42^. Our IgG glycosylation baseline analysis revealed higher abundance of pro-inflammatory G0f (core fucose/no galactose) and lower frequency of anti-inflammatory G2 (two galactose units) and G2S1f (two galactose units/one sialic acid units/core fucose) IgG in First Nations participants compared to non-Indigenous individuals (Fig. 2a, Supplementary Fig. 3a). We also found higher abundance of pro-inflammatory glycoform G0f in individuals with comorbidities, while anti-inflammatory G2S1f and G2f were higher in participants without comorbidities (Fig. 2a, and Supplementary Fig. 3a). Total abundance of IgG G0 glycoforms (G0 and G0f) was higher in First Nations and non-Indigenous participants with comorbidities (Fig. 2b, and Supplementary Fig. 3a) as compared to those without. We also observed greater abundance of total G2 (G2, G2f and G2S1f) and fucosylated (G0f, G1f, G1f*, G2f and G2S1f) IgG glycoforms in participants without comorbidities compared to those with comorbidities, regardless of ethnicity (Fig. 2b, and Supplementary Fig. 3a). IgG afucosylation is a pro-inflammatory biomarker, therefore abundance of fucosylated IgG can be utilised as a reciprocal anti-inflammatory marker^39^. Participants with two or more pre-existing conditions had bulk IgG glycosylation profiles with greater representation of pro-inflammatory glycans as compared to those with no comorbidities (Fig. 2c, and Supplementary Fig. 3b,c). Total G0 abundance was higher in participants with 2 or more comorbidities and consequently, total G2 and fucosylated glycoforms decreased with increasing multimorbidity (Fig. 2d, and Supplementary Fig. 3b). Thus, comorbidities are associated with markedly altered glycosylation patterns. This is especially evident in First Nations people, who have increased prevalence of chronic comorbidities.

### IgG glycoforms, plasma cytokines and immune cell activation contribute to comorbidity-associated inflammation

To define key correlations across our independently measured soluble inflammatory mediators, cellular hyperactivation and antibody IgG glycosylation patterns, we generated a correlation matrix (Fig. 2e). We observed correlative relationships between pro-inflammatory cytokines, bulk IgG glycosylation patterns, and immune cell activation together with number of comorbidities (Fig. 2e). Total G0 IgG abundance was positively correlated with plasma concentrations of both IL-18 (r_s_=0.4892, *p* < 0.0001) and IL-6 (r_s_=0.3686, *p* < 0.0001) (Fig. 2f). Reciprocally, IL-18 negatively correlated with total abundance of anti-inflammatory G2 glycoforms (r_s_ = -0.4389, *p* < 0.0001) (Fig. 2f). We observed similar correlative relationships with immune cell activation, with the frequency of activated HLA-DR^+^CD38^+^ CD8^+^ T cells positively correlating with G0 abundance (r_s_=0.3148, *p* = 0.0028), IL-18 (r_s_=0.4178, *p* < 0.0001) and IL-6 (r_s_=0.4146, *p* < 0.0001) (Fig. 2g). Frequency of HLA-DR^+^CD56^dim^CD16^+^ NK cells also positively correlated with plasma IL-18 (r_s_=0.4090, *p* < 0.0001) and MCP-1 (also known as CCL2; r_s_ = 0.4216, *p* < 0.0001) levels (Fig. 2h). MCP-1 is a mediator of inflammatory conditions including kidney disease, and a potent activator, triggering NK differentiation, enhancing cytotoxicity and promoting cell migration to sites of inflammation or infection^43–45^. Independently, IL-18, IL-6, MCP-1, G0 abundance, HLA-DR^+^CD38^+^ CD8^+^ T cells and HLA-DR^+^CD56^dim^CD16^+^ NK cells all had positive correlations with number of comorbidities (Fig. 2e). Overall, our data present a distinct multimorbidity-driven baseline inflammatory profile, encompassing inflammatory soluble mediators, differential IgG glycosylation patterns and increased immune cell activation.

### Similar HAI responses following vaccination in First Nations and non-Indigenous vaccinees despite comorbidities

**Fig. 3:**
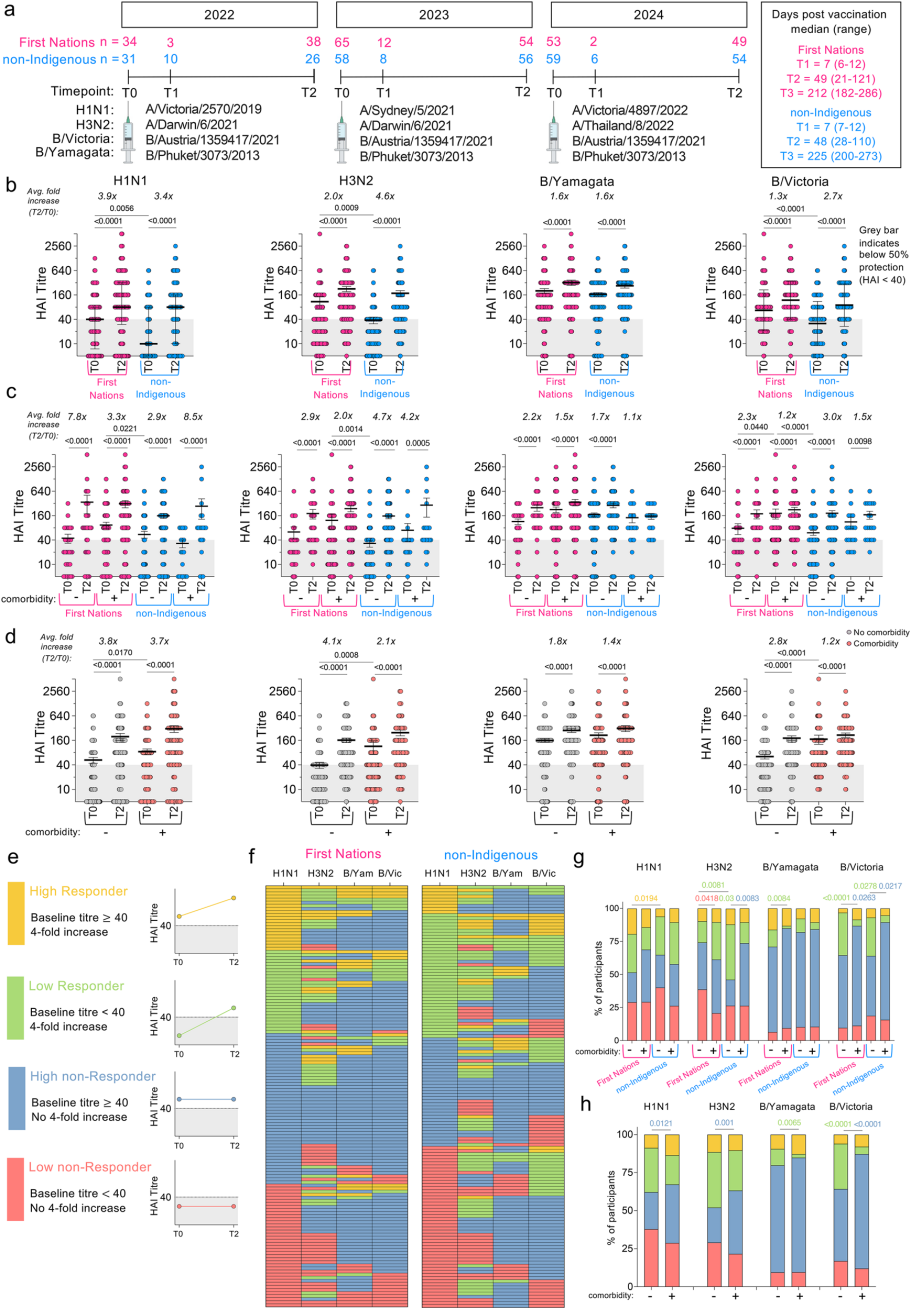
Australian First Nations and non-Indigenous vaccinees with multiple comorbidities produce robust HAI titres against all quadrivalent vaccine components. **a** Vaccine study design and number of participants across three-year collection period for First Nations and non-Indigenous groups with corresponding seasonal quadrivalent influenza vaccination formulations Created in BioRender. Skinner, M. (https://BioRender.com/9a98avm). **b–d** Haemagglutinin inhibiting serum antibodies determined by haemagglutinin inhibition (HAI) assay prior to vaccination (T0) and post-vaccination (T2) against H1, H3, B/Yamagata and B/Victoria vaccine components. Comparison of pre- and post-vaccination strain-specific HAI titres between **b** First Nations (*n* = 137) and non-Indigenous (*n* = 136) participants, **c** with (+) (n_FN_=106, n_NI_ = 19) and without (-) (n_FN_=31, n_NI_ = 117) comorbidities and **d** comorbidity status irrespective of ethnicity (n_NCM_=148, n_CM_ = 125). Bold line indicates mean, and error bars indicate SEM. Data points above grey bar indicates titre of $\ge$40 which confers 50% protection. **e** Definitions of responder status by baseline HAI titre and post-vaccination antibody titre fold change. **f** Categorical heat maps of vaccine component responder status, each row represents an individual vaccinee, ordered according to H1N1 responder status, for First Nations (left) and non-Indigenous (right) cohorts. **g**,** h** Frequency of vaccine strain-specific responder status groups (**g**) First Nations and non-Indigenous with comorbidities and no comorbidities and (**h**) comorbidity status irrespective of ethnicity. Statistical significance was determined (**b–d**) by two-sided Wilcoxon ranked sum test for paired pre- and post-vaccination titres within groups, and two-sided Mann-Whitney test for unpaired comparison between groups corrected with Dunn’s test for multiple comparisons. Statistical significance of responder status group frequency (**g**,** h**) was determined by two-sided Chi square test and/or Fisher’s exact test where any cell value < 5.

To evaluate influenza vaccine-specific responses in Australian First Nations people in context of comorbidities, we sampled participants receiving a quadrivalent inactivated influenza vaccine (IIV) during 2022, 2023 and/or 2024 Southern Hemisphere influenza seasons (Fig. 3a). Blood was taken at baseline (T0; pre-vaccination) as well as post-vaccination at T1 (days 6-12), T2 (days 21-121) and T3 (days 182-286) to assess vaccine-induced immunity (Fig. 3a). Haemagglutinin inhibition (HAI) titres, the best-defined correlate of protection against influenza, with HAI titre of 40 correlating with ~50% protection^46^, were assessed first at baseline and T2. HAI titres against H1N1, H3N2, B/Yamagata and B/Victoria vaccine components increased across all study years for Australian First Nations and non-Indigenous vaccinees post-IIV, with median post-vaccination titres above the 50% protective titre cut-off (Fig. 3b, and Supplementary Fig. 4a). We detected higher baseline H1N1-, H3N2- and B/Victoria-specific HAI titres in First Nations participants compared to non-Indigenous individuals (Fig. 3b), however the geometric mean titres only differed by ~2-fold, which by the sensitivity of the assay, may not indicate a physiologically relevant distinction. These higher baseline titres could be due to repeated seasonal vaccinations, which are strongly recommended for both Australian First Nations people and those with comorbidities^9–11^, and/or recent infections with related strains, however concordant epidemiological and/or immunological data is not available to confirm either hypothesis. When we examined responses per year, we found that neither specific vaccine formulation nor years’ sub-cohort drove our observations (Supplementary Fig. 4a). Our First Nations and non-Indigenous cohorts exhibited a matched proportion of individuals who were vaccinated in the influenza season directly prior to their participation in our study, 70% and 72%, respectively. (Supplementary Fig. 5a, b). Baseline titres against IAV vaccine components were higher in those who received an IIV in the previous year (Supplementary Fig. 5c), however vaccination history did not consistently drive higher HAI titres between First Nations and non-Indigenous participants or across different vaccine components (Supplementary Fig. 5d).

First Nations and non-Indigenous participants with and without comorbidities generated similar HAI responses towards all vaccine components (Fig. 3c, d). Baseline titres of H3N2 and B/Victoria were higher in First Nations participants with comorbidities compared to non-Indigenous people without comorbidities. First Nations participants with comorbidities also displayed higher B/Victoria-specific baseline titres than those without (Fig. 3c). Overall, baseline titres of H1N1, H3N2 and B/Victoria were higher in participants with comorbidities compared to participants without comorbidities, regardless of ethnicity, however T2 post-IIV titres were comparable across groups for all vaccine components (Fig. 3d). In context of multimorbidity, we found robust increases of HAI titres toward all vaccine components across participants with increasing numbers of pre-existing conditions and, where a significant increase was not observed, median post-vaccination titres conferred ~50% seroprotection (Supplementary Fig. 4b).

### Vaccinees with comorbidities have higher proportions of High Non-Responder individuals towards H1N1, H3N2 and B/Victoria vaccine components

We next stratified participants according to four responder status groups based on seroconversion and pre-existing immunity (Fig. 3e). Historically, responders to IIV have been defined as exhibiting a 4-fold increase in HAI titres post-IIV^46^. This definition, however, does not sufficiently characterise the physiological relevance of antibody titres at baseline. We thus defined Low Non-Responders as individuals with baseline HAI titres <40 who did not seroconvert (<4-fold increase). High Non-Responders had baseline HAI titres ≥40 but without a 4-fold increase in HAI titres post-IIV. Low Responders had baseline titres <40 and seroconverted, while High Responders had seroprotective baseline HAI titres ≥40 and seroconverted following IIV (Fig. 3e). Using these definitions, we assessed responder status across individuals and vaccine components (Fig. 3f). In participants with no comorbidities, we found higher proportion of H1N1 High Responders in First Nations people (19.4%) compared to non-Indigenous participants (5.9%), representing both elevated pre-existing and robust humoral immunity in First Nations participants toward H1N1 strains (Fig. 3g). Responder groups were more variable towards H3N2 viruses. Non-Indigenous participants without comorbidities had the highest proportion (41.8%) of H3N2 Low Responders, compared to non-Indigenous with comorbidities (15.8%) and First Nations people without comorbidities (16.1%). Subsequently, non-Indigenous participants with comorbidities had a higher proportion of H3N2 High Non-Responders compared to non-Indigenous without comorbidities, while First Nations people without comorbidities had a higher proportion of H3N2 Low Non-Responders compared to First Nations people with comorbidities (Fig. 3g). First Nations with comorbidities had lower proportion of B/Yamagata and B/Victoria Low Responders (1.88% and 4.7%, respectively) compared to no comorbidity First Nations participants (12.9% and 32.25%, respectively) (Fig. 3g). Participants with comorbidities had a higher proportion of H1N1, H3N2 and B/Victoria High Non-Responders, while B/Yamagata and B/Victoria had less Low Responders, compared to participants without comorbidities (Fig. 3h). Individuals with multimorbidity had a high degree of variation in their responder status (Supplementary Fig.4c). We observed increased proportions of H1N1, H3N2 and B/Victoria Low Non-Responders in groups with no and 1 pre-existing conditions compared to those with 2 or more comorbidities (Supplementary Fig. 4c). Interestingly, the proportion of High Non-Responders increased with the number of comorbidities against all vaccine components (Supplementary Fig. 4c). Collectively, our data show high levels of pre-existing influenza-specific humoral immunity in First Nations participants with comorbidities and demonstrate that participants with multiple chronic diseases mount protective antibody responses towards seasonal IIV.

### Vaccinees with comorbidities have durable longitudinal HAI titres

**Fig. 4:**
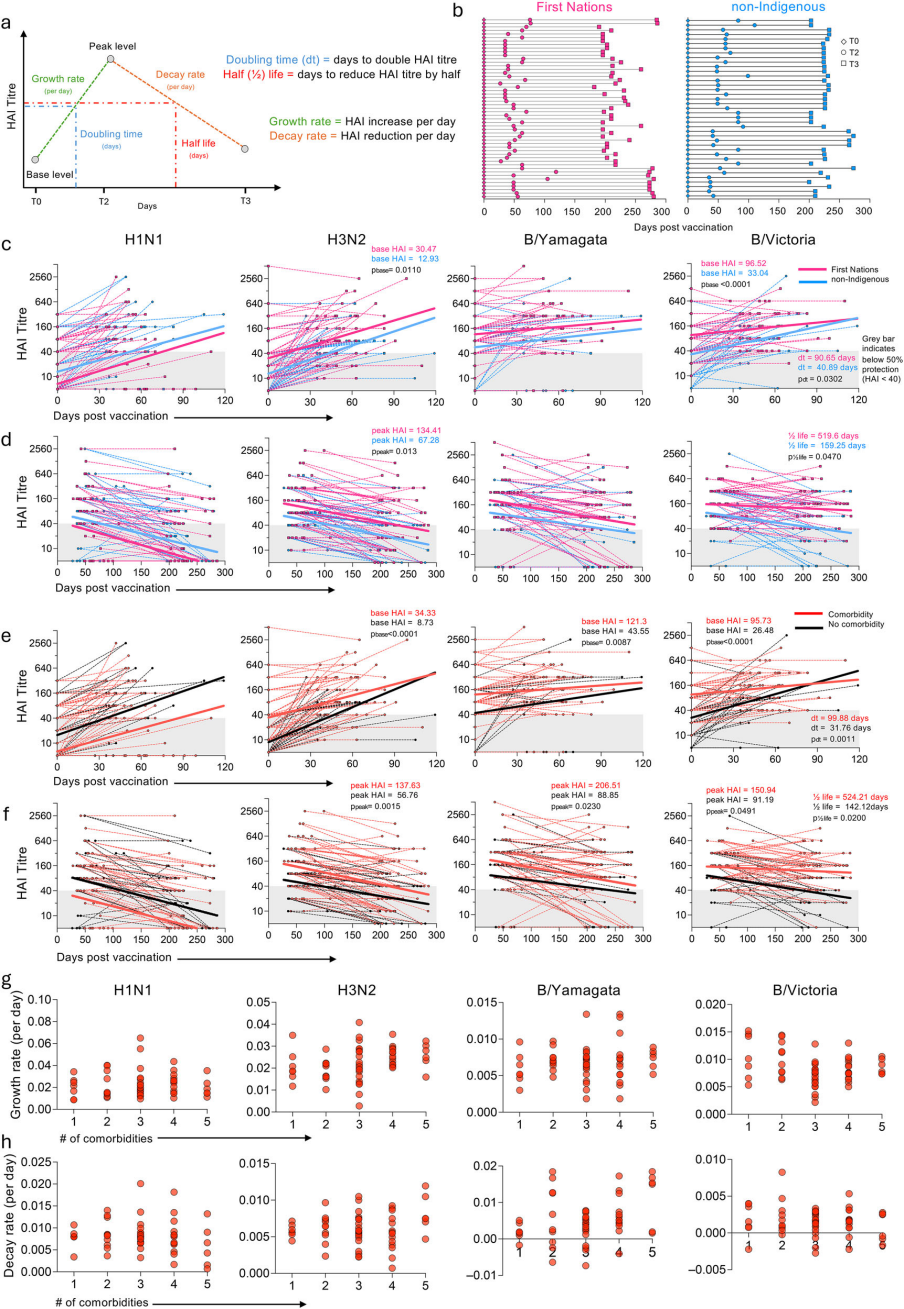
Comorbidity-affected First Nations and non-Indigenous vaccinees display stable long-term HAI kinetics. **a** Schematic depicting HAI kinetics modelling parameters in relation to vaccination sampling timepoints. **b** Longitudinal HAI cohort sampling timepoints (*n* = 90), overview of First Nations (pink, left, *n* = 51) and non-Indigenous (blue, right, *n* = 39) participants. **c**,** d** Comparison of First Nations and non-Indigenous HAI titre kinetics between (**c**) T0 and T2, d) T2 and T3 toward H1, H3, B/Yamagata and B/Victoria (left-to-right) vaccine components. **e**,** f** Comparison of HAI titre kinetics of those with (red, *n* = 57) and without (black, *n* = 33) comorbidities between **e** T0 and T2, **f** T2 and T3 toward H1, H3, B/Yamagata and B/Victoria vaccine components. Bolded lines depict (**c**,** e**) growth and (**d**,** f**) decay linear mixed-effect models for each subgroup. **g**,** h** Comparison of vaccine-component specific antibody (**g**) growth and (**h**) decay rates (per day) among participants with one or more comorbidities (*n* = 57). Dashed lines (**c–f**) connect paired data points of an individual vaccinee. Data points above grey bar indicates HAI titre of $\ge$40 which confers 50% serological protection. Statistical significance (**c–f**) of model parameters (base HAI level, doubling time (dt), peak HAI level and half-life (1/2 life)) was determined using Wald’s test, based on the standard errors of the estimated coefficients. Correlations calculated using two-sided Spearman correlation. Significant digits displayed on plots.

We also investigated durability of vaccine-specific HAI responses in First Nations and non-Indigenous participants at 6–9 months (T3; 182-286 days) post-IIV (Fig. 4a, b, *n* = 90 vaccinees). Consistent with analysis of the total cohort, we observed increased HAI titres between T0 and T2 for H1N1, H3N2 and B/Victoria, however we also found reduced titres between T2 and T3 for all vaccine components apart from B/Victoria in First Nations people (Supplementary Fig. 6a). T3 titres were similar to baseline (T0) titres, with no significant titre changes in First Nations or non-Indigenous vaccinees, suggesting post-IIV antibody titres return to baseline levels overtime (Supplementary Fig. 6a). Comparison of HAI kinetics between First Nations and non-Indigenous participants highlighted higher mean H3N2- (30.47) and B/Victoria-specific (96.52) HAI titres at baseline in First Nations participants compared to non-Indigenous individuals (H3N2 = 12.93, p = 0.011; B/Victoria=33.04, *p* < 0.0001) (Fig. 4c), consistent with analysis of the overall cohort (Fig. 3b). Despite lower baseline B/Victoria titres, non-Indigenous participants displayed a faster B/Victoria-HAI doubling time (40.89 days) compared to First Nations vaccinees (90.65 days, *p* = 0.03), leading to comparable peak titres (Fig. 4c). We observed higher mean peak H3N2 titres in First Nations participants (134.41) compared to non-Indigenous (67.28, *p* = 0.013) (Fig. 4d). However, models of H1N1, H3N2 and B/Yamagata HAI decay were comparable among First Nations and non-Indigenous participants (Fig. 4d). Although similar peak titres, First Nations participants had greater B/Victoria-specific HAI half-life (519.6 days) compared to non-Indigenous participants (159.2 days, *p* = 0.047), inferring greater durability of B/Victoria HAI responses in First Nations people.

Given the high proportion of First Nations individuals with comorbidities, we also examined the impact of comorbidities on HAI kinetics. Grouping by comorbidity status showed similar kinetics with stronger statistical results (Fig. 4e, f). Participants with comorbidities exhibited higher mean baseline titres toward H3N2 (34.33, *p* < 0.0001), B/Yamagata (121.3, *p* = 0.0087) and B/Victoria (95.73, *p* < 0.0001) vaccine components compared to individuals without comorbidities (8.73, 43.55 and 26.48, respectively) (Fig. 4e). Participants without comorbidities had robust B/Victoria HAI doubling time (31.76 days) compared to vaccinees with comorbidities (99.88 days, *p* = 0.0011) (Fig. 4e). Despite faster doubling time, vaccinees without comorbidities displayed lower B/Victoria-specific peak titres (91.19, *p* = 0.049) and half-life (142.12 days, *p* = 0.02), compared to those with comorbidities (peak HAI = 150.94, ½ life=524.21 days) (Fig. 4f). Individuals with comorbidities also had higher mean peak titres against H3N2 (137.63, *p* = 0.0015) and B/Yamagata (206.51, *p* = 0.023) compared to no comorbidities (56.76 and 88.85, respectively) (Fig. 4f). We also investigated the impact of multiple comorbidities on strain-specific HAI titre growth and decay rates. However, when considering both First Nations and non-Indigenous participants with one or more comorbidities, we found no significant correlations between HAI kinetic parameters with number of comorbidities towards any vaccine component (Fig. 4g, h; Supplementary Fig.6b).

### HA-specific IgD^-^CD10^-^ B cell populations increase following vaccination

**Fig. 5:**
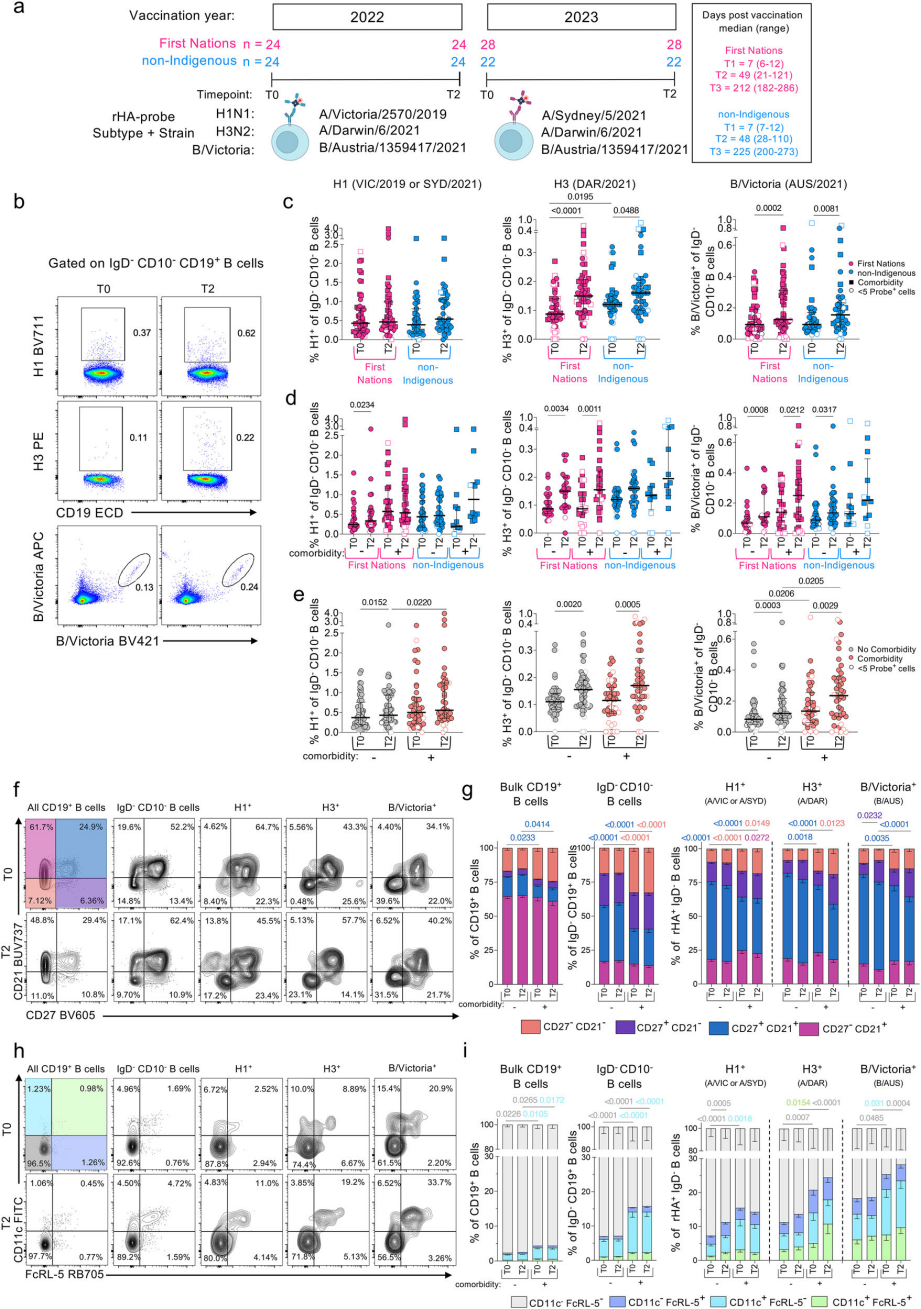
Influenza HA-specific IgD^-^CD10^-^ B cells increase post-vaccination in all vaccinees but phenotype is delineated by comorbidities. **a** Schematic depicting subsets of First Nations and non-Indigenous vaccinees selected (*n* = 98) for recombinant haemagglutinin (rHA) tetramerized probe staining. rHA probes were strain-specific and correspond to applicable (2022 or 2023) seasonal quadrivalent influenza vaccination formulations received for each participant. Created in BioRender. Skinner, M. (https://BioRender.com/7wicxf8). **b** Representative flow cytometric plots of vaccine-specific rHA^+^ IgD^-^ B cell gating at pre- (T0) and post-vaccination (T2) timepoints. **c**–**e** Frequencies of H1- (left), H3- (middle) and B/Victoria-specific (right) IgD^-^CD10^-^ B cells at T0 and T2 post-IIV for c First Nations (*n* = 52) and non-Indigenous (*n* = 46), **d** with (+) (n_FN_=30, n_NI_ = 12) and without (-) (n_FN_=22, n_NI_ = 34) comorbidities and (**e**) overall comorbidities (red, *n* = 42) and non-comorbidity (grey, *n* = 56) regardless of ethnicity. Participants that recorded <5 rHA probe^+^ B cells are represented as open symbols. **f** Representative flow cytometry plots and (**g**) proportions of CD27/CD21 phenotypic populations of bulk CD19^+^, total IgD^-^CD10^-^ and rHA-specific IgD^-^CD10^-^ CD19^+^ B cells in participants with and without comorbidities. Participants that recorded $\ge$5 rHA probe^+^ B cells were included this analysis, which allowed inclusion of 76–90% of participants across rHA-probe specificities and sampling timepoints (H1_T0_ = 87/98 (88%), H1_T2_ = 88/98 (90%), H3_T0_ = 76/98 (78%), H3_T2_ = 82/98 (84%), B/Vic_T0_ = 74/98 (76%), B/Vic_T2_ = 82/98 (84%)) to be assessed for phenotype. **h** Representative flow cytometry plots showing atypical marker gating on bulk CD19^+^, IgD^-^CD10^-^ and rHA-specific IgD^-^CD10^-^ CD19^+^ B cells. **i** Proportions of atypical B cell marker (FcRL-5 and CD11c) expression of bulk CD19^+^, total IgD^-^CD10^-^ and rHA-specific IgD^-^CD10^-^ CD19^+^ B cells in participants with and without comorbidities. Participants that recorded $\ge$5 rHA probe^+^ B cells were included in this analysis. **c**–**e** Bolded line represents median and error bars depict IQR. Statistical significance was determined by two-sided Wilcoxon ranked sum test for paired pre- and post-vaccination rHA^+^ frequencies within groups, and two-sided Mann-Whitney test for unpaired comparison between groups corrected with Dunn’s test for multiple comparisons. **g**, **i** Mean and SEM are shown, with statistical significance determined by two-way ANOVA with two-sided Tukey’s test for multiple comparisons for phenotypic B cell population frequencies between sample timepoints and participants.

To define influenza-specific cellular responses, we measured populations of HA-specific IgD^-^ CD10^-^ CD14^-^ CD16^-^ CD3^-^ CD19^+^ B cells (enriching for mature B cells that have undergone class-switch recombination, hereby referred to as IgD^-^CD10^-^ B cells) at T0 and T2 post-IIV using recombinant HA (rHA) probes directed against H1, H3 and B/Victoria vaccine components for 2022 and 2023 influenza vaccination seasons (Fig. 5a,b; Supplementary Fig. 7a; *n* = 98 vaccinees). First Nations participants had a lower frequency of H3 rHA-specific IgD^-^CD10^-^ B cells (median 0.087%) compared to non-Indigenous participants (0.12%, *p* = 0.0195) prior to vaccination (Fig. 5c). However, following IIV, we detected increased frequencies of H3 and B/Victoria rHA^+^ IgD^-^CD10^-^ B cells in First Nations and non-Indigenous vaccinees compared to baseline (Fig. 5c). When First Nations and non-Indigenous participants were stratified according to their comorbidity status, the increase in H3 and B/Victoria rHA^+^ IgD^-^CD10^-^ B cells post-IIV was maintained in First Nations people with and without comorbidities, while only B/Victoria rHA-specific IgD^-^CD10^-^ B cells increased in non-Indigenous participants without comorbidities (Fig. 5d). First Nations people without comorbidities also showed increased H1 rHA^+^ IgD^-^CD10^-^ B cells following vaccination. Finally, when participants were grouped only by comorbidity status, vaccinees had increased frequencies of H3- and B/Victoria-specific IgD^-^CD10^-^ B cells post-vaccination, while only vaccinees without comorbidities expanded H1-specific IgD^-^CD10^-^ B cells (Fig. 5e). Notably, those with comorbidities recorded higher median post-vaccination frequencies of H1- (0.56%) and B/Victoria-specific (0.24%) B cells compared to those without comorbidities (0.43%, *p* = 0.022 and 0.12%, *p* = 0.0205, respectively) (Fig. 5e).

### Prominent atypical CD27^-^CD21^-^ phenotype in HA^-^specific IgD^-^CD10^-^ B cells in individuals with comorbidities

To understand the impact of comorbidities on the phenotype of influenza rHA-specific IgD^-^CD10^-^ B cells, we measured expression of activation (CD21) and memory (CD27) phenotypic markers (those with $\ge$5 rHA^+^ IgD^-^ B cells were included in the phenotypic analysis, which permitted 76-90% sample retention across antigen-specificities and timepoints) (Fig. 5f; and Supplementary Fig. 7b). Individuals with comorbidities had decreased frequencies of CD27^+^CD21^+^ resting phenotype within influenza-specific rHA^+^ IgD^-^CD10^-^ CD19^+^ B cells (as well as IgD^-^CD10^-^ CD19^+^ B cells) at both baseline and T2 post-IIV (Fig. 5g, and Supplementary Fig.7c). Conversely, the proportion of CD27^-^CD21^-^ B cells was increased in participants with comorbidities for H1-specific and total IgD^-^CD10^-^ B cells at both timepoints, as well as H3-specific B cells post-IIV (Fig. 5g). We observed similar trends among First Nations and non-Indigenous participants with comorbidities (Supplementary Fig. 7c). A growing body of literature suggests IgD^-^CD27^-^CD21^-^ are atypical lineage B cells (atBCs) associated with chronic diseases, autoimmunity and aging^47–57^. In participants without comorbidities, only CD27^+^CD21^-^ activated B/Victoria-specific IgD^-^CD10^-^ B cells increased post-vaccination (Fig. 5g). As expected, bulk CD19^+^ B cells were predominantly CD27^-^CD21^+^ (mean 61.2% - 65.3%) at all timepoints, reflecting primarily naïve IgD^+^ B cells (Fig. 5g; Supplementary Fig. 7b).

We also investigated the isotype of antigen-specific B cell responses by measuring expression of IgG and IgM (Supplementary Fig. 8a). We found an increased proportion of IgG^+^ H3-specific B cells following vaccination, while the proportion of IgG^+^ B/Victoria-specific B cells decreased, in participants with comorbidities (Supplementary Fig. 8b, c). H3-specific B cells had a lower proportion of IgG^+^ cells compared to H1- and B/Victoria-specific B cells, with subsequently greater proportions of IgM^+^ (compared to H1 only) and IgD^-^ IgG^-^ IgM^-^ B cells (Supplementary Fig. 8 d).

### Influenza rHA-specific B cells in vaccinees with comorbidities display a prevalent atypical B cell phenotype

To further investigate the high prevalence of atypical lineage rHA^+^ IgD^-^CD10^-^ B cells in participants with comorbidities at baseline and post-IIV, we analysed expression of known atBC markers CD11c and FcRL-5^47,48,58,59^ on IgD^-^CD10^-^ rHA-specific B cells as well as on total IgD^-^CD10^-^ and bulk CD19^+^ B cells (Fig. 5h). Our data revealed B cells expressing CD11c and/or FcRL-5 atypical surface markers were CD21^-^ and predominantly CD27^-^, as described for single-positive FcRL-5 and CD11c atBC populations^49,58^ (Supplementary Fig. 8e). To validate the identity of FcRL-5 and CD11c expressing B cells in our cohort as atypical lineage memory B cells, we confirmed co-expression of FcRL-5 and CD11c markers with expression of transcription factor T-bet (Supplementary Fig. 8 f), an atBC population marker^48,50,58,60–63^. This established that T-bet^+^FcRL-5^+^ IgD^-^CD10^-^ B cells in our vaccinees display the CD19^high^ CD11c^+^ CD21^-^ and CD27^-/+^ phenotype, consistent with published work in the context of influenza-vaccination and other disease settings^48,50,58,60–63^.

CD11c and FcRL-5 expression was enriched on influenza rHA-specific IgD^-^CD10^-^ B cells as well as total IgD^-^CD10^-^ B cells in comparison to the bulk CD19^+^ B cell population (Fig. 5i, and Supplementary Fig. 8g). We observed higher expression of atBC markers in participants with comorbidities compared to individuals without comorbidities, exemplified by lower frequencies of double-negative CD11c^-^FcRL-5^-^ rHA-specific IgD^-^CD10^-^ B cells, total IgD^-^CD10^-^ and bulk CD19^+^ B cells in participants with comorbidities (H1-specific B cells at T0 only, H3- and B/Victoria-specific B cells at T0 and T2) (Fig. 5i, Supplementary Fig. 8h). This can be attributed to higher T0 or T2 frequencies of CD11c^+^ B cells in participants with comorbidities (Fig. 5i, and Supplementary Fig. 8h). We also observed a higher proportion of CD11c^+^FcRL-5^+^ H3-specific B cells post-IIV in participants with comorbidities compared to individuals without pre-existing conditions. We detected similar trends among First Nations and non-Indigenous participants with comorbidities (Supplementary Fig. 8h). The expression of atypical markers by IgD^-^CD10^-^ B cells was stable following vaccination, with the only change depicting lower proportions of CD11c^-^FcRL-5^-^ H1-specific B cells post-vaccination in participants without comorbidities (Fig. 5i). Thus, our data demonstrate increased prevalence of atBCs within influenza HA-specific IgD^-^CD10^-^ CD19^+^ B cell populations in individuals with comorbidities, further corroborating an altered differentiation status with distinct features^47,48,58–60,64,65^ of memory B cells, including HA-specific B cells, in people with chronic diseases. These data provide in-depth evidence of atBC populations in our vaccinees at the protein level with multi-marker resolution.

### Reduced cT_FH_1 cell activation during early IIV response in individuals with comorbidities

**Fig. 6:**
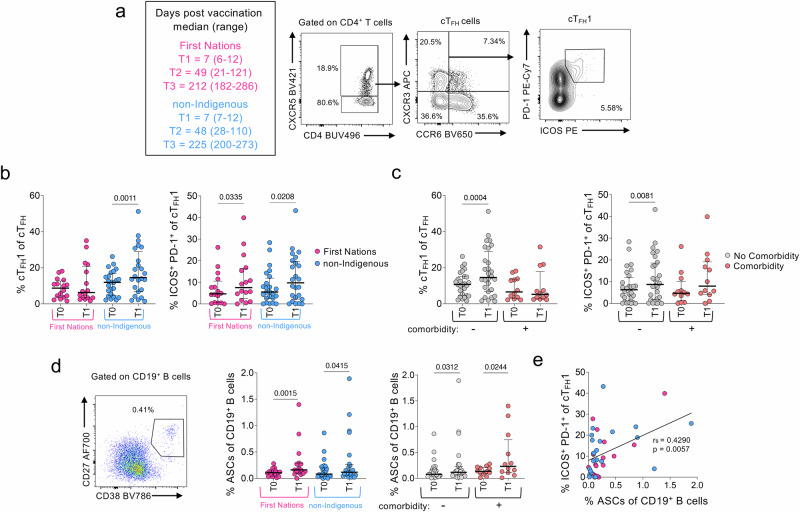
Early recruitment of activated ICOS^+^ PD-1^+^ cT_FH_1 cells post-IIV is insignificant in those with comorbidities. **a** Representative flow cytometry plots depicting gating of CXCR5^+^ cT_FH_ CD4^+^ T cells (left), CXCR3^+^ CCR6^-^ cT_FH_1 cells (middle) and ICOS^+^PD-1^+^ activated CXCR3^+^ cT_FH_1 cells (right). **b**, **c** Proportions of CXCR3^+^ cT_FH_1 (left) and ICOS^+^PD-1^+^ activated CXCR3^+^ cT_FH_1 cells (right) pre- and post-IIV according to (**b**) ethnicity (n_FN_=15, n_NI_ = 23) and (**c**) comorbidity status regardless of ethnicity (n_NCM_=27, n_CM_ = 11). **d** Representative flow cytometry plot (left) and proportions of CD27^high^CD38^high^ antibody-secreting CD19^+^ B cells according to ethnicity (middle) and comorbidities regardless of ethnicity (right). **e** Spearman correlation of T1 ASCs and ICOS^+^PD-1^+^ cT_FH_1 cells where symbols (*n* = 38) indicate First Nations (pink) and non-Indigenous (blue) participants. **b**–**d** Bolded lines represent median and errors bars show IQR with statistical significance determined by two-sided Wilcoxon ranked sum test for paired and post-IIV frequencies within groups and two-sided Mann-Whitney test for unpaired comparison between groups corrected with Dunn’s test for multiple comparisons. Correlations (**e**) calculated using two-sided Spearman correlation.

Having defined influenza-specific antibody and memory B cell responses at T2 post-IIV, we subsequently assessed early correlates of vaccine-induced antibody responses at T1 (6-12 days post-IIV, *n* = 40 vaccinees) (Fig. 6a). Previous studies by us and others found activated ICOS^+^PD-1^+^ circulating CXCR3^+^ (c)T_FH_1 cells and CD27^high^CD38^high^ antibody-secreting B cells (ASCs), generated 7–10 days post-influenza virus infection or vaccination, positively correlate with day 28 HAI titres^16,66^. cT_FH_ cells are phenotypically and functionally similar to germinal centre (GC) T_FH_ cells, which reside in secondary lymphoid organs and facilitate generation of high-affinity memory B cells in GCs^67–69^. Our data show that CXCR3^+^ cT_FH_1 cell frequency increased in non-Indigenous participants but not in First Nations people at T1 post-IIV (Fig. 6b). Although differences in cT_FH_1 cell frequency of total cT_FH_ cells, First Nations and non-Indigenous vaccinees had robust increases in activated ICOS^+^PD-1^+^ CXCR3^+^ cT_FH_1 cells at T1 post-IIV (Fig. 6b).

Further stratification of vaccinees according to comorbidities revealed only participants without comorbidities exhibited increased frequency of CXCR3^+^ cT_FH_1 cells at T1, compared to pre-vaccination (Fig. 6c). Similarly, only individuals without comorbidities had increased proportions of activated ICOS^+^PD-1^+^ CXCR3^+^ cT_FH_1 cells (Fig. 6c). Thus, participants with comorbidities had diminished cT_FH_1 cell responses post-IIV in terms of both frequency and activation. As First Nations participants in our cohort had markedly increased prevalence of comorbidities (and overrepresentation of multimorbidity), this suggests suboptimal cT_FH_1 responses are driven by comorbidities rather than ethnicity.

In terms of ASC responses and comorbidities, both participants with and without comorbidities displayed increased ASCs at T1 (Fig. 6d). We also observed a positive correlation between ASCs and activated cT_FH_1 cells in our overall vaccine cohort (Fig. 6e).

### Multimorbidity-associated inflammation at baseline associated with influenza-specific atypical B cell phenotypes

**Fig. 7:**
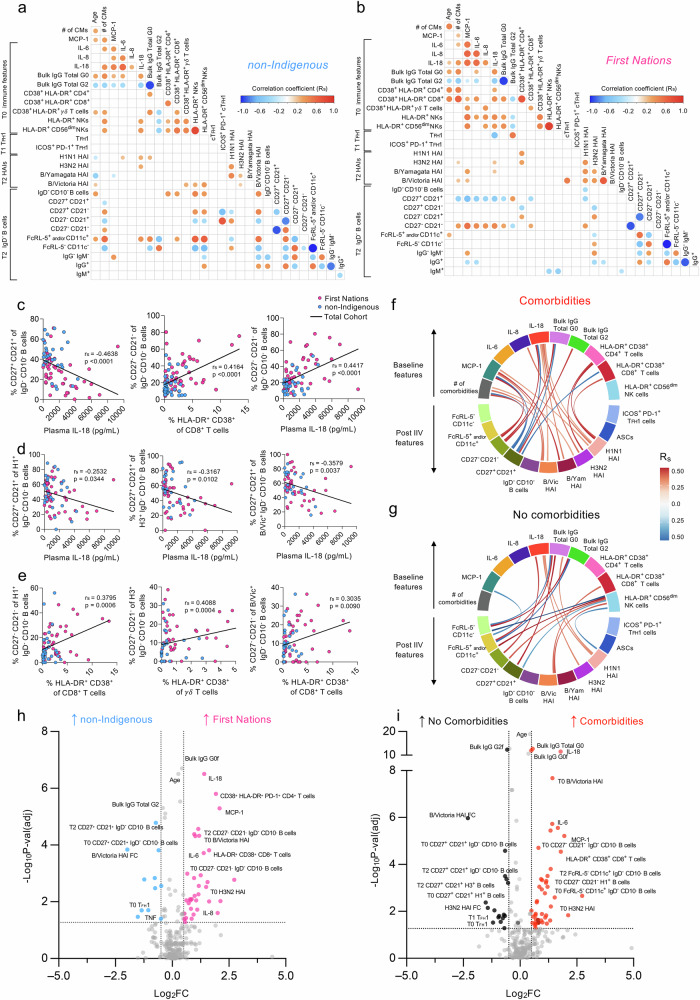
Multimorbidity-associated inflammatory signatures at baseline are associated with atypical influenza-specific IgD^-^CD10^-^ B cell phenotypes following IIV. **a**, **b** Correlation matrix of spearman correlations between baseline inflammatory features and influenza-specific humoral immune responses in (**a**) non-Indigenous and (**b**) First Nations vaccinee subpopulations. Only statistically significant correlations (*p* < 0.05) are displayed. **c**–**e** Representative plots of significant Spearman correlations between (**c**) CD27/CD21 phenotype of total IgD^-^ (CD27^+^CD21^+^ (left^)^ or CD27^-^CD21^-^ IgD^-^ B cells ^(^middle and right)) vs baseline inflammatory features (IL-18 or HLA-DR^+^CD38^+^ CD8^+^ T cells)(*n* = 80). **d**, **e** Representative plots of significant Spearman correlations between (**d**) CD27^+^CD21^+^ HA-specific B cells vs plasma IL-18 and (**e**) CD27^-^CD21^-^ HA-specific B cells and activated T cells (HLA-DR^+^CD38^+^ CD8^+^ or $\gamma \delta$ T cells) (*n* = 75). **f**, **g** Circos plots depicting Spearman correlations between baseline inflammatory signatures and features of post-IIV humoral immunity in (**f**) vaccinees with comorbidities and (**g**) those without comorbidities. Only significant correlations (*p* < 0.05) are shown. Colour of bands connecting immune features represent the strength of the correlation indicated by the Spearman correlation coefficient. **h**, **i** Volcano plots showing direct comparison of 293 unique pre- and post-vaccination immune features between (**h**) First Nations and non-Indigenous vaccinees and (**i**) those with and w**i**thout comorbidities regardless of ethnicity. Correlations calculated using two-sided Spearman correlation. Statistical significance (**h**, **i**) was determined with an unpaired, two-sided *t*-test with Benjamini–Hochberg adjustment.

To evaluate the impact of baseline immune features on post-IIV influenza-specific immunity, we generated a correlation matrix assessing the links between key baseline inflammatory features, HAI antibody titres, early cT_FH_1 activation and memory B cell phenotypes (Fig. 7). We observed distinct correlative relationships between rHA^+^ IgD^-^CD10^-^ memory B cell phenotypes and baseline inflammatory signatures (Fig. 7a,b, Supplementary Fig. 9). In both non-Indigenous and First Nations groups, the number of comorbidities positively correlated with plasma IL-18 (non-Indigenous r_s_ = 0.416, *p* < 0.0001; First Nations r_s_ = 0.377, *p* < 0.0001), agalactosylated G0 IgG antibodies (non-Indigenous r_s_ = 0.423, *p* < 0.0001; First Nations r_s_ = 0.432, *p* < 0.0001) and post-vaccination proportions of CD27^-^CD21^-^ atypical lineage IgD^-^CD10^-^ B cells (non-Indigenous r_s_ = 0.322, *p* = 0.029; First Nations r_s_ = 0.356, *p* = 0.0095) (Fig. 7a, b). Plasma levels of IL-18 also inversely correlated with T1 frequency of cT_FH_1 cells in First Nations people (r_s_ = -0.565, *p* = 0.026). Interestingly, T2 proportions of FcRL-5^+^ and/or CD11c^+^ IgD^-^CD10^-^ B cells positively correlated with the number of comorbidities in non-Indigenous vaccinees only (r_s_=0.588, *p* < 0.0001) (Fig. 7a).

IL-18 levels and activated CD8^+^ T cell frequencies correlated with post-vaccination expansion of CD27^-^CD21^-^ IgD^-^CD10^-^ B cells within the full cohort (r_s_=0.4417, *p* < 0.0001 and r_s_ = 0.4164, *p* < 0.0001, respectively) (Fig. 7c). These results were sustained across all three rHA^+^ populations (Fig. 7d). Notably, increasing concentrations of plasma IL-18 correlated with the reduction of CD27^+^CD21^+^ B cells for H1^+^ (r_s_ = -0.2532, *p* = 0.0344), H3^+^ (r_s_ = -0.3167, *p* = 0.0102) and B/Victoria^+^ (r_s_ = -0.3579, *p* = 0.0037) IgD^-^CD10^-^ B cells (Fig. 7d). Consistent with findings for total IgD^-^CD10^-^ B cells, frequency of activated CD8^+^ and ${{{\rm{\gamma }}}}{{{\rm{\delta }}}}$ T cells at baseline also correlated with the expansion of CD27^-^CD21^-^ antigen-specific B cells (Fig. 7e, and Supplementary Fig. 9).

In terms of comorbidities, positive correlations of CD27^-^CD21^-^ IgD^-^CD10^-^ B cells proportions at T2 with plasma IL-18, MCP-1, agalactosylated G0 IgG and number of comorbidities were maintained in comorbidity-affected individuals (Fig. 7f). We also detected negative correlations of CD27^+^CD21^+^ IgD^-^CD10^-^ B cells at T2 with these same features, in addition to CD38^+^HLA-DR^+^ CD8^+^ T cells in individuals with comorbidities and only HLA-DR^+^ CD56^dim^ NK cells in vaccinees without comorbidities (Fig. 7f, g). Additionally, in individuals without comorbidities HLA-DR^+^ CD56^dim^ NK cells and agalactosylated pro-inflammatory IgG correlated with proportions of FcRL-5^+^ and/or CD11c^+^ IgD^-^CD10^-^ B cells (Fig. 7g). To confirm our comorbidity-related findings were not driven by age, we compared key findings (IL-8, IL-18, IgG G0 and atypical B cells) between paired age groups of comorbidity-stratified cohorts. This analysis highlighted that while there are trends of increased inflammation and perturbed immunity with age, the presence of comorbidities significantly increased the magnitude of these features within distinct age groups (Supplementary Fig. 10)

Direct comparison of 293 unique pre- and post-vaccination immune features between ethnicity groups showed agalactosylated G0 IgG antibodies, plasma cytokines (IL-6, IL-8, MCP-1 and IL-18), activated T cells, atypical lineage CD27^-^CD21^-^ memory B cells and increased H3N2 and B/Victoria baseline HAI titres were highly prominent in First Nations people compared to the non-Indigenous group (Fig. 7h). Additionally, non-Indigenous participants had higher anti-inflammatory G2 IgG, T_FH_1 frequencies, H3N2 and B/Victoria HAI fold changes and proportions of resting CD27^+^CD21^+^ IgD^-^CD10^-^ B cells (Fig. 7h). Comparisons between comorbidity and non-comorbidity groups revealed very similar distribution of immune features as ethnicity-stratified analysis (Fig. 7i). These data further suggest that while First Nations and non-Indigenous vaccinees generated prototypical humoral immune responses to seasonal IIV, the occurrence of comorbidities, which were highly prevalent in our First Nations cohort, contributed to baseline inflammation, agalactosylated G0 IgG antibodies, cellular hyperactivation, and atypical lineage CD27^-^CD21^-^ memory B cells following vaccination.

## Discussion

Our study provides key insights into immune responses toward seasonal IIV in Australian First Nations people with high prevalence of comorbidities. Our findings demonstrate pre-existing humoral immunity and comorbidity-associated inflammatory signatures shape responses towards seasonal influenza vaccination. Our cohort had a high prevalence of comorbidities, presenting a rare opportunity to define baseline immunological features impacting humoral immunity following vaccination in First Nations as well as non-Indigenous people. Indigenous people globally are at increased risk of developing both severe viral respiratory infections^1–5^ and chronic diseases^19–21^. It is therefore of key importance to understand in-depth the impact of comorbidities on immunity towards influenza vaccination in such high-risk groups. We identified comorbidity-driven inflammatory signatures (pro-inflammatory cytokines MCP-1, IL-6 and IL-18, inflammation-associated agalactosylated IgG antibodies and activated NK cells, ${{{\rm{\gamma }}}}{{{\rm{\delta }}}}$, CD4^+^ and CD8^+^ T cells) at baseline, associated with a systemic atypical B cell phenotype which extended to influenza-specific B cell populations, and lack of significant cT_FH_1 cell activation in individuals with comorbidities following influenza vaccination.

Our previous work examining SARS-CoV-2 mRNA vaccine responses in First Nations people linked pro-inflammatory agalactosylated IgG antibodies and high plasma IL-18 levels to a reduced humoral axis, inversely correlating with RBD-specific IgG titres, spike-specific memory B cells and T_FH_ cell frequencies^17^. Our current influenza vaccination study additionally revealed comorbidity-associated inflammatory signatures in both First Nations and non-Indigenous participants were more prominent with increasing multimorbidity. Participants with comorbidities exhibited increased concentrations of pro-inflammatory plasma cytokines and chemokines compared to participants without comorbidities. Specifically, we observed over-representation of interferon (IFN)-$\gamma$ inducing factor, IL-18, in individuals with comorbidities, that was highest in those with multimorbidity. In combination with IL-12, IL-18 is a potent activator of NK cells, non-polarised T cells, T-helper type 1 (T_H_1), dendritic and B cells^70^. High plasma levels of IL-18 are associated with influenza and SARS-CoV-2 infection, as well as conditions such as atheroma, myocardial infarction, chronic obstructive pulmonary disease and Crohn’s disease^26,67,70–73^. It is thus unsurprising that high levels of this cytokine correlated with increased baseline NK and T cell activation. Additionally, we found IgG glycosylation profiles were characterised by a higher abundance of pro-inflammatory agalactosylated glycans in First Nations and non-Indigenous individuals with comorbidities. Higher levels of agalactosylated IgG antibodies have been associated with seroconversion following influenza vaccination^28^. We observed no such relationship in our cohort, most likely due to high baseline antibody titres in individuals with comorbidities.

Despite identifying a pro-inflammatory baseline immune profile in our participants with comorbidities, we did not observe comorbidity-associated reduced antibody titres following influenza vaccination. Unlike SARS-CoV-2 mRNA vaccination, which served as a primary antigen exposure for most individuals in Australia and required multiple doses for protective responses, seasonal IIVs build on strong foundations of pre-existing influenza-virus-specific antibody immunity, best demonstrated in our cohort by seroprotective HAI titres and detectable frequencies of HA-specific memory B cells, even prior to vaccination. Reduced antibody levels identified in SARS-CoV-2 vaccinees with comorbidities^17^ may be linked to immune naivety toward a novel antigen, exacerbated by a hyperactivated baseline immune profile. In fact, in our influenza vaccinees with comorbidities, we found high levels of pre-existing influenza-specific immunity, potentially due to repeated seasonal vaccinations and/or recent infections with related strains, however concordant epidemiological or immunological data is not available to confirm either hypothesis. Despite comparable antibody levels and ASCs in vaccinees with or without comorbidities, we found that post-IIV, individuals with comorbidities had increased influenza-specific atypical memory B cells and suboptimal cT_FH_1 responses, inferring potential modulation of the humoral axis that could be overlooked due to seroprotective post-IIV HAI titres. HAI titres are the gold-standard correlate of protection endorsed by the WHO, however it is a largely quantitative measure that provides limited information on the quality and effectiveness of humoral responses (isotype, subclass, neutralisation, Fc function). Therefore, we believe our findings of atypical memory phenotypes and altered cT_FH_1 responses warrant further investigation.

Additionally, vaccinees with comorbidities had higher proportions of High Non-Responder individuals (baseline HAI $\ge$40 but no 4-fold HAI increase post-IIV) against H1N1, H3N2 and B/Victoria vaccine components compared to those without comorbidities. These data again demonstrate a high-level of pre-existing influenza immunity in comorbidity-affected people but also flag a potential perturbation of vaccine responses due to increased failure to seroconvert. Perhaps, in the event of a pandemic influenza virus, where pre-existing influenza-specific immunity would provide only limited protection^74–76^, individuals with attributes of perturbed baseline immunity (agalactosylated IgG, pro-inflammatory cytokines, and high immune cell activation) would exhibit impaired humoral responses toward vaccination with a novel antigen. Future studies into these high-risk populations to investigate the quality and functionality of antibody responses and the memory B cell repertoire would be of interest to public health.

Analysis of HAI titres over time revealed largely comparable strain-specific HAI kinetics (growth, decay, doubling time and half-life) between First Nations and non-Indigenous vaccinees with or without comorbidities, despite higher baseline HAI titres for some influenza vaccine components in First Nations and comorbidity affected participants. In our participants, vaccine-specific HAI titres reached serological protective titres at 1-3 months post-vaccination (T2), however 6-9 months (T3) titres were remarkably similar to pre-vaccination (T0) titres. This suggests that while seasonal vaccination boosts humoral immunity towards vaccine strains for the current season, it may not contribute to increasing baseline influenza-specific humoral immunity for the following season, even when the strains remain the same. Alternatively, seasonal influenza vaccination may be crucial for increasing humoral protection during a season, and equally critical for the maintenance of baseline influenza-specific humoral immunity. While antibody maintenance and effects of repeated vaccination have been studied^77^, further investigation into the maintenance of influenza-specific immunity in high-risk groups is needed.

While our data demonstrate comparable frequencies of HA-specific IgD^-^CD10^-^ B cells among First Nations and non-Indigenous vaccinees with and without comorbidities, functional phenotypes of IgD^-^CD10^-^ B cells were influenced by comorbidities and correlated with features of perturbed baseline immunity. Vaccinees with comorbidities exhibited higher frequencies of atypical lineage CD27^-^CD21^-^ IgD^-^ and rHA-specific IgD^-^CD10^-^ B cells compared to those without comorbidities. These results were recapitulated using atBC phenotypic markers CD11c and FcRL-5, showing individuals without comorbidities displayed reduced overall expression of atBC markers at both sampling timepoints (T0 and T2). While the precise physiologic role of atBCs remains incompletely understood, evidence suggests atBCs are associated with chronic infections in malaria, HIV and HCV infection, autoimmunity and aging^47–57^. Interestingly, human and mice studies show that without CD21 on the surface, B cell receptor (BCR) activation thresholds are higher, signal transduction is muted and BCR-induced cellular proliferation is limited^49^. CD27^-^CD21^-^ atBCs also upregulate T cell interaction molecules^55^ and readily proliferate in response to TLR-7 and TLR-9 agonists, CD40L and cytokines, suggesting other modes of stimulation may induce activation for programmed differentiation^48,49,54,78^. Formation of atBCs is directed by expression of T_H_1 cytokines including IFN-$\gamma$, which can be induced by IL-18-mediated activation of the STAT4 signalling pathway on NK and T_H_1 cells^62,79^. Perhaps, the propensity for memory B cells to adopt an atBC phenotype in individuals with comorbidities is leveraged by an IL-18 dominated baseline cytokine milieu, as our data suggest. It is important to note atypical B cells (FcRL5^+^ and also CD21^low^) have been documented in the normal response to influenza vaccination in healthy adults^80^, with some contesting they are essential for a protective post-IIV response, although these studies either included comorbidity effected individuals or did not disclose participants’ comorbidity presentations^47,80,81^. It is likely that perturbed baseline immunity, identified in our study, contribute to shaping B cell fate. For example, IL-6 plays a role in B cell differentiation and can contribute to increased antibody production^82,83^. However, given we observed upregulation of multiple inflammatory cytokines and glycosylation biomarkers, including IL-18 and agalactosylated IgG within individuals with comorbidities, these signatures more likely reflect chronic inflammation. Indeed, a bidirectional feedback loop has been described between cytokines and antibody glycosylation patterns^33^, with cytokines affecting the expression of glycotransferases in B cells, thus altering the Fc glycosylation of newly produced antibodies^33,84^. These altered antibodies can subsequently activate innate immune cells, further impacting cytokine production, and hence potentially contributing to the maintenance of chronic inflammatory states.

Overall, our data defined in-depth immune responses towards inactivated influenza vaccination in Australian First Nations and non-Indigenous people with and without comorbidities. Our study demonstrated generation of seroprotective HAI titres in First Nations and non-Indigenous with comorbidities, revealing high baseline antibody levels in participants with comorbidities. Phenotypically, however, in individuals with comorbidities, influenza-specific B cells were of a prominent atypical phenotype, accompanied by the lack of significant cT_FH_1 activation, indicating qualitatively different humoral immunity towards influenza virus components in participants with chronic comorbidities. Our study thus provides immunological basis to support influenza vaccination for high-risk groups, highlighting the ability of First Nations and non-Indigenous individuals with comorbidities to induce influenza-specific serological and cellular responses, albeit of distinct B cell phenotype and cT_FH_1 characteristics, despite substantial baseline immune perturbation.

### Limitations of our study

In our study, we observed disparate comorbidity burden between groups: 63% of First Nations participants had ≥2 comorbidities, compared with 9% of non-Indigenous, clearly revealing disproportionally higher comorbidities in First Nations people. In addition, comorbidity affected participants were older, irrespective of ethnicity. While we demonstrated the presence of comorbidities significantly increased the magnitude of age-associated inflammation and perturbed immunity between subgroups of younger and older adults, these highly overlapping factors (comorbidity burden, ethnicity and age), inherently impacted our statistical analysis as they were not statistically independent variables that could be adjusted to assess their individual contributions to observed immunological differences. However, it is important to note a disparately high proportion of First Nations people globally are impacted by comorbidities at younger ages compared to non-Indigenous people, and our data provide key insights into why Indigenous people globally are highly susceptible to life-threatening influenza disease. We also assert the importance of highlighting Australian First Nations people in this study, as a severely understudied population with unique socioeconomic, political and health status. Our study displays some heterogeneity of post-vaccination sampling timepoints, due to logistics of collecting blood samples from First Nations people in remote and regional areas. In addition, our interrogation of early post-vaccination immune responses (ASCs and cT_FH_ cells) was limited to measuring global populations rather than influenza antigen specific. Partially due to reduced sample size and cell numbers at this optional timepoint, making a HLA-restricted tetramer-based approach impractical, but also due to the challenges of accurately characterising highly activated rHA-specific antibody secreting B cells via flow cytometry. In future studies, where sample size and cell numbers permit, measuring antigen-specific cT_FH_ cells and ASCs during early vaccination responses in comorbidity affected individuals would be informative.

## Methods

### LIFT-v study design and specimen collection

We enroled 344 participants who received an IIV (194 Australian First Nations and 150 non-Indigenous individuals) across 2022, 2023 and 2024 Southern Hemisphere influenza seasons, via the Menzies School of Health Research in Darwin, Northern Territory or University of Melbourne, Victoria, Australia. Samples of vaccinees were taken at T0 (*n* = 340), T1 (*n* = 41), T2 (*n* = 286) and T3 (*n* = 90). Demographic, clinical and sampling timepoint information of total LIFT-v cohort, First Nations and non-Indigenous groups are described in Supplementary Tables 1 and Supplementary Data 1. LIFT-v participants reported comorbidities, and participants recruited via the Menzies School of Health Research also gave consent to review their medical records. Evidence of the following comorbidities and factors were documented: chronic renal disease, cardiac disease, chronic respiratory disease, diabetes, liver disease, neurological disorders, obesity and immunosuppression. Peripheral blood samples were collected in heparinised tubes and serum tubes, with plasma and serum collected following centrifugation, respectively. Peripheral blood mononuclear cells (PBMCs) were isolated by Ficoll-Paque separation. Samples were processed at the Menzies School of Health Research or University of Melbourne and stored at the University of Melbourne. Experiments conformed to the principles of the Declaration of Helsinki (2013) and the Australian National Health and Medical Research Council Code of Practice. Written informed consent was obtained from all blood donors before the study. Participants recruited via the Menzies School of Health Research were reimbursed with vouchers up to the value of $20AUD for their time and inconvenience. The study was approved by the Human Research Ethics Committee of the Northern Territory Department of Health and the Menzies School of Health Research (no. 2021-3964) and the University of Melbourne Human Research Ethics Committees (nos. 21864, 31236).

### Plasma cytokine analysis

The LEGENDplex Human Inflammation Panel 1 kit (BioLegend) was used according to manufacturer’s instructions to measure cytokines and chemokines in participants baseline (T0) plasma (1:2 dilution). Cytokines and chemokines including IL-1β, IFNα2, IFN-γ, TNF, MCP-1 (CCL2), IL-6, IL-8 (CXCL8), IL-10, IL-12p70, IL-17A, IL-18, IL-23 and IL-33 were measured and concentrations (pg/mL) were calculated based on known standards. Samples were acquired on a LSR Fortessa (BD Biosciences) and analysed with the QOGNIT LEGENDplex programme.

### IgG purification and IgG N-linked glycan profiling

IgG antibodies were purified from donor plasma using the Melon Gel IgG Purification Kit (Thermo Fisher Scientific) according to the manufacturer’s protocol^17^. Excess serum proteins were removed and antibodies buffer exchanged into PBS by centrifugation through 100-kDa Amicon Ultra Centrifugal Filters (Merck Millipore). Sample purity was assessed via SDS–PAGE (Bio-Rad Laboratories) and approximate concentrations of IgG per sample was measured using a NanoDrop spectrophotometer (Bio-Rad Laboratories). N-linked glycosylation patterns were measured according to the ProfilerPro glycan profiling LabChip GXII Touch protocol on the LabChip GXII Touch HT Microchip-CE platform (PerkinElmer) using the LabChip GX Touch software (v.1.9.1010.0), as described previously^17^. Digested and labelled N-linked glycans were analysed by microchip capillary electrophoresis laser-induced fluorescence, with the relative prevalence of major N-linked glycan profiles of IgG analysed using the LabChip GX Reviewer (PerkinElmer) software. Peaks were assigned based on the migration of known standards and glycan digests. The peak area and relative abundance of each glycoform was calculated.

### Haemagglutinin inhibition assay

Donor serum collected on T0, T2 and T3 were RDE-treated and assessed for HA-antibody titres against virus strains specific to their relevant seasonal vaccine components (Supplementary Table 2). H3N2 viruses were titrated in the presences of Oseltamivir carboxylate on guinea pig red blood cells, while H1N1 and influenza B viruses were titrated without Oseltamivir carboxylate on turkey red blood cells. Influenza B viruses were ether treated before titration. HAI titration was performed for each sample until complete inhibition of haemagglutination was achieved, as described previously and as per WHO guidelines^16,46^. HAI titres were reported as the reciprocal of the highest dilution of serum where haemagglutination was completely inhibited.

### HAI kinetics modelling

To evaluate the baseline levels, peak responses, and the growth and decay rates of haemagglutination inhibition (HAI) titres following vaccination, we fitted linear mixed effects models. Each model included a fixed binary effect for participant group (First Nations vs. non-Indigenous) and random intercepts for individual participants to account for within-subject correlation. Left censoring was incorporated to appropriately handle HAI titre values falling below the assay’s lower limit of detection (HAI < 10). Statistical significance of model parameters was assessed using Wald’s test, based on the standard errors of the estimated coefficients. We also conducted a secondary analysis to compare growth and decay rates between individuals with and without reported comorbidities, using the same modelling framework. A group and time interaction term, which allowed both intercepts and slopes (growth and decay rates) to differ between First Nations and non-Indigenous participants was employed. Formal statistical testing of slope differences was performed using Wald tests on the interaction term in the linear mixed-effects models. All analyses were performed using the *GLMMadaptive* package in R (version 4.3.1).

### Immune profiling flow cytometric analysis

Cryopreserved PBMCs were thawed, cells (5.0 ×10^5^) were then stained with two immunophenotyping antibody staining panels (Supplementary Table 3 and 4), fixed in 1% PFA and washed in MACS buffer (PBS with 0.5% BSA and 2 mM EDTA) before acquisition. Samples were acquired on a LSR Fortessa (BD Biosciences) and analysed with FlowJo10 (FlowJo, LLC).

### Assessment of influenza HA-specific IgD^-^CD10^-^ B cells

Recombinant HA probes used in this analysis are biotinylated tetramerized HA molecules with a removed transmembrane domain and a mutation at Y98F (influenza A virus probes only) to prevent nonspecific binding to sialic acid residues on non-antigen specific B cells^85^. rHA probes were matched to vaccine components of each year (2022 and 2023). H1 (A/Victoria/2570/2019 for 2022, A/Sydney/5/2021 for 2023) and H3 (A/Darwin/6/2021 both years) virus strain rHA probes were each conjugated to a single fluorochrome (BV711 and PE respectively), while the B/Victoria lineage rHA probe (B/Austria/1359417/2021 both years) was conjugated to two fluorochromes (BV421 and APC) and B/Victoria rHA^+^ B cells gated on dual colour positivity. Cryopreserved PBMCs were thawed in cRPMI with Benzonase® Nuclease and cells (1.0 – 8.0 × 10^6^) stained with a B cell phenotyping panel and conjugated rHA probes (Supplementary Table 5). Cells were fixed in 1% PFA before acquisition on a LSR Fortessa and analysed with FlowJo10. For further phenotypic analysis of rHA^+^ IgD^-^CD10^-^ B cells, a cut-off was $\ge$5 rHA probe-specific IgD^-^CD10^-^ B cells was employed.

### Statistical analysis

No statistical methods were used to predetermine sample sizes, but our sample sizes mirror those previously published^17^. Normality tests were not performed and nonparametric statistical analyses were performed in the study. Statistical significance was assessed using a two-sided Mann–Whitney U-test, two-sided Wilcoxon signed-rank test, two-way ANOVA and Spearman correlation coefficient (r_s_) in GraphPad Prism V10.4.1 unless stated otherwise. Correlation matrices (Fig. 2e; Fig. 7a, b; and Supplementary Fig. 9) were generated in R using corrplot v.0.92 (https://github.com/taiyun/corrplot).

### Reporting summary

Further information on research design is available in the Nature Portfolio Reporting Summary linked to this article.

## Supplementary information

Supplementary Infirmation

Peer Review file

Description of additional supplementary Files

Supplementary Data 1

Reporting Summary

## Source data

Source Data

## Data Availability

Source data are provided with this paper. All other data are provided in the article and its Supplementary files or from the corresponding author upon reasonable request.
