# Supplementary material for: Atypical B cells and inflammatory profiles delineate immunity to influenza vaccination in First Nations and non-Indigenous people with chronic multimorbidity
Source: Nat Commun. 2026 Jun 5;17:7210. doi: 10.1038/s41467-026-73988-z (PMC13396489; doi:10.1038/s41467-026-73988-z)
Supplement: Supplementary file 1 — Supplementary Infirmation [file 41467_2026_73988_MOESM1_ESM.pdf]

SUPPLEMENTARY INFORMATION

Atypical B-cells and inflammation delineate influenza vaccination immunity in First Nations and non-Indigenous people with multimorbidity

Morgan J Skinner[1], Lukasz Kedzierski[1], Ruth A Purcell[1], Mark Mayo[2], Bianca F Middleton[2], Lilith F Allen[1], Ruth R Hagen[1], Alexandra Hinchcliff[2], Matilda Clark[2], Caitlin Kent[2], Malet Aban[3], Heidi Peck[3], Hayley A McQuilten[1], Arnold Reynaldi[4], Ashleigh I Holloway[1], Angelica Tan[2], Vanessa Rigas[2], Erin Gargen[2], Miles P Davenport[4], Stephen J Kent[1,5], Ian Barr[3], Hyon-Xhi Tan[1], Adam K Wheatley[1], Amy W Chung[1], Jane Nelson[2], Adrian Miller[6], Thi HO Nguyen[1], Jane Davies[2]*, Louise C Rowntree[1]* and Katherine Kedzierska[1,7]*

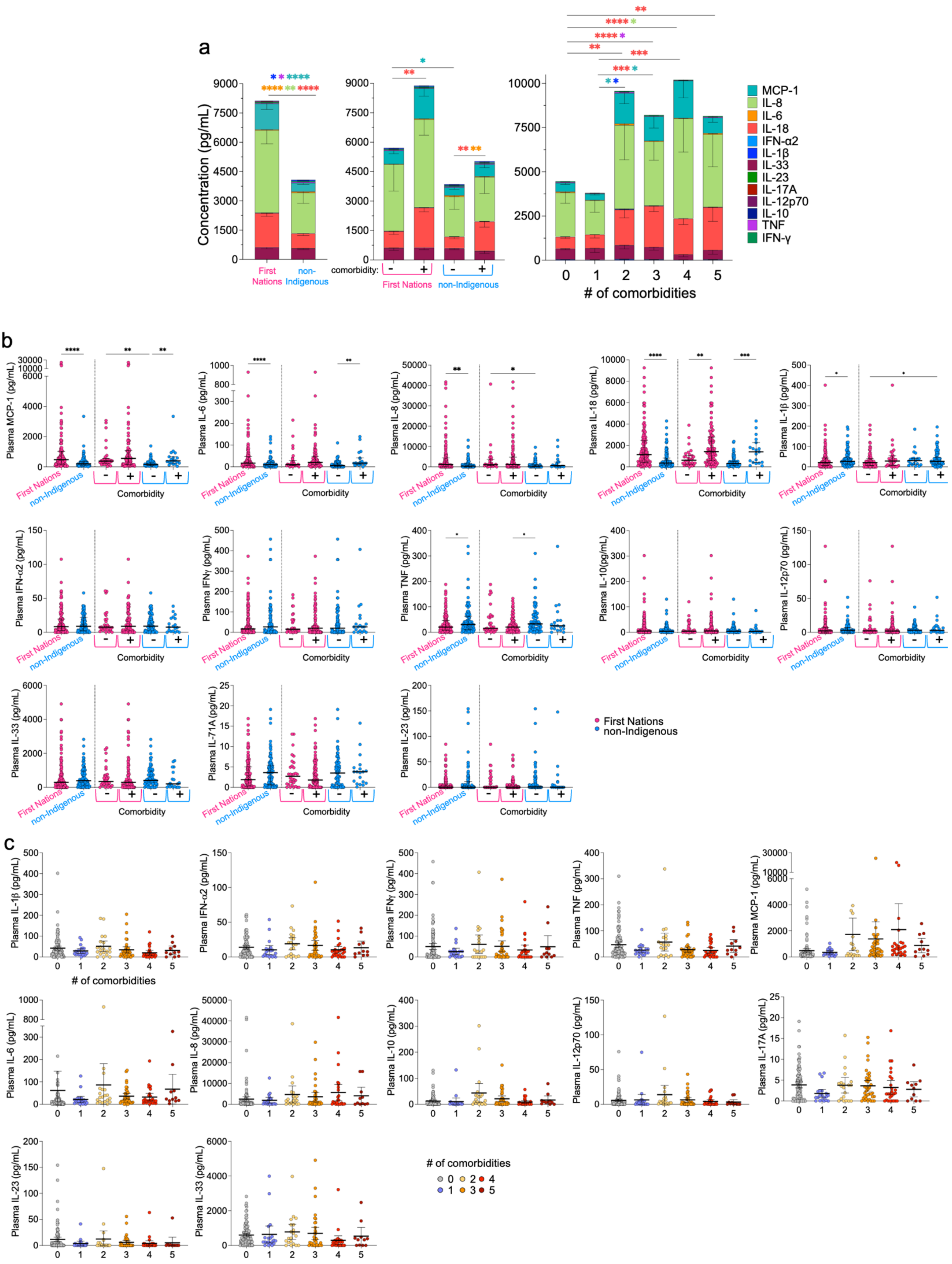
a
Concentration (pg/mL)
First Nations
non-Indigenous
comorbidity:
First Nations
non-Indigenous
# of comorbidities
MCP-1
IL-8
IL-6
IL-18
IFN-α2
IL-1β
IL-33
IL-23
IL-17A
IL-12p70
IL-10
TNF
IFN-γ
b
Plasma MCP-1 (pg/mL)
Plasma IL-6 (pg/mL)
Plasma IL-8 (pg/mL)
Plasma IL-18 (pg/mL)
Plasma IL-1β (pg/mL)
Plasma IFN-α2 (pg/mL)
Plasma IFNγ (pg/mL)
Plasma TNF (pg/mL)
Plasma IL-10(pg/mL)
Plasma IL-12p70 (pg/mL)
Plasma IL-33 (pg/mL)
Plasma IL-71A (pg/mL)
Plasma IL-23 (pg/mL)
Comorbidity
First Nations
non-Indigenous
c
# of comorbidities
Plasma IL-1β (pg/mL)
Plasma IFN-α2 (pg/mL)
Plasma IFNγ (pg/mL)
Plasma TNF (pg/mL)
Plasma MCP-1 (pg/mL)
Plasma IL-6 (pg/mL)
Plasma IL-8 (pg/mL)
Plasma IL-10 (pg/mL)
Plasma IL-12p70 (pg/mL)
Plasma IL-17A (pg/mL)
Plasma IL-23 (pg/mL)
Plasma IL-33 (pg/mL)
# of comorbidities

Supplementary Figure 1. **Baseline concentrations of 13 plasma cytokine and chemokines concentrations in First Nations and non-Indigenous participants.** Representative stacked plot depicting plasma concentrations of 13 cytokines and chemokines measured by LEGENDplex assay compared between **a** First Nations (n=125) and non-Indigenous (n=99) cohorts (left) with (n_{FN}=95, n_{NI} =19) and without (n_{FN}=30, n_{NI}=80) comorbidities (middle) and degrees of multimorbidity (n_0=110, n_1=20, n_2=20, n_3=36, n_4=26, n_5=11) (right). **b,c** Individual cytokine concentrations by ethnicity and comorbidity (**b**) and multimorbidity (**c**). Mean and SEM are shown (**a**), with statistical significance determined by two-way ANOVA with two-sided Tukey's test for multiple comparison between sample timepoints and participants. (**b,c**) Bolded line represents median and error bars depict IQR, with statistical significance determined by two-sided Mann-Whitney test for unpaired comparison between groups corrected with Dunn's test for multiple comparisons. Exact p values available in Source Data File. * p<0.05, ** p<0.01, *** p<0.001 and **** p<0.0001.

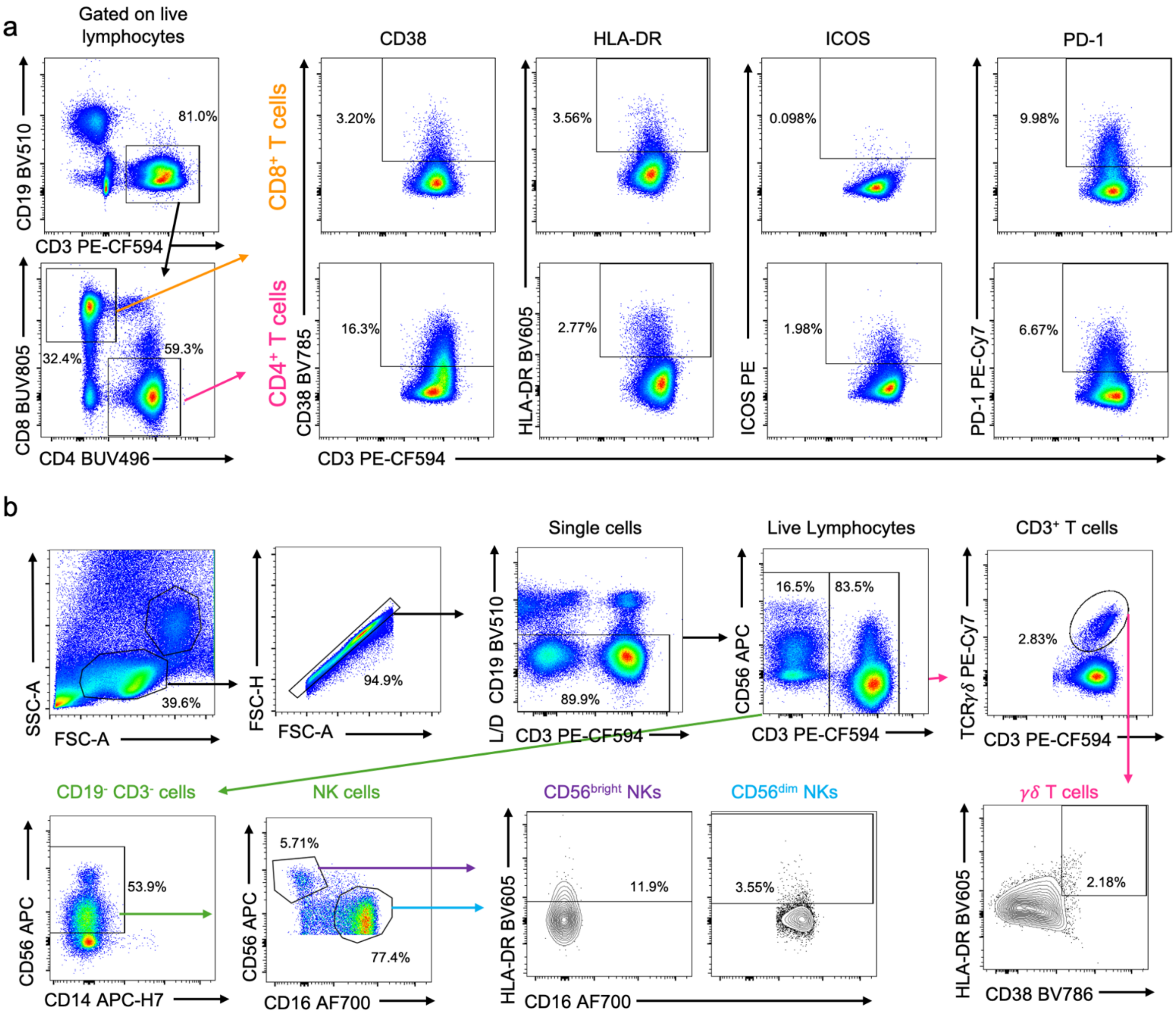

Supplementary Figure 2. **Gating strategy of baseline frequencies of activated innate and adaptive immune cells and comparison between specific comorbidity presentations. a** Representative flow cytometry plots of CD4^{+} and CD8^{+} T cell activation markers (CD38, HLA-DR, ICOS and PD-1) used in Boolean gating strategy. **b** Representative flow cytometry plots of Natural Killer (NK) cell subset (CD56/CD16) and activation (HLA-DR), and γδ T cell hyperactivation (HLA-DR^{+} CD38^{+}) gating.

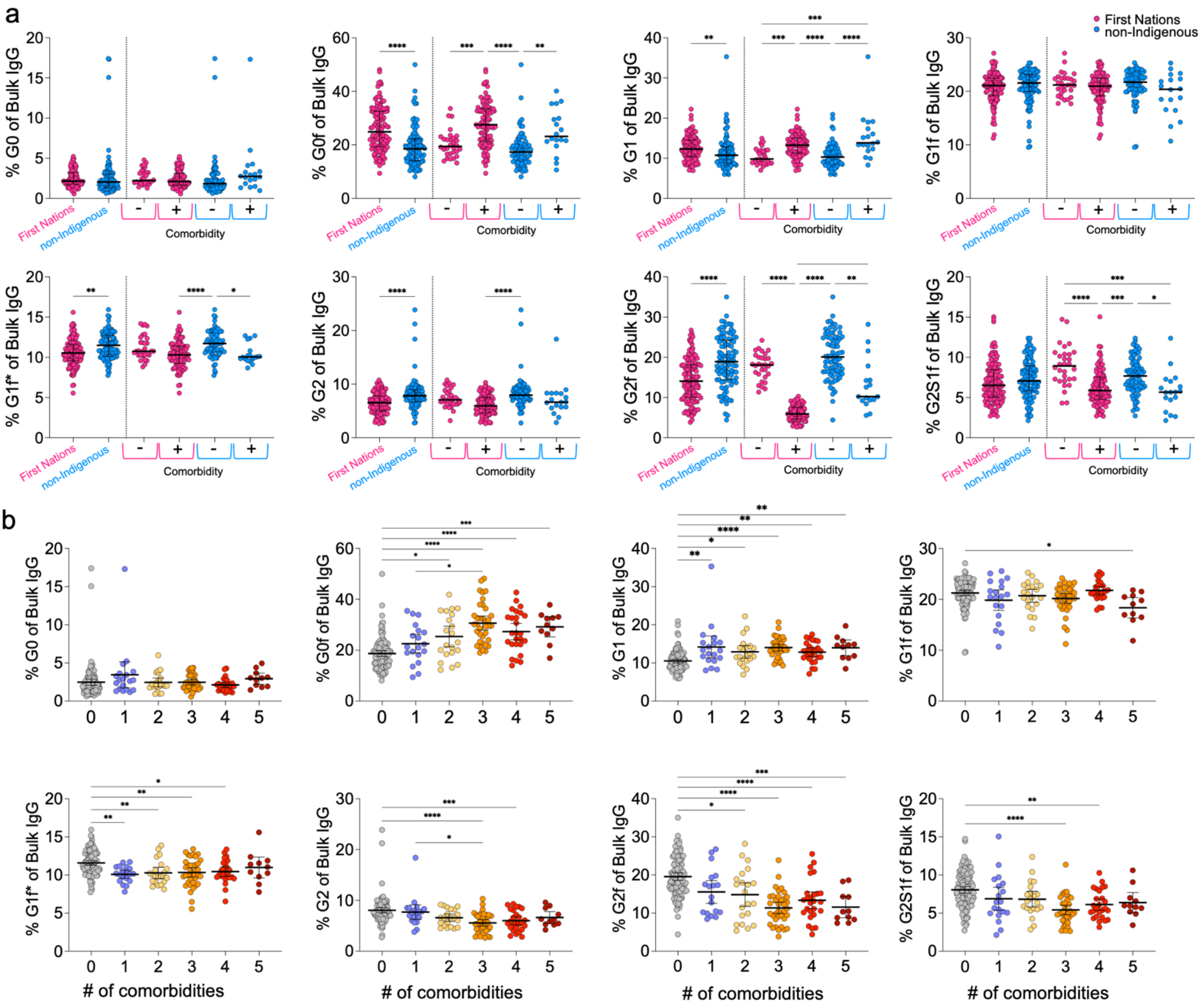

Supplementary Figure 3. **Bulk IgG glycosylation. a,b** Relative abundances of Bulk IgG N-linked glycoforms of First Nations (n=125) and non-Indigenous (n=97) cohorts with (n_{FN}=95, n_{NI}=18) and without (n_{FN}=30, n_{NI}=79) comorbidities (**a**) and number of multimorbidity (n_0=109, n_1=19, n_2=21 n_3=36, n_4=26, n_5=11) (**b**). Bolded line represents the median and error bars depict IQR (**a, b**), with statistical significance of individual dot plots determined using two-sided Mann-Whitney test for unpaired comparison between groups corrected with Dunn's test for multiple comparisons. Exact p values available in Source Data File. * p<0.05, ** p<0.01, *** p<0.001 and **** p<0.0001.

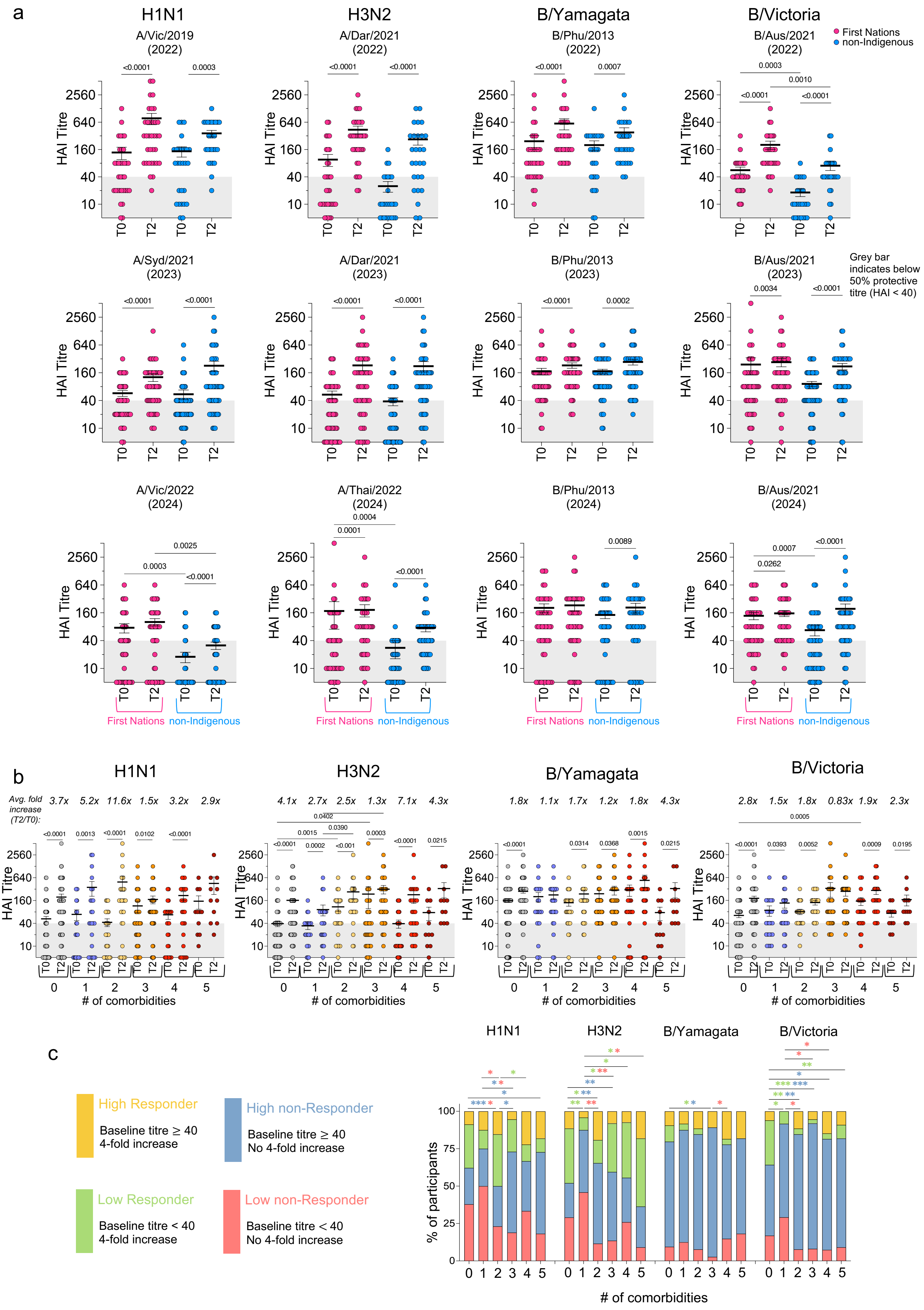
a
H1N1
H3N2
B/Yamagata
B/Victoria
First Nations
non-Indigenous
A/Vic/2019 (2022)
A/Dar/2021 (2022)
B/Phu/2013 (2022)
B/Aus/2021 (2022)
A/Syd/2021 (2023)
A/Dar/2021 (2023)
B/Phu/2013 (2023)
B/Aus/2021 (2023)
Grey bar indicates below 50% protective titre (HAI < 40)
A/Vic/2022 (2024)
A/Thai/2022 (2024)
B/Phu/2013 (2024)
B/Aus/2021 (2024)
HAI Titre
T0
T2
b
Avg. fold increase (T2/T0):
H1N1: 3.7x 5.2x 11.6x 1.5x 3.2x 2.9x
H3N2: 4.1x 2.7x 2.5x 1.3x 7.1x 4.3x
B/Yamagata: 1.8x 1.1x 1.7x 1.2x 1.8x 4.3x
B/Victoria: 2.8x 1.5x 1.8x 0.83x 1.9x 2.3x
# of comorbidities
c
High Responder
Baseline titre ≥ 40
4-fold increase
High non-Responder
Baseline titre ≥ 40
No 4-fold increase
Low Responder
Baseline titre < 40
4-fold increase
Low non-Responder
Baseline titre < 40
No 4-fold increase
% of participants
# of comorbidities

Supplementary Figure 4. **Serum HAI titres toward 2022-2024 quadrivalent vaccine components among First Nations and non-Indigenous participants. a,b** Paired haemagglutinin inhibitory serum antibodies determined by HAI assay prior to vaccination (T0) and post-vaccination (T2) against H1, H3, B/Yamagata and B/Victoria vaccine components (**a**) respective to each Southern Hemisphere influenza season/vaccination year (2022; n_{FN}=34 n_{NI}=26) (2023; n_{FN}=54 n_{NI}=56) (2024; n_{FN}=49 n_{NI}=54) and (**b**) according to number of comorbidities (n_0=148, n_1=24, n_2=26 n_3=37, n_4=27, n_5=11). **c** Definitions of responder status by baseline HAI titre and post-vaccination antibody titre fold change (left) and frequency of vaccine strain-specific responder status groups (right) according to number of comorbidities (n_0=148, n_1=24, n_2=26 n_3=37, n_4=27, n_5=11). Bold line (**a, b**) indicates mean, and error bars indicate SEM. Data points above grey bar indicates titre of ≥ 40 which confers 50% seroprotection. Statistical significance (**a, b**) was determined by Wilcoxon ranked sum test for paired pre- and post-vaccination titres within groups, and two-sided Mann-Whitney test for unpaired comparison between groups corrected with Dunn's test for multiple comparisons. Statistical significance of responder status group frequency (**c**) was determined by Chi square test and/or Fisher's exact test where any cell value <5. Exact p values (**c**) available in Source Data File. * p<0.05, ** p<0.01, *** p<0.001 and **** p<0.0001.

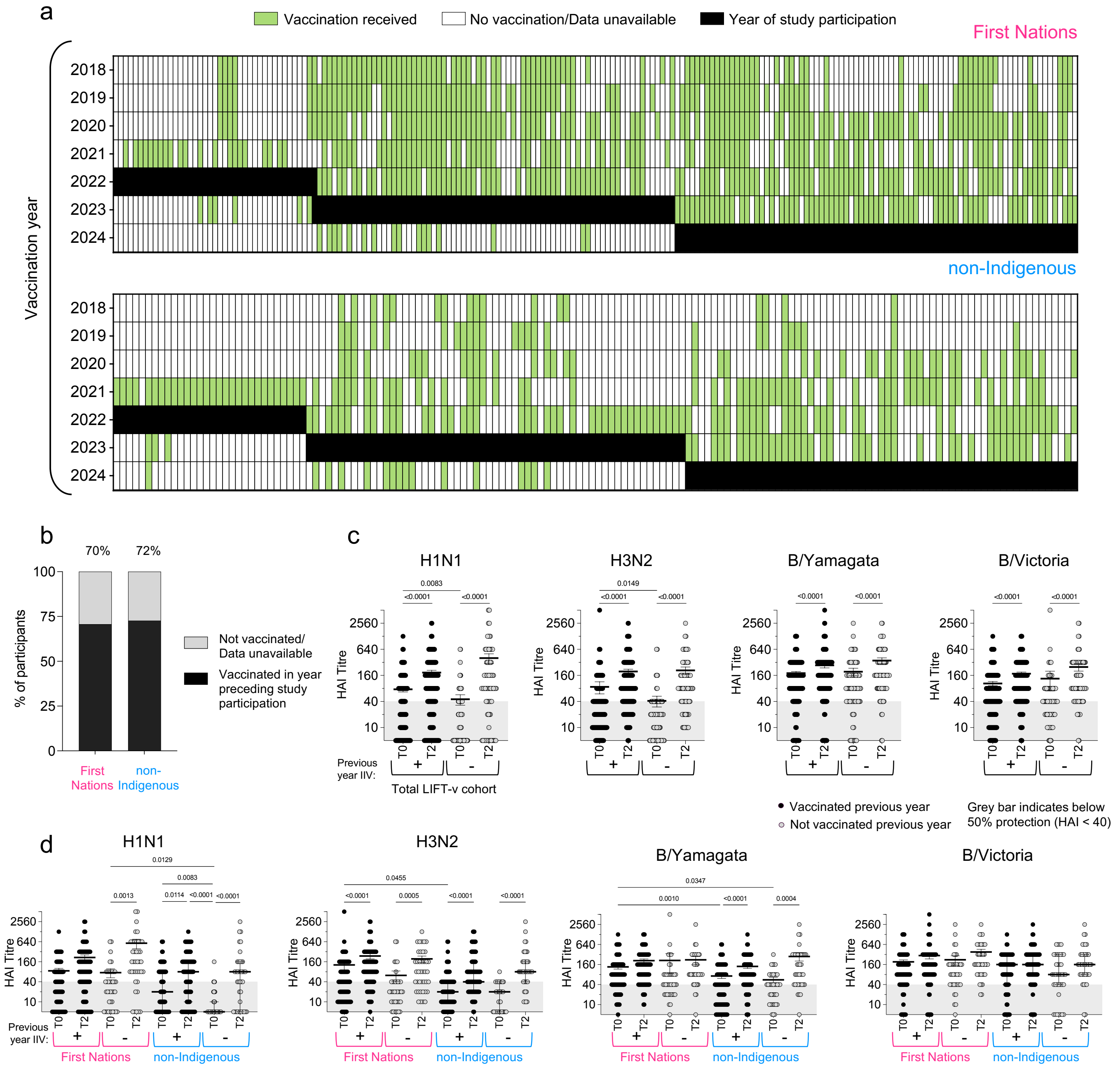

Supplementary Figure 5. **Vaccination history of study participants had limited impact on serum HAI titres toward 2022-2024 quadrivalent vaccine components among First Nations and non-Indigenous participants. a** Categorical heat maps where each column represents a single participant, depicting known influenza vaccination history between 2018-2024 for First Nations (top) and non-Indigenous (bottom) cohorts. Confirmed vaccination (green), no vaccination/data unavailable (white) and year of study participation (black) are shown for each donor. **b** Frequencies of First Nations (left, pink)(n=137) and non-Indigenous (n=136) participants (right, blue) who received an influenza virus vaccination in the year previous to their participation in our study. **c,d** Hemagglutinin inhibitory serum antibodies determined by HAI assay prior to vaccination (T0) and post-vaccination (T2) against H1, H3, B/Yamagata and B/Victoria vaccine components grouped by participants previous years vaccination status for (**c**) the total LIFT-v cohort (n_{Vax}=194, n_{NoVax}=79) and d) First Nations (n_{Vax}=97, n_{NoVax}=40) and non-Indigenous (n_{Vax}=97, n_{NoVax}=39) cohorts. Bold line (**c,d**) indicates mean, and error bars indicate SEM. Data points above grey bar indicates titre of ≥ 40 which confers 50% protection. Statistical significance (**c,d**) was determined by Wilcoxon ranked sum test for paired pre- and post-

vaccination titres within groups, and two-sided Mann-Whitney test for unpaired comparison between groups with Dunn's multiple comparisons test. * p<0.05, ** p<0.01, *** p<0.001 and **** p<0.0001.

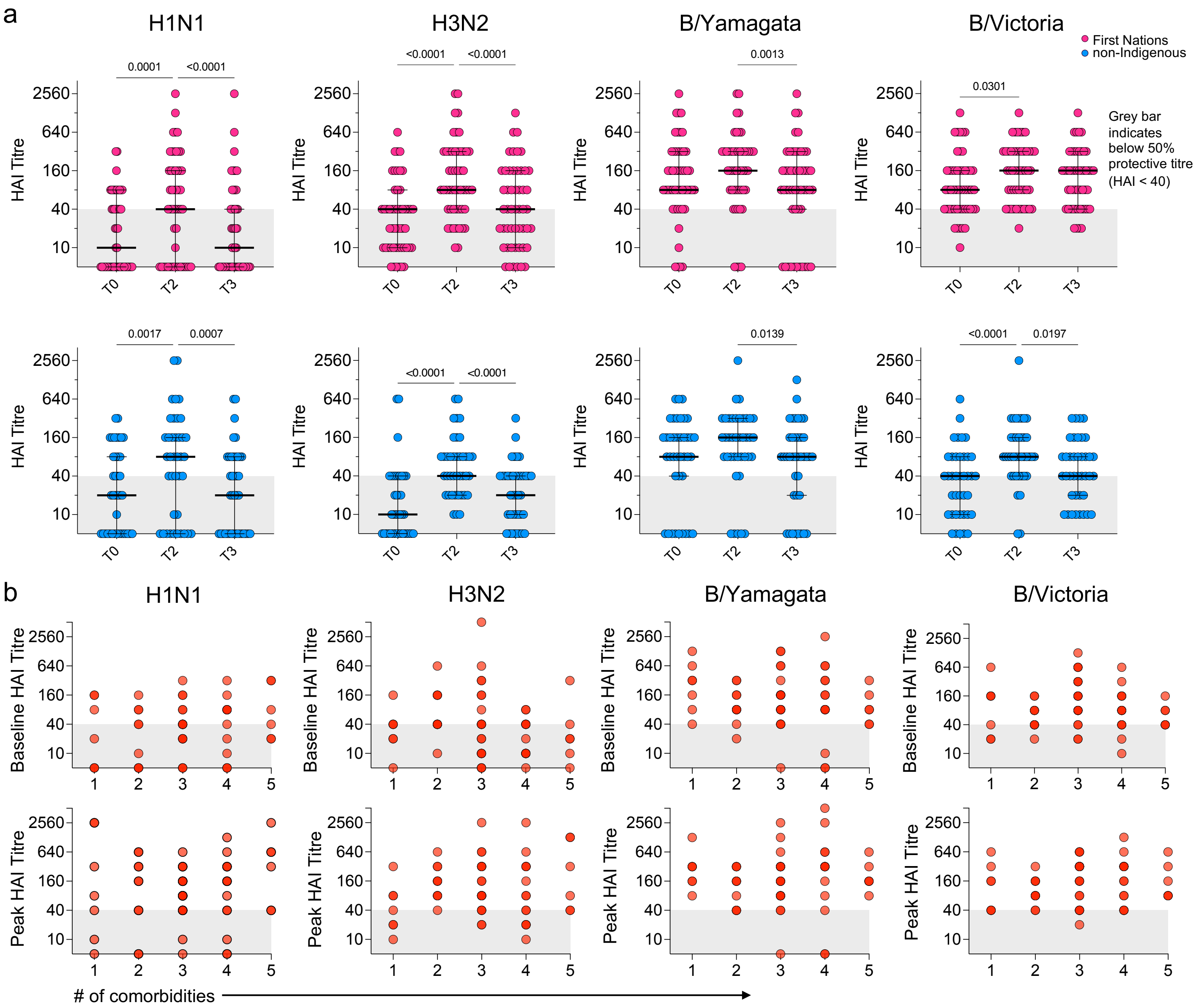

Supplementary Figure 6. **Serum HAI kinetics is not impacted by multimorbidity. a** Haemagglutinin inhibiting serum antibodies determined by HAI assay at T0, T2 and T3 against H1, H3, B/Yamagata and B/Victoria vaccine components (n_{FN}=51, n_{NI}=39). **b** Comparison of base and peak HAI titres among longitudinal cohort with increasing number of comorbidities (n=57). Data points above grey bar indicates titre of ≥ 40 which confers 50% seroprotection. Statistical significance (**a**) was determined by Wilcoxon ranked sum test for paired pre- and post-vaccination titres within groups, and two-sided Mann-Whitney test for unpaired comparison between groups corrected with Dunn's test for multiple comparisons. * p<0.05, ** p<0.01, *** p<0.001 and **** p<0.0001.

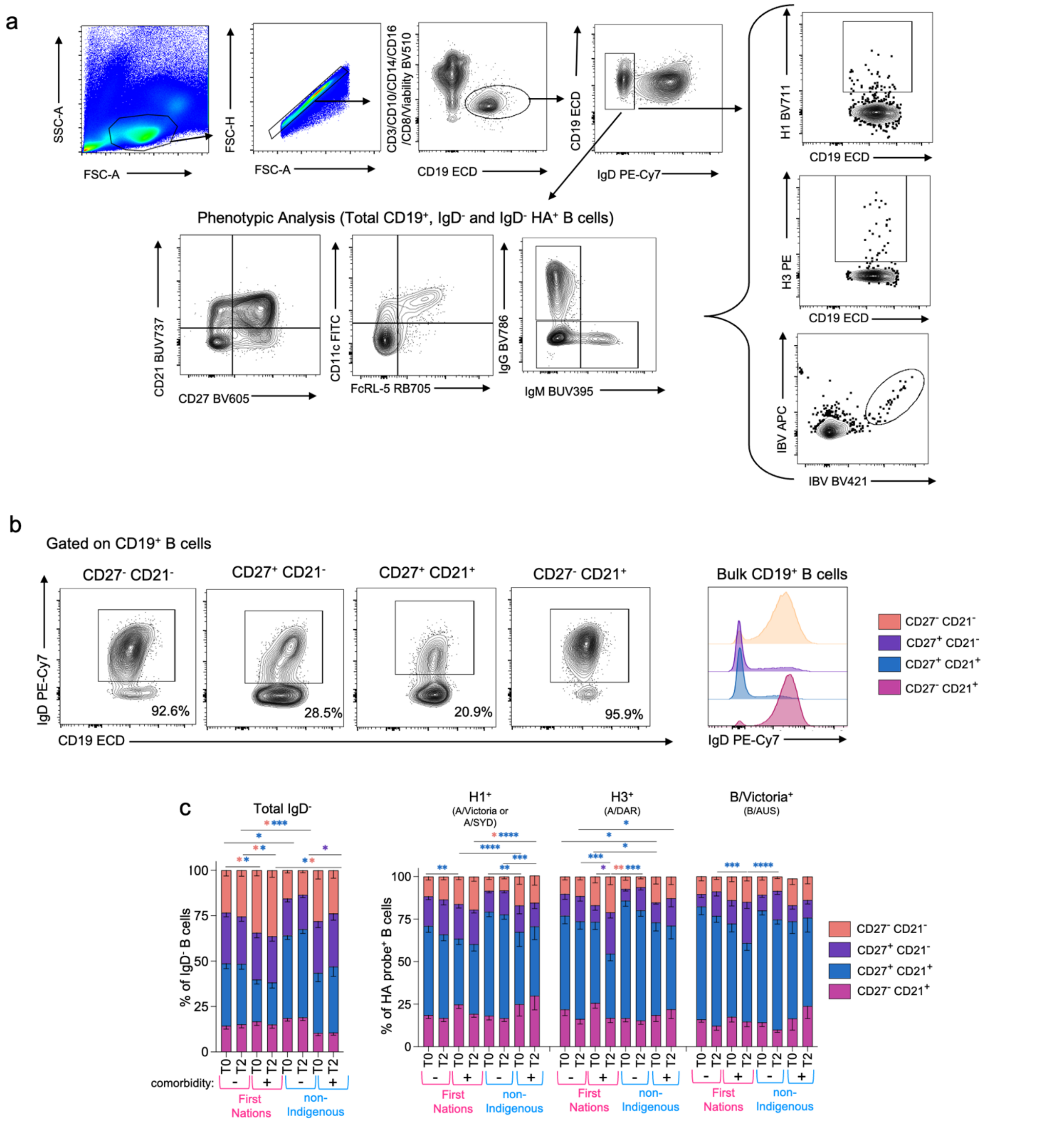

Supplementary Figure 7. **rHA probe panel gating strategy and of rHA-specific IgD⁻ CD10⁻ B cells phenotypes in First Nations and non-Indigenous with and without comorbidities. a** Representative flow cytometry plots of rHA$^+$ B cell antibody panel gating strategy. **b** Representative flow cytometry plots and histogram of IgD expression of CD21/CD27 phenotypic bulk CD19$^+$ B cells populations. **c** Proportions of CD21/CD27 phenotypic populations of total IgD$^-$ and HA-specific B cells in First Nations and non-Indigenous participants with (n_{FN}=30, n_{NI}=12) and without (n_{FN}=22, n_{NI}=34) comorbidities. Statistical significance of phenotypic B cell population frequencies between sample timepoints and participant groups (**c**) was calculated by by two-way ANOVA with two-sided Tukey's test for multiple comparisons. Exact p values (**c**) available in Source Data File. * p<0.05, ** p<0.01, *** p<0.001 and **** p<0.0001.

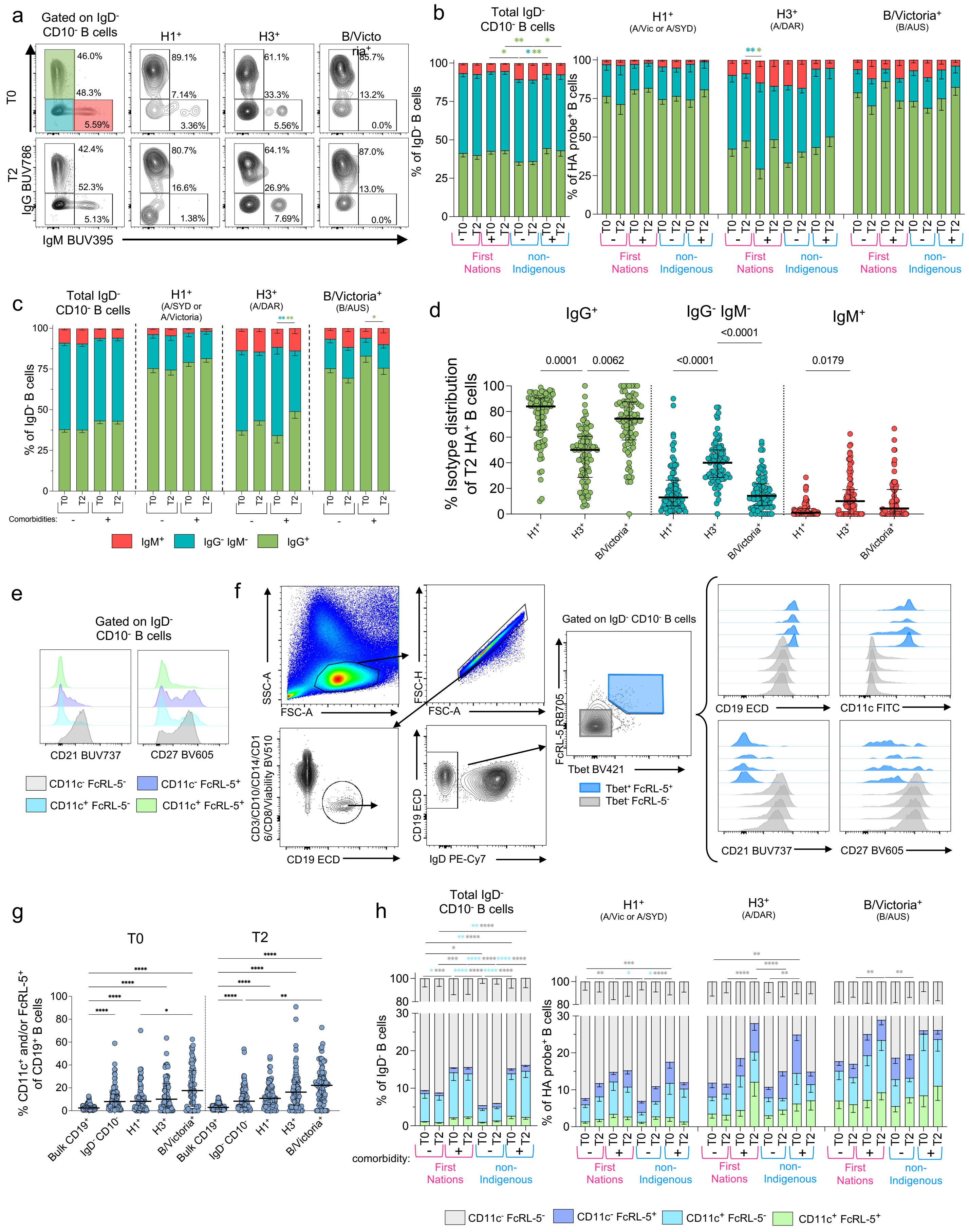
a
Gated on IgD- CD10- B cells
H1+
H3+
B/Victoria+
T0
T2
IgG BUV786
IgM BUV395
b
Total IgD- CD10- B cells
H1+ (A/Vic or A/SYD)
H3+ (A/DAR)
B/Victoria+ (B/AUS)
% of IgD- B cells
% of HA probe+ B cells
First Nations
non-Indigenous
c
Total IgD- CD10- B cells
H1+ (A/SYD or A/Victoria)
H3+ (A/DAR)
B/Victoria+ (B/AUS)
% of IgD- B cells
Comorbidities:
IgM+
IgG- IgM-
IgG+
d
IgG+
IgG- IgM-
IgM+
% Isotype distribution of T2 HA+ B cells
0.0001
0.0062
<0.0001
<0.0001
0.0179
e
Gated on IgD- CD10- B cells
CD21 BUV737
CD27 BV605
CD11c- FcRL-5-
CD11c- FcRL-5+
CD11c+ FcRL-5-
CD11c+ FcRL-5+
f
SSC-A
FSC-A
FSC-H
CD3/CD10/CD14/CD16/CD8/Viability BV510
CD19 ECD
IgD PE-Cy7
Gated on IgD- CD10- B cells
FcRL-5 RB705
Tbet BV421
Tbet+ FcRL-5+
Tbet- FcRL-5-
CD11c FITC
g
T0
T2
% CD11c+ and/or FcRL-5+ of CD19+ B cells
Bulk CD19+
IgD- CD10-
H1+
H3+
B/Victoria+
h
Total IgD- CD10- B cells
H1+ (A/Vic or A/SYD)
H3+ (A/DAR)
B/Victoria+ (B/AUS)
% of IgD- B cells
% of HA probe+ B cells
comorbidity:
First Nations
non-Indigenous
CD11c- FcRL-5-
CD11c- FcRL-5+
CD11c+ FcRL-5-
CD11c+ FcRL-5+

Supplementary Figure 8. **Atypical B cell marker expression and isotype distribution of total IgD$^-$ CD10$^-$ and HA-specific IgD$^-$ CD10$^-$ B cells in First Nations and non-Indigenous individuals with and without comorbidities.**

a,b Representative flow cytometry plots (**a**) and proportions (**b**) of immunoglobulin (IgG and IgM) isotype distribution of total IgD$^-$ and HA-specific B cells in First Nations and non-Indigenous participants with (n_{FN}=30, n_{NI}=12) and without (n_{FN}=22, n_{NI}=34) comorbidities. **c** Proportions of immunoglobulin (IgG and IgM) isotype distribution of total IgD$^-$ and rHA-specific B cells in participants with (n=42) and without (n=56) comorbidities irrespective of ethnicity. **d** Comparison of T2 rHA-specific IgD$^-$ CD10$^-$ B cell Ig isotype frequencies. **e** Representative histogram depicting CD21/CD27 phenotype of atBC marker expressing cell subsets. **f** Representative flow cytometry plots demonstrating Tbet, FcRL-5 and CD11c co-expression in IgD$^-$ CD10$^-$ B cells on a subset of vaccinated donors (n=4). **g** Proportion of CD11c$^+$ and/or FcRL-5$^+$ B cells at T0 and T2 between CD19$^+$, IgD$^-$ CD19$^+$ and rHA-specific IgD$^-$ CD19$^+$ B cells (n=98). **h** Proportions of atypical B cell markers (FcRL-5 and CD11c) expression on total IgD$^-$ and HA-specific B cells in First Nations and non-Indigenous participants with (n_{FN}=30, n_{NI}=12) and without (n_{FN}=22, n_{NI}=34) comorbidities. Statistical significance of phenotypic B cell population frequencies between sample timepoints and participant groups (**b,c,h**) was calculated by by two-way ANOVA with two-sided Tukey's test for multiple comparisons. Statistical significance (**d,g**) determined using two-sided Mann-Whitney test corrected with Dunn's test for multiple comparisons. Unpaired comparison between groups. Exact p values (**b,c,h**) available in Source Data File. * p<0.05, ** p<0.01, *** p<0.001 and **** p<0.0001.

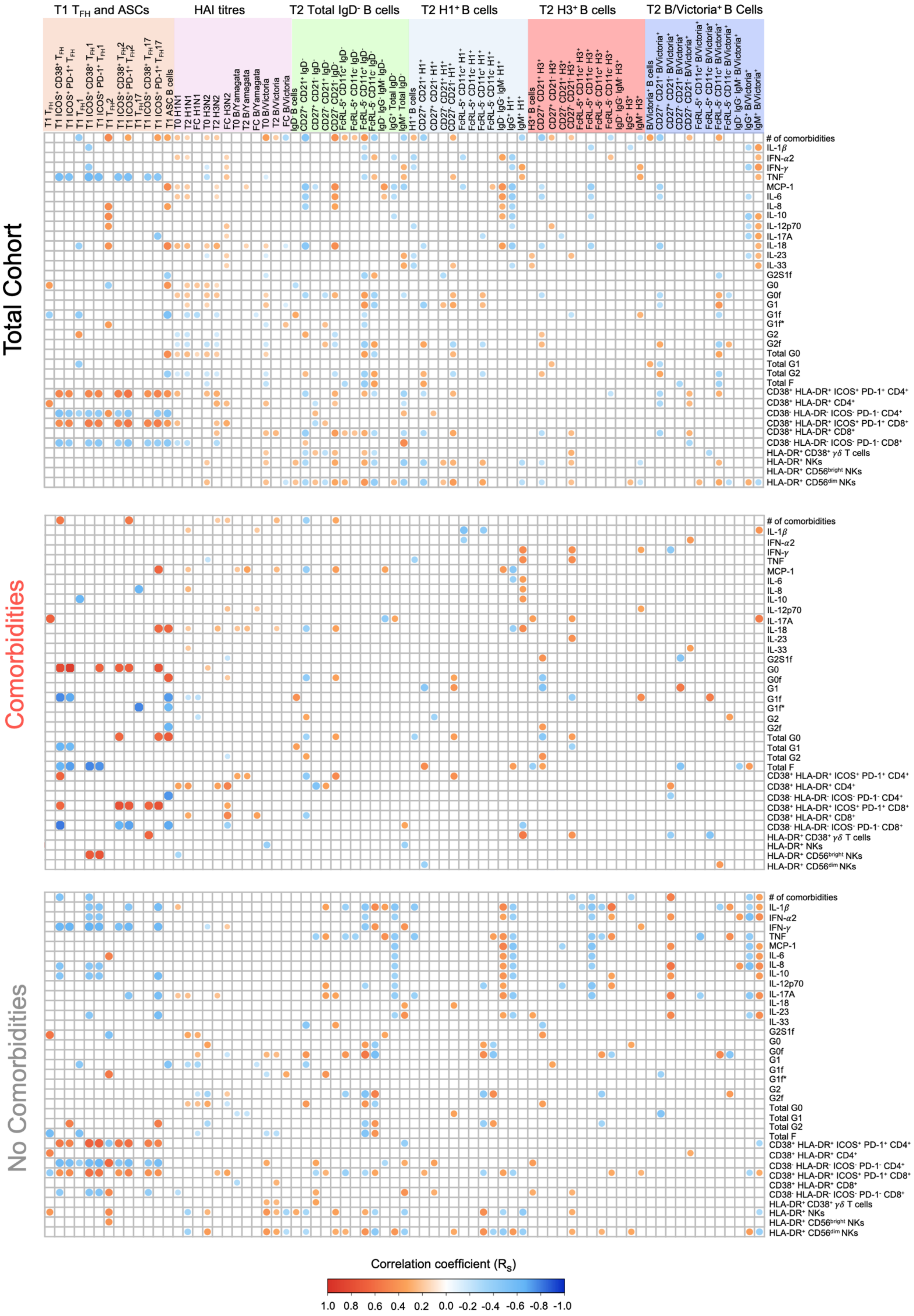

T1 TFH and ASCs
HAI titres
T2 Total IgD- B cells
T2 H1+ B cells
T2 H3+ B cells
T2 B/Victoria+ B Cells
T1 TFH
T1 ICOS+ CD38+ TFH
T1 ICOS+ PD-1+ TFH
T1 TFH1
T1 ICOS+ CD38+ TFH1
T1 ICOS+ PD-1+ TFH1
T1 TFH2
T1 ICOS+ CD38+ TFH2
T1 ICOS+ PD-1+ TFH2
T1 TFH17
T1 ICOS+ CD38+ TFH17
T1 ICOS+ PD-1+ TFH17
T1 ASC B cells
T0 H1N1
T2 H1N1
FC H1N1
T0 H3N2
T2 H3N2
FC H3N2
T0 B/Yamagata
T2 B/Yamagata
FC B/Yamagata
T0 B/Victoria
T2 B/Victoria
FC B/Victoria
IgD- B cells
CD27+ CD21+ IgD-
CD27- CD21+ IgD-
CD27+ CD21- IgD-
CD27- CD21- IgD-
FcRL-5+ CD11c+ IgD-
FcRL-5- CD11c+ IgD-
FcRL-5+ CD11c- IgD-
FcRL-5- CD11c- IgD-
IgD- IgG- IgM- IgD-
IgG+ Total IgD-
IgM+ Total IgD-
H1+ B cells
CD27+ CD21+ H1+
CD27- CD21+ H1+
CD27+ CD21- H1+
CD27- CD21- H1+
FcRL-5+ CD11c+ H1+
FcRL-5- CD11c+ H1+
FcRL-5+ CD11c- H1+
FcRL-5- CD11c- H1+
IgD- IgG- IgM- H1+
IgG+ H1+
IgM+ H1+
H3+ B cells
CD27+ CD21+ H3+
CD27- CD21+ H3+
CD27+ CD21- H3+
CD27- CD21- H3+
FcRL-5+ CD11c+ H3+
FcRL-5- CD11c+ H3+
FcRL-5+ CD11c- H3+
FcRL-5- CD11c- H3+
IgD- IgG- IgM- H3+
IgG+ H3+
IgM+ H3+
B/Victoria+ B cells
CD27+ CD21+ B/Victoria+
CD27- CD21+ B/Victoria+
CD27+ CD21- B/Victoria+
CD27- CD21- B/Victoria+
FcRL-5+ CD11c+ B/Victoria+
FcRL-5- CD11c+ B/Victoria+
FcRL-5+ CD11c- B/Victoria+
FcRL-5- CD11c- B/Victoria+
IgD- IgG- IgM- B/Victoria+
IgG+ B/Victoria+
IgM+ B/Victoria+
Total Cohort
Comorbidities
No Comorbidities
# of comorbidities
IL-1β
IFN-α2
IFN-γ
TNF
MCP-1
IL-6
IL-8
IL-10
IL-12p70
IL-17A
IL-18
IL-23
IL-33
G2S1f
G0
G0f
G1
G1f
G1f*
G2
G2f
Total G0
Total G1
Total G2
Total F
CD38+ HLA-DR+ ICOS+ PD-1+ CD4+
CD38+ HLA-DR+ CD4+
CD38- HLA-DR- ICOS- PD-1- CD4+
CD38+ HLA-DR+ ICOS+ PD-1+ CD8+
CD38+ HLA-DR+ CD8+
CD38- HLA-DR- ICOS- PD-1- CD8+
HLA-DR+ CD38+ γδ T cells
HLA-DR+ NKs
HLA-DR+ CD56bright NKs
HLA-DR+ CD56dim NKs
Correlation coefficient (Rs)
1.0 0.8 0.6 0.4 0.2 0.0 -0.2 -0.4 -0.6 -0.8 -1.0

Supplementary Figure 9. **Correlations of baseline immune features and post-vaccination immune responses according to comorbidity status.** Correlation matrix of spearman correlations between baseline inflammatory features (y axis) and influenza-specific humoral immune responses (x axis) in total cohort (top), comorbidity-affected (middle) and non-comorbidity vaccinee (bottom) subpopulations. Only statistically significant correlations (p <0.05) are displayed.

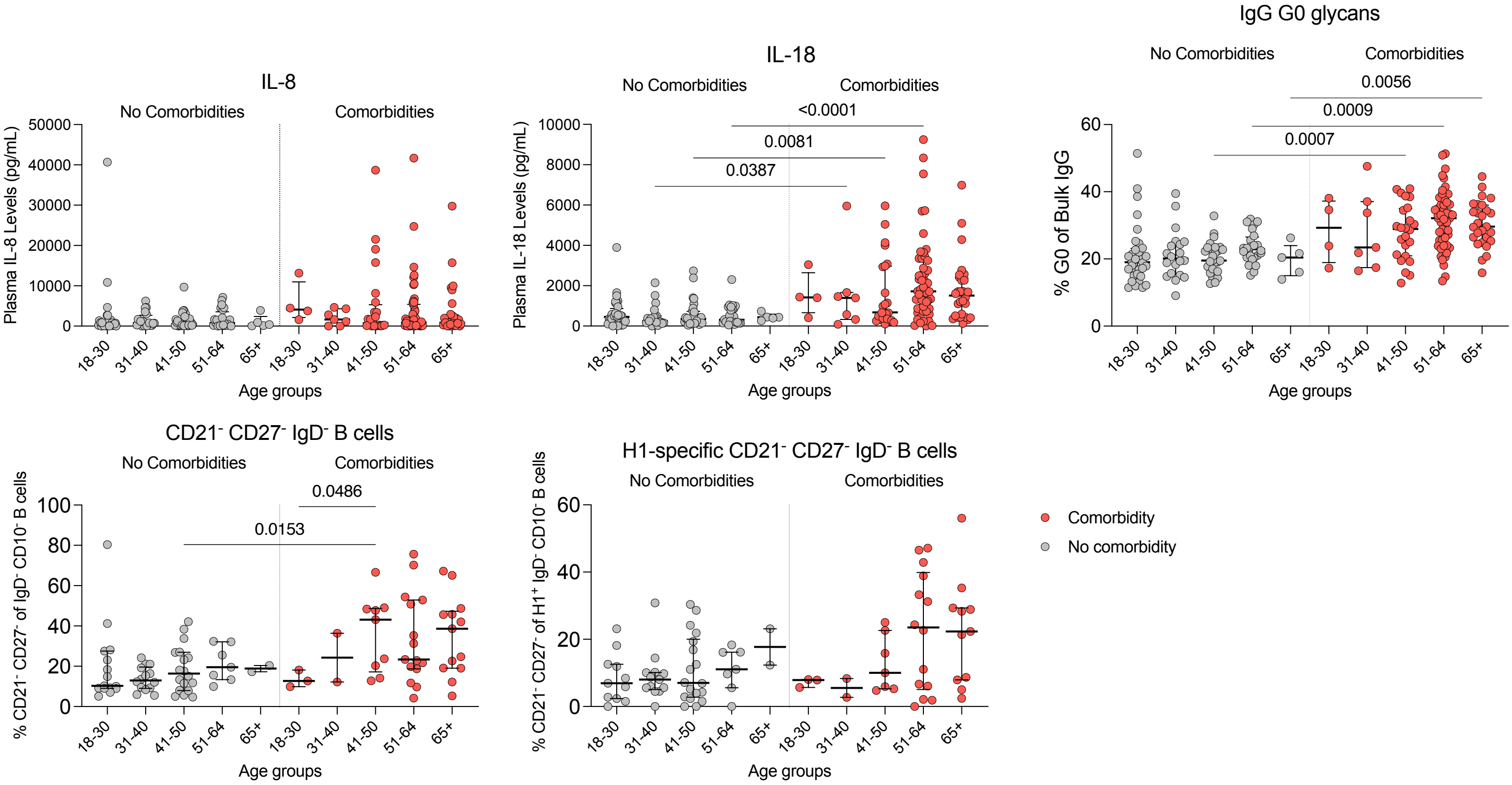

Supplementary Figure 10. **Magnitude of baseline inflammation and perturbed immunity is increased in individuals with comorbidities compared to non-comorbidity individuals of matched age.** Comparison of key baseline inflammatory (IL-8, IL-18 and IgG G0) and perturbed cellular features (atypical B cell phenotypes) among comorbidity (red) and non-comorbidity (grey) individuals between approximate age subsets. Bolded line represents the median and error bars depict IQR, with statistical significance of individual dot plots determined using two-sided Mann-Whitney test for unpaired comparison between groups corrected with Dunn's test for multiple comparisons. * p<0.05, ** p<0.01, *** p<0.001 and **** p<0.0001.

Supplementary Tables

Supplementary Table 1. **Summary of LIFT-v cohort characteristics**

	Total LIFT-v Cohort	First Nations	Non-Indigenous
	n = 344	n = 194	n = 150
**Demographic**			
Age (median, range)	49 (19-81)	53 (19-78)	41 (19-81)
Female (n, %)	209 (60%)	112 (58%)	97 (64%)
BMI (median, range)	25.5 (14.8 - 48.8)	26.0 (14.8 - 48.8)	25.0 (18.3 - 42.0)
**Ethnicity (n, %)**			
Aboriginal	182 (52.9%)	182 (93.8%)	0 (0%)
Torres Strait Islander	4 (1.2%)	4 (2.1%)	0 (0%)
Aboriginal and Torres Strait Islander	8 (2.3%)	8 (4.1%)	1 (0%)
Caucasian	128 (37.2%)	0 (0%)	128 (85.3%)
Asian	13 (3.8%)	0 (0%)	13 (8.7%)
Other	9 (2.6%)	0 (0%)	9 (6.0%)
**Regionality (n, %)**			
Remote Northen Territory	47 (13.7%)	43 (22.2%)	4 (2.7%)
Urban Darwin	261 (75.9%)	151 (77.8%)	110 (73.3%)
Melbourne	36 (10.5%)	0 (%)	36 (24.0%)
**Comorbidities (n, %)**			
Renal Disease	135 (39.2%)	119 (61.3%)	16 (10.7%)
Diabetes	100 (29.1%)	95 (49%)	5 (3.3%)
Cardiac Disease	95 (27.6%)	89 (45.9%)	6 (4.0%)
Immunosuppression	75 (21.8%)	61 (31.4%)	14 (9.3%)
Chronic Respiratory Disease	43 (12.5%)	41 (21.1%)	2 (1.3%)
Liver Disease	20 (5.8%)	19 9.8%)	1 (0.7%)
**Days post vaccination timepoints (median, range)**			
Timepoint 1 (T1)	7 (6-12)	7 (6-12)	7 (7-12)
Timepoint 2 (T2)	48 (21-121)	49 (21-121)	48 (28-110)
Timepoint 3 (T3)	224 (182-286)	212 (182-286)	225 (200-273)

Supplementary Table 2. **Serum HAI titre virus strains**

	Vaccination Year		
Vaccine Component	2022	2023	2024
A/H1N1	A/Victoria/2570/2019	A/Sydney/5/2021	A/Victoria/4897/2022
A/H3N2	A/Darwin/6/2021	A/Darwin/6/2021	A/Thailand/8/2022
B/Yamagata	B/Phuket/3073/2013	B/Phuket/3073/2013	B/Phuket/3073/2013
B/Victoria	B/Austria/1359417/2021	B/Austria/1359417/2021	B/Austria/1359417/2021

Supplementary Table 3. **T_{FH} and ASC panel**

mAb	Fluorochrome	Cat. #	Supplier
CD4	BUV496	612936	BD Horizon
CD21	BUV737	612788	BD Horizon
CD8A	BUV805	564912	BD Biosciences
CXCR5	BV421	562747	BD Horizon
CD19	BV510	562947	BD Horizon
HLA-DR	BV605	307640	BioLegend
CCR6	BV650	563922	BD Horizon
CD20	BV711	563126	BD Horizon
CD38	BV785	563964	BD Horizon
CXCR3	APC	550967	BD Biosciences
CD27	AF700	560611	BD Biosciences
L/D NIR	APC-H7	L34992	Thermofisher
CD11c	FITC	337214	BioLegend
CD45	PerCP-Cy5.5	340953	BD Biosciences
ICOS	PE	557802	BD Pharmigen
CD3	PE-CF594	562280	BD Biosciences
PD-1	PE-Cy	561272	BD Biosciences

Supplementary Table 4. **Immunophenotype panel**

mAb	Fluorochrome	Cat. #	Supplier
CD71	BV421	562995	BD Horizon
CD19	BV510	562947	BD Horizon
L/D Aqua	BV510	L34966	Thermofisher
HLA-DR	BV605	307640	BioLegend
CD4	BV650	563875	BD Horizon
CD27	BV711	563167	BD Horizon
CD38	BV785	563964	BD Horizon
CD56	APC	304610	BioLegend
CD16	AF700	302026	BioLegend
CD14	APC-H7	560180	BD Biosciences
CD45RA	FITC	555488	BD Pharmigen
CD8	PerCP-Cy5.5	340953	BioLegend
IL-18RA	PE	313808	BioLegend
CD3	PE-CF594	562280	BD Biosciences
TCRyD	PE-Cy	561272	BD Biosciences

Supplementary Table 5. **Influenza-specific B cell panel**

mAb	Fluorochrome	Cat. #	Supplier
FcRL-5	RB705	757499	BD Biosciences
CD11c	FITC	337214	BioLegend
IBV rHA Probe	Strep-APC	554067	BD Pharmigen
IgM	BUV395	563903	BD Horizon
CD21	BUV737	612788	BD Horizon
IBV rHA Probe	Strep-BV421	405225	BioLegend
Free SA		563261	BD Horizon
CD3		317332	BioLegend
CD8		301047	BioLegend
CD10	BV510	312219	BioLegend
CD14		301841	BioLegend
CD16		302047	BioLegend
L/D Aqua		L34966	Thermofisher
CD27	BV605	302830	BioLegend
CD71	BV650	334116	BioLegend
H1 rHA Probe	Strep-BV711	405241	BioLegend
IgG	BV786	564230	BD Horizon
H3 rHA Probe	Strep-PE	554061	BD Pharmigen
CD19	ECD	IM2708U	Beckman Coulter
IgD	PE-Cy7	561314	BD Biosciences
