# Supplementary material for: Atypical B cells and inflammatory profiles delineate immunity to influenza vaccination in First Nations and non-Indigenous people with chronic multimorbidity
Source: Nat Commun. 2026 Jun 5;17:7210. doi: 10.1038/s41467-026-73988-z (PMC13396489; doi:10.1038/s41467-026-73988-z)
Supplement: Supplementary file 3 — Description of additional supplementary Files [file 41467_2026_73988_MOESM3_ESM.pdf]

Description of Additional Supplementary Files

File Name: Supplementary Data 1
Description: Table containing de-identified demographic information of study participants.
