# Supplementary material for: Atypical B cells and inflammatory profiles delineate immunity to influenza vaccination in First Nations and non-Indigenous people with chronic multimorbidity
Source: Nat Commun. 2026 Jun 5;17:7210. doi: 10.1038/s41467-026-73988-z (PMC13396489; doi:10.1038/s41467-026-73988-z)
Supplement: Supplementary file 5 — Reporting Summary [file 41467_2026_73988_MOESM5_ESM.pdf]

nature portfolio

Corresponding author(s): Katherine Kedzierska

Last updated by author(s): 2026/05/01

Reporting Summary

Nature Portfolio wishes to improve the reproducibility of the work that we publish. This form provides structure for consistency and transparency in reporting. For further information on Nature Portfolio policies, see our Editorial Policies and the Editorial Policy Checklist.

Statistics

For all statistical analyses, confirm that the following items are present in the figure legend, table legend, main text, or Methods section.

n/a	Confirmed	
☐	☒	The exact sample size (*n*) for each experimental group/condition, given as a discrete number and unit of measurement
☐	☒	A statement on whether measurements were taken from distinct samples or whether the same sample was measured repeatedly
☐	☒	The statistical test(s) used AND whether they are one- or two-sided<br>*Only common tests should be described solely by name; describe more complex techniques in the Methods section.*
☐	☒	A description of all covariates tested
☐	☒	A description of any assumptions or corrections, such as tests of normality and adjustment for multiple comparisons
☐	☒	A full description of the statistical parameters including central tendency (e.g. means) or other basic estimates (e.g. regression coefficient) AND variation (e.g. standard deviation) or associated estimates of uncertainty (e.g. confidence intervals)
☐	☒	For null hypothesis testing, the test statistic (e.g. *F*, *t*, *r*) with confidence intervals, effect sizes, degrees of freedom and *P* value noted<br>*Give P values as exact values whenever suitable.*
☒	☐	For Bayesian analysis, information on the choice of priors and Markov chain Monte Carlo settings
☒	☐	For hierarchical and complex designs, identification of the appropriate level for tests and full reporting of outcomes
☐	☒	Estimates of effect sizes (e.g. Cohen's *d*, Pearson's *r*), indicating how they were calculated

Our web collection on statistics for biologists contains articles on many of the points above.

Software and code

Policy information about availability of computer code

Data collection	BD FACS DIVA v8.0.1; Thermo Ascent Software for Multiskan v2.4; LabChip GXII Touch HT Microchip-CE platform
Data analysis	FlowJo v10; Prism v9; R v4.2.0, EnhancedVolcano v1.14.0; online QOGNIT LEGENDplex™ program; LabChip GX Touch software v1.9.1010.0; LabChip GX Reviewer software v5.4.2222.0

For manuscripts utilizing custom algorithms or software that are central to the research but not yet described in published literature, software must be made available to editors and reviewers. We strongly encourage code deposition in a community repository (e.g. GitHub). See the Nature Portfolio guidelines for submitting code & software for further information.

Data

Policy information about availability of data

All manuscripts must include a data availability statement. This statement should provide the following information, where applicable:

- Accession codes, unique identifiers, or web links for publicly available datasets
- A description of any restrictions on data availability
- For clinical datasets or third party data, please ensure that the statement adheres to our policy

The published article includes all datasets generated or analyzed during the study. Source data are provided with this paper as Source Date files. FACS-source files are available from the authors upon request. The majority data of the current study are based on flow cytometry and there are hundreds of FACS-source files with a big file size. We are happy to provide any single file upon request.

Research involving human participants, their data, or biological material

Policy information about studies with human participants or human data. See also policy information about sex, gender (identity/presentation), and sexual orientation and race, ethnicity and racism.

Reporting on sex and gender	Sex of participants was assigned based on self-reporting. We reported sex-distribution between our main study populations, which was matched, however we did not recruit/design the study to achieve this. We have provided disaggregated data on participant sex in the Supplementary Information file.
Reporting on race, ethnicity, or other socially relevant groupings	Ethnicity of our participants, which included Aboriginal, Torres Strait Islander, Aboriginal & Torres Strait Islander, Asian and Caucasian people were self reported by participants. Segregation of participants based on ethnicity and comorbidity status formed the basis of our analysis and therefore was not adjusted for.
Population characteristics	Age and comorbidity status are the major co-variant population characteristics - reported in Supplementary Data 1.
Recruitment	Samples were recruited through the Menzies School of Health research in Darwin, Northern Territory and University of Melbourne, Australia. Participants were recruited from a wide range of settings including but not limited to: healthcare and university staff vaccination programs, land council community events, remote community vaccination days, hospital and health services campus including a community based dialysis unit. Overall presence of co-morbidities was consistent with reported rates for First Nations people in the Northern Territory. Signed informed consents were obtained from all blood donors prior to the study.
Ethics oversight	Experiments conformed to the principles of the Declaration of Helsinki (2013) and the Australian National Health and Medical Research Council Code of Practice. Written informed consent was obtained from all blood donors before the study. The study was approved by the Human Research Ethics Committee of the Northern Territory Department of Health and the Menzies School of Health Research (no. 2021-3964) and the University of Melbourne Human Research Ethics Committees (nos. 21864, 31236).

Note that full information on the approval of the study protocol must also be provided in the manuscript.

Field-specific reporting

Please select the one below that is the best fit for your research. If you are not sure, read the appropriate sections before making your selection.

☒ Life sciences ☐ Behavioural & social sciences ☐ Ecological, evolutionary & environmental sciences

For a reference copy of the document with all sections, see nature.com/documents/nr-reporting-summary-flat.pdf

Life sciences study design

All studies must disclose on these points even when the disclosure is negative.

Sample size	The sample size was determined by the availability of samples from Australian First Nations and non-Indigenous participants that received quadrivalent seasonal inactivated influenza vaccination in Northern Territory, Australia in 2022-2024. Additional non-Indigenous participants were also recruited through the University of Melbourne, Australia.
Data exclusions	No data were excluded with the following exception which was pre-established: donors who had a total number of less than 5 counted rHA probe-specific IgD- CD10-CD19+ B cells were excluded for further phenotypic analyses as cell numbers were too low (Fig 5f-i). This was indicated in the manuscript in Results, methods and figure legends. Information on impact of this exclusion criteria on sample retention for each probe-specificities and timepoint are indicated in the Figure 5 legend and resutls. A source data file was generated to show all data points.
Replication	Experiments could not be replicated due to limited PBMC numbers. These are rare and unique patient samples and so we were limited to performing all the available assays. To ensure reliability, all timepoints from the same patient were carried out in the same experiment.
Randomization	Randomization was not applicable to the study, participants received quadrivalent seasonal inactivated influenza vaccination following current vaccine recommendations in Australia.
Blinding	Experiments were not blinded.

Reporting for specific materials, systems and methods

We require information from authors about some types of materials, experimental systems and methods used in many studies. Here, indicate whether each material, system or method listed is relevant to your study. If you are not sure if a list item applies to your research, read the appropriate section before selecting a response.

Materials & experimental systems

n/a	Involved in the study
☐	☒ Antibodies
☒	☐ Eukaryotic cell lines
☒	☐ Palaeontology and archaeology
☒	☐ Animals and other organisms
☒	☐ Clinical data
☒	☐ Dual use research of concern
☒	☐ Plants

Methods

n/a	Involved in the study
☒	☐ ChIP-seq
☐	☒ Flow cytometry
☒	☐ MRI-based neuroimaging

Antibodies

Antibodies used	We used commercially-available antibodies as per Material and Methods. See Supplementary Table 3-5.
Validation	All antibodies were obtained from commercial vendors. Each antibody used had a validated technical data sheet as per manufacturer's website showing positive staining, and titrated in our laboratory to define the appropriate concentration prior to their use. FACS positive staining is shown in the FACS plots in the main figures and supplementary figures.

Plants

Seed stocks	*Report on the source of all seed stocks or other plant material used. If applicable, state the seed stock centre and catalogue number. If plant specimens were collected from the field, describe the collection location, date and sampling procedures.*
Novel plant genotypes	*Describe the methods by which all novel plant genotypes were produced. This includes those generated by transgenic approaches, gene editing, chemical/radiation-based mutagenesis and hybridization. For transgenic lines, describe the transformation method, the number of independent lines analyzed and the generation upon which experiments were performed. For gene-edited lines, describe the editor used, the endogenous sequence targeted for editing, the targeting guide RNA sequence (if applicable) and how the editor was applied.*
Authentication	*Describe any authentication procedures for each seed stock used or novel genotype generated. Describe any experiments used to assess the effect of a mutation and, where applicable, how potential secondary effects (e.g. second site T-DNA insertions, mosiacism, off-target gene editing) were examined.*

Flow Cytometry

Plots

Confirm that:

☒ The axis labels state the marker and fluorochrome used (e.g. CD4-FITC).

☒ The axis scales are clearly visible. Include numbers along axes only for bottom left plot of group (a 'group' is an analysis of identical markers).

☒ All plots are contour plots with outliers or pseudocolor plots.

☒ A numerical value for number of cells or percentage (with statistics) is provided.

Methodology

Sample preparation	Samples were prepared as described in Methods. Peripheral blood was collected in heparinised or EDTA tubes and serum tubes, with plasma and sera collected after centrifugation, respectively. Peripheral blood monocular cells (PBMCs) were isolated via Ficoll-Paque separation.
Instrument	BD LSRII Fortessa was used for acquisition of data
Software	BD FACS Diva v8.0.1, FlowJo v10
Cell population abundance	No single-cell sorting was performed.
Gating strategy	Gating strategy has been described in the figures, results, figure legends and displayed in Supplementary Figure 2&7.

☒ Tick this box to confirm that a figure exemplifying the gating strategy is provided in the Supplementary Information.
