# Supplementary material for: Atypical B cells and inflammatory profiles delineate immunity to influenza vaccination in First Nations and non-Indigenous people with chronic multimorbidity
Source: Nat Commun. 2026 Jun 5;17:7210. doi: 10.1038/s41467-026-73988-z (PMC13396489; doi:10.1038/s41467-026-73988-z)

nature portfolio

Peer Review File

Atypical B cells and inflammatory profiles delineate immunity to influenza vaccination in First Nations and non-Indigenous people with chronic multimorbidity

Corresponding Author: Professor Katherine Kedzierska

This file contains all reviewer reports in order by version, followed by all author rebuttals in order by version.

Version 0:

Reviewer comments:

Reviewer #1

(Remarks to the Author)
This manuscript from Skinner et al. describes an influenza vaccination study of Australian First Nations and non-Indigenous participants with varying numbers of health co-morbidities. Protection against influenza infection and disease burden are particularly important in individuals with co-morbidities, and First Nations individuals have a high prevalence of these co-morbidities. The authors performed a thorough analysis of influenza vaccine responses across multiple years and multiple time points pre and post vaccination. I applaud their effort to characterize serum cytokines, antibodies, and PBMC phenotypes, including antigen-specific B cells. I do have some concerns about caveats arising from the demographics of the cohort, along with some smaller suggestions described below.

Major points to address:
I notice that the First Nations subset of the cohort is about 10 years older than the non-Indigenous participants. Age is associated with changes in serum cytokine levels, cell differentiation and/or activation, and number of prior influenza exposures. Increasing age is also associated with higher probability of co-morbidities. How is one to separate the numerous age-related effects from co-morbidities and First Nations status in this study? As a starting point, the median ages for participants in the 4 groups in Fig 1h and elsewhere (First Nations/non-Indigenous, co-morbidity yes/no) should be transparently reported so readers can consider the influence of age on the subsequent observations and correlations. I do appreciate the challenges of putting together a cohort of this size and complexity, and that it is not always possible to account for all confounding factors. However, age is a huge confounder for influenza in particular and I have serious concerns that many of the observations could be simply explained by age differences between the groups. Perhaps the authors could perform a correlation analysis between age, co-morbidities, First Nations status, and some of the findings presented near the end of the paper (MBCs, TFH, etc.) to convince us that there is or is not more going on beyond age.
A second area where I have questions is in explaining why baseline titres would be different between First Nations and non-Indigenous populations (Fig 3b). Your own data show that vaccine-induced antibodies are back to baseline levels within a year, so emphasis on vaccination programs in the First Nations population doesn't seem to be a good explanation for the difference. Along the same lines, are there reasonable explanations for why only certain subtypes would be elevated and not others (Fig 4d)? Are recent influenza infection rates sufficient to explain these differences? This is implied in-text (~line 222) and in the discussion but the evidence for this possibility is unclear.

Minor concerns:
Fig 1e/f: Summarizing baseline serum cytokines by summing concentrations from multiple cytokines is an odd choice and I think not appropriate. Cytokine behaviour and effect can vary dramatically at different concentrations depending on the cytokine. No issue with reporting individual cytokine differences as long as these are corrected for false discovery rate, which it appears they were.
Fig 1h/i: T cells that are single positive for PD-1 or CD38 are not necessarily activated and should not be portrayed as such. For example, T cells from younger people are also more likely to be CD38+ at steady state, further reinforcing my concerns about the influence of age on these analyses.
Fig 3: Results section title doesn't seem to agree with conclusions? (line 207)
MBC and TFH section titles (lines 338, 392): Due to the above concerns about covariates with co-morbidities, I would revise these titles to avoid the implication of causation between co-morbidities and MBC/TFH frequencies.

Reviewer #2

(Remarks to the Author)
This study has explored the immunological features of blood samples collected from a powerful cohort of participants from Australian First Nation and non-indigenous communities. In this way, this study can provide unprecedented insight into how co-morbidities among First Nation Australians may perturb the immune response to vaccination. This provides unique, valuable and much-needed insight to an underrepresented area of research.

Below I have listed in detail minor amendments or clarifications that I think would improve the manuscript.

Given that the differential signature in chronic morbidity patients is complex, I would suggest avoiding the overall summary page 2 line 44, which states that the findings “display pro-inflammatory signatures post-IIV” and on page 14 line 453, which states that the vaccines “contributed to baseline inflammation”. Many of the overall changes were suggestive of a reduced response, rather than a pro-inflammatory response per se. Instead, I would suggest changing the wording in these sections to better reflect that the signature is perturbed in a multimodal manner.

Define all abbreviations the first time they are used, e.g. HAI & HA, cTFH1, atBCs

Page 9- Line 294. Suggest re-wording “Non-Indigenous participants overcame their lower baseline” to replace "overcame" with a more descriptive word, which less mechanistic implication.

The representative flow plots for Figure 5B T2 seem to show outliers with a much higher % of + cells compared to the mean data presented in C. Could the authors instead present a sample more representative of the population means?

Figure 5 has gated antigen-specific B cells within the CD19+ IgD population. In the figure legend and main text, these are specified as just MBCs. For accuracy, could these please be referred to as IgD- B cells, as they are in the figure itself? Or specify in the text that this gate is “highly enriched” for MBCs, rather than simply refering to these cells as MBCs.

To explore atypical MBCs, the authors have excluded participants with <5 MBCs (Figure 5F). Could the authors please indicate in the text what proportion of the subset of participants was excluded through this strategy?

Could the authors please clarify in the discussion why they suspect there were no differences in ASCs, given the changes to serum titres in patients with co-morbidities? Assessment of the rHA-specific ASCs would also help clarify this argument, although I appreciate that with the exclusion criteria of participants with <5 antigen-specific cells, a large proportion of the cohort may be excluded.

Reviewer #3

(Remarks to the Author)
Overall Assessment
This manuscript by Skinner et al. comprehensively analyzes the immune response of a cohort of First Nation Indigenous Australians and non-indigenous Australians with various comorbidity statuses over time after influenza vaccination. The study aims to interrogate immunological factors that lead to a predisposition of First Nation people to have more severe and lethal outcomes to respiratory virus infections. The principal finding of this study is that irrespective of ethnicity, vaccine recipients that harbor more comorbidities have an increased basal inflammatory immune landscape highlighted by increased IL-18, pro-inflammatory glycosylation on circulating antibody, and increased natural kill and T cell activation. The authors of this study demonstrate that major immunological differences in response to the vaccine were found not necessarily between ethnic groups (First Nation vs non-indigenous), but tied more to co-morbidities, which were notably more present in the First Nations cohort. Vaccinees with an increased comorbidity burden did not significantly differ in resultant HA+ antibody titers post vaccination but displayed reduced cTfh activation status and appeared to have irregular development of atypical memory B cells. The influenza specific antibody titers waned in a similar timeframe in all groups, suggesting that ethnicity and comorbidity burden did not have substantial impact on the durability of the serological response. However, development of atypical memory B cells in higher comorbidity groups could affect protection against reinfection due to alternative cell fates driven by a more pro-inflammatory environment.
The extensive characterization work in this manuscript is laudable and will provide a potentially useful dataset to inform public health for the demographics analyzed. However, there are several factors and concepts in this manuscript that should be improved and clarified before I can recommend publication. My specific comments are listed below. After sufficient revision, I feel comfortable endorsing this manuscript for publication.
Specific comments
Major Weaknesses:
1) The title of the work feels mismatched to the data presented. The differences in high-comorbidity First Nation individuals vs high-comorbidity non-indigenous individuals were minimal. This suggests that immunologically, these groups are equally ‘high-risk’. Other factors that predispose the First Nation people to be high risk are not sufficiently clarified in the text to make this unilateral claim.
2) Insufficient definition of what a memory B cell is in this paper. What is the flow cytometric definition used in this work?
3) No stated reason as to why IgD- B cells were the focus of analysis compared to bulk CD19+ B cells. Understanding the frequency of Ag specific B cells in these populations and how they compare between groups at T0 and how they change

through the response could be elucidating.
4) What exactly is a ‘Mature IgD- B cell’? Are the authors focusing on class-switched cells, or on some IgM+, IgD- memory/naive cell? This needs to be clarified.
5) While antigen specific B cells are assessed in this study, specific T cells are not. Are the cTfh observed in this study antigen specific?
6) Authors do not reconcile on the fact that despite a lower cTfh activation profile, there is no significant difference in antibody titer between high and low comorbidity groups. The observation is stated, but this apparent discrepancy is not discussed.

Minor Weaknesses:
1) 1) Line 254: ‘This definition, however, does not consider baseline titers’. The meaning of this is unclear, as the definition is set to 4x the baseline titer so by definition considers it. Rephrase to more accurately convey the specific point you want addressed as ‘this definition does not sufficiently characterize the population at baseline’ or to some effect.
2) Line 387: ‘Corroborating an altered differentiation status with distinct gene features of memory B cells’ This paper does not include any gene feature data. You do correctly state at the end of this paragraph that you see differences at the protein level, but in no way did you confirm that the cells you are reporting as atypical memory B cells have genetic profiles similar to those observed in previous publications.
3) Minor typos, such as, in line 662, “recombvinant” is used instead of recombinant.
4) Error in line 324: “First Nations participants had a lower frequency of H3 rHA-specific B cells (median 0.12%) compared to non-Indigenous participants (0.0087%, p=0.0195)”. Median values do not represent data illustrated in Fig 5c or claim in the text.
5) Figure 5B – the tick marks on the flow cytometry plots have been removed. This hides the compression level applied in flowjo, assuming it is bi-ex display. This must always be displayed for transparency.

Version 1:

Reviewer comments:

Reviewer #1

(Remarks to the Author)
The authors have reasonably addressed my questions and made revisions accordingly. No further questions or concerns from me.

Reviewer #2

(Remarks to the Author)
In the revised manuscript and the point-by-point response, the authors have presented a greatly improved version of the manusciprt that addresses all my questions and concerns. In my opinion, the manuscript is now suitable for publication in this journal and will advance knowledge in this understudied and important population.

Reviewer #3

(Remarks to the Author)
The authors have adequately addressed my concerns and I recommend publication.

Open Access This Peer Review File is licensed under a Creative Commons Attribution 4.0 International License, which permits use, sharing, adaptation, distribution and reproduction in any medium or format, as long as you give appropriate credit to the original author(s) and the source, provide a link to the Creative Commons license, and indicate if changes were made.
In cases where reviewers are anonymous, credit should be given to 'Anonymous Referee' and the source.
The images or other third party material in this Peer Review File are included in the article's Creative Commons license, unless indicated otherwise in a credit line to the material. If material is not included in the article's Creative Commons license and your intended use is not permitted by statutory regulation or exceeds the permitted use, you will need to obtain permission directly from the copyright holder.
To view a copy of this license, visit https://creativecommons.org/licenses/by/4.0/

Point-by-point responses to Reviewers' comments

We thank the Reviewers for their comments and insightful suggestions, which have allowed us to further improve the manuscript. We would also like to thank the Editors for the opportunity to resubmit the revised version to *Nature Communications*.

REVIEWER COMMENTS

Reviewer #1 (Remarks to the Author):

This manuscript from Skinner et al. describes an influenza vaccination study of Australian First Nations and non-Indigenous participants with varying numbers of health co-morbidities. Protection against influenza infection and disease burden are particularly important in individuals with co-morbidities, and First Nations individuals have a high prevalence of these co-morbidities. The authors performed a thorough analysis of influenza vaccine responses across multiple years and multiple time points pre and post vaccination. I applaud their effort to characterize serum cytokines, antibodies, and PBMC phenotypes, including antigen-specific B cells. I do have some concerns about caveats arising from the demographics of the cohort, along with some smaller suggestions described below.

We thank the Reviewer for these comments, appreciation of our study, recognising the quality and depth of our analysis as well as the insightful comments to improve our manuscript.

Major points to address:

I notice that the First Nations subset of the cohort is about 10 years older than the non-Indigenous participants. Age is associated with changes in serum cytokine levels, cell differentiation and/or activation, and number of prior influenza exposures. Increasing age is also associated with higher probability of co-morbidities. How is one to separate the numerous age-related effects from co-morbidities and First Nations status in this study? As a starting point, the median ages for participants in the 4 groups in Fig 1h and elsewhere (First Nations/non-Indigenous, co-morbidity yes/no) should be transparently reported so readers can consider the influence of age on the subsequent observations and correlations. I do appreciate the challenges of putting together a cohort of this size and complexity, and that it is not always possible to account for all confounding factors. However, age is a huge confounder for influenza in particular and I have serious concerns that many of the observations could be simply explained by age differences between the groups. Perhaps the authors could perform a correlation analysis between age, co-morbidities, First Nations status, and some of the findings presented near the end of the paper (MBCs, TFH, etc.) to convince us that there is or is not more going on beyond age.

We thank the Reviewer for this suggestion and for their appreciation of our cohort. To address this concern, we have included the following graph depicting ages across the four main subgroups in a revised Figure 1a and have added the following accompanying sentence to Results (Page 4, lines 107-115):

"Comorbidity-affected individuals were older than those without comorbidities for both First Nations (39 years without comorbidities, 54 years with comorbidities, p<0.0001) and non-Indigenous cohorts (40 without comorbidities and 62 years with comorbidities, p<0.0001) (Fig.1a). Notably, median age of First Nations participants with comorbidities trended lower compared to non-Indigenous participants also with comorbidities, however this was not significant (54 and 62 years, respectively) (Fig.1a). This is consistent with epidemiological data which demonstrate Indigenous peoples in Australia and globally are disproportionately impacted by co- and multimorbidity at younger ages and have shorter predicted lifespans compared to non-Indigenous individuals (19, 69, 70, 71)."

<u>New Figure 1a</u>

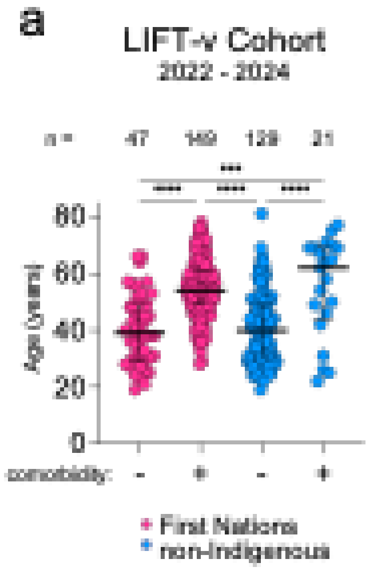

We have also amended our Figures to include age as a parameter for correlation matrices presented in Figure 2e and Figure 7a-b.

New Figure 2e

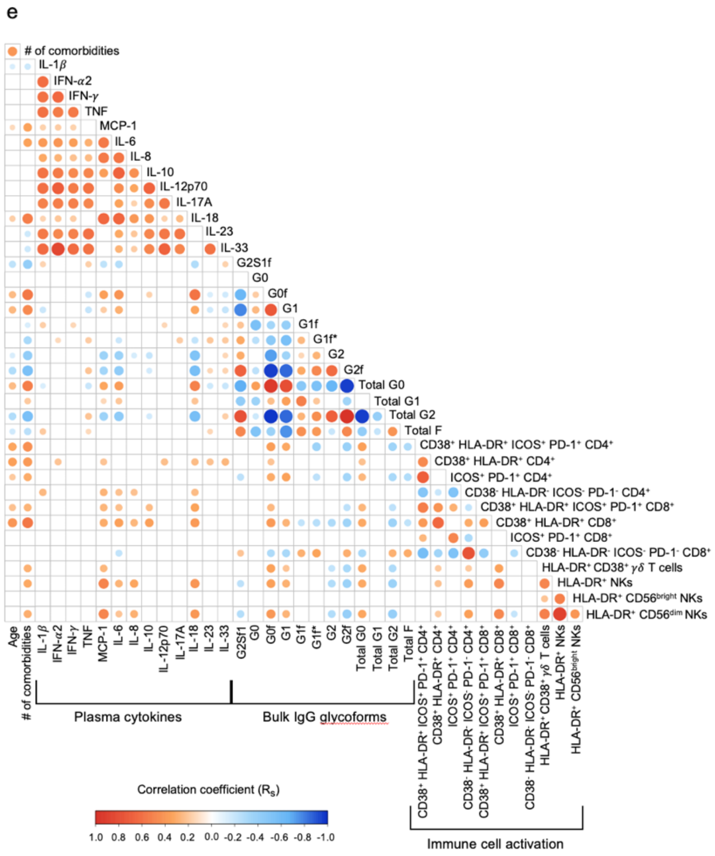

New Figure 7a-b

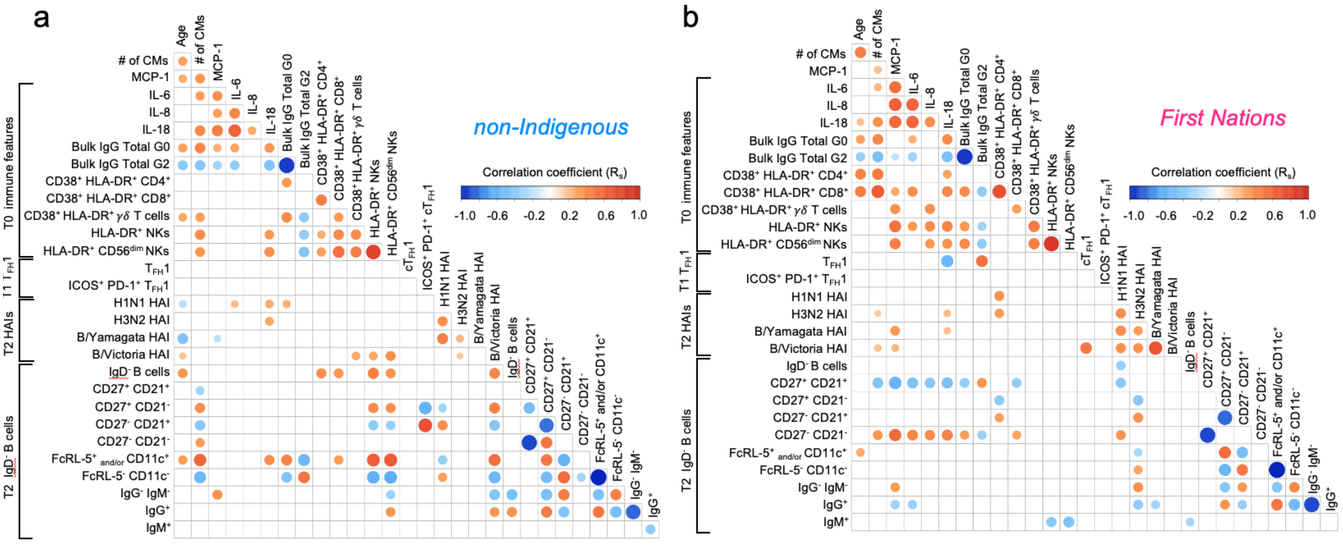

We have included additional discussion of the overlapping nature of ethnicity, comorbidity burden and age as a limitation of this study in the Discussion (Page 18, lines 600-604) (New additions to this passage in **bold**):

“*In our study, we observed disparate comorbidity burden between the groups: 63% of First Nations participants had ≥2 comorbidities, compared with 9% in the non-Indigenous group, clearly revealing disproportionally higher comorbidities in First Nations people.* ***In addition, comorbidity affected participants were significantly older, irrespective of ethnicity. While we demonstrated the presence of comorbidities significantly increased the magnitude of age-associated inflammation and perturbed immunity between subgroups of younger and older adults,*** *these highly overlapping factors* ***(comorbidity burden, ethnicity and age),*** *inherently impacted our statistical analysis as they were not statistically independent variables that could be adjusted to assess their individual contributions to observed immunological differences.”*

In addition, to further confirm that our immunological observations were not strictly driven by age, we have included key features (IL-8, IL-18, IgG G0 glycans and atypical phenotype B cells) graphed according to age subsets for both no-comorbidity and comorbidity groups in Supplementary Figure 10. These data demonsrate that while there are trends of increased inflammation and perturbed immunity with age, the presence of comorbidities significantly increased the magnitude of these features within distinct age groups. These data are also now referenced in the Results section (Page 14, lines 452-456):

“To confirm that our comorbidity-related findings were not driven by ageing, we compared key findings (IL-8, IL-18, IgG G0 and atypical B cells) between paired age groups of comorbidity stratified cohorts. This analysis highlighted that while there are trends of increased inflammation and perturbed immunity with age, the presence of comorbidities significantly increased the magnitude of these features within distinct age groups (Supplementary Fig.10)”.

New Supplementary Figure 10.

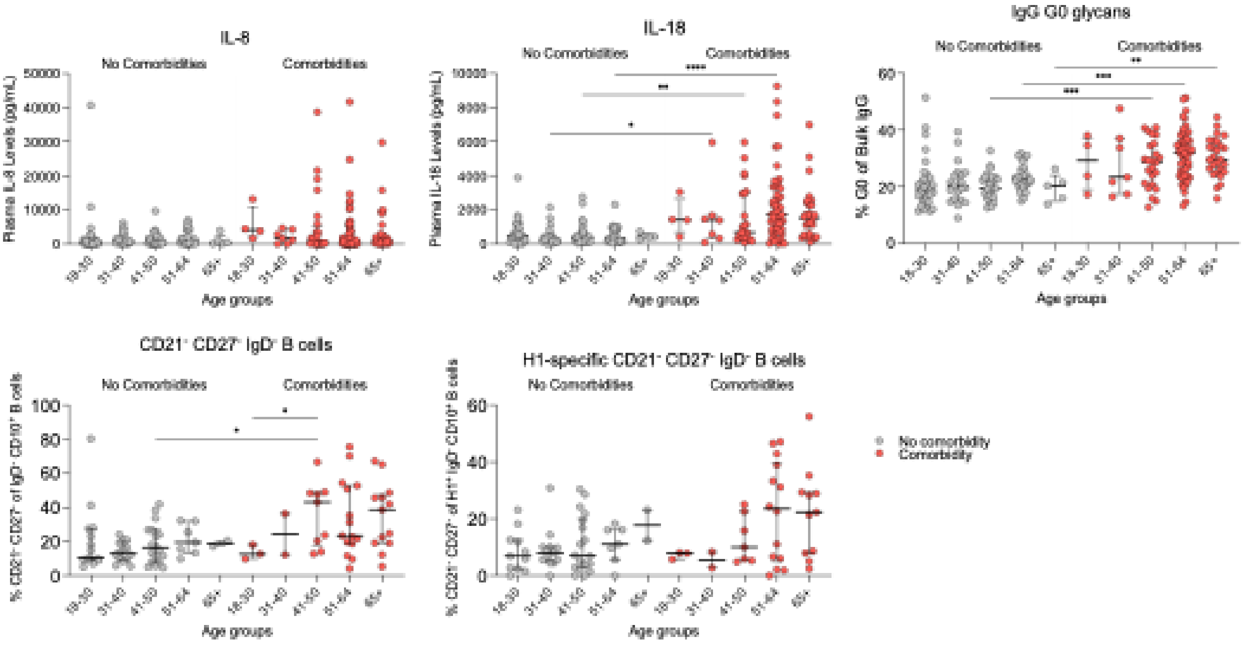

A second area where I have questions is in explaining why baseline titres would be different between First Nations and non-Indigenous populations (Fig 3b). Your own data show that vaccine-induced antibodies are back to baseline levels within a year, so emphasis on vaccination programs in the First Nations population doesn't seem to be a good explanation for the difference. Along the same lines, are there reasonable explanations for why only certain subtypes would be elevated and not others (Fig 4d)? Are recent influenza infection rates sufficient to explain these differences? This is implied in-text (~line 222) and in the discussion but the evidence for this possibility is unclear.

We thank the Reviewer for this insightful question. These data certainly raise many questions as to what drives baseline differences and their physiological relevance.

The Reviewer's comment prompted us to reconsider the physiological relevance of the differences in HAI titres at baseline as we have reported them. The WHO considers a minimum 4-fold change in HAI titre to be a seroconversion event *(Hobson et al. J Hyg (Lond) 1972)*. All significant baseline HAI differences between groups only differed by approximately 2-fold; which by the sensitivity of the assay is not considered to be physiologically different. To limit over interpretation and increase transparency of these data we have toned down the language around how we report these findings, including but not limited to softening the Results section title on Page 7, lines 206-207:

Results section title:
"Similar HAI responses following vaccination in First Nations and non-Indigenous vaccinees despite comorbidities"

Softened language in the Results section (Page 7, lines 217-224; new additions in **bold**):

“Interestingly, we detected higher baseline H1N1-, H3N2- and B/Victoria-specific HAI titres in First Nations participants compared to non-Indigenous individuals (Fig.3b), ***however the geometric mean titres only differed by approximately 2-fold, which by the sensitivity of the assay, may not indicate a physiologically relevant distinction. These higher baseline titres could be due to repeated seasonal vaccinations, which are strongly recommended for Australian First Nations people*** *(9-11), and/or recent infections with related strains,* ***however concordant epidemiological or immunological data is not available to confirm either hypothesis****.”*

Further to the Reviewer’s comment, unfortunately sufficient concordant epidemiological and immunological evidence is not available to make definitive statements on why some subtypes are elevated and not others, as well as the lasting impact of repeated regular vaccinations between our study groups. We have included the following sentence in the Discussion (Page 16, lines 514-516; new additions in **bold**):

“In fact, in our influenza vaccinees with comorbidities, we found high levels of pre-existing influenza-specific immunity, ***potentially*** *due to repeated seasonal vaccinations and/or recent infections with related strains,* ***however concordant epidemiological or immunological data is not available to confirm either hypothesis.****”*

Minor concerns:
Fig 1e/f: Summarizing baseline serum cytokines by summing concentrations from multiple cytokines is an odd choice and I think not appropriate. Cytokine behaviour and effect can vary dramatically at different concentrations depending on the cytokine. No issue with reporting individual cytokine differences as long as these are corrected for false discovery rate, which it appears they were.

We thank the Reviewer for this comment and agree that summing concentrations of multiple cytokines for comparison between groups can be misleading. To remedy this, we removed references to cumulative plasma cytokine concentrations from the Results and instead highlight only specific cytokines individually.

In addition, while we correct for false discovery rate in the analysis of individual cytokines and report them individually, based on the Reviewer’s comment and to improve clarity, we have moved the stacked bar graphs depicting cytokine concentrations to Supplementary Figure 1a. This supplementary figure also includes all measured cytokines graphed individually according to ethnicity, comorbidity and

multimorbidity stratification. Main Figure 1e-f now includes key individual cytokine results that are significantly different between groups (MCP-1, IL-6 and IL-18).

New Main Figure 1e-f

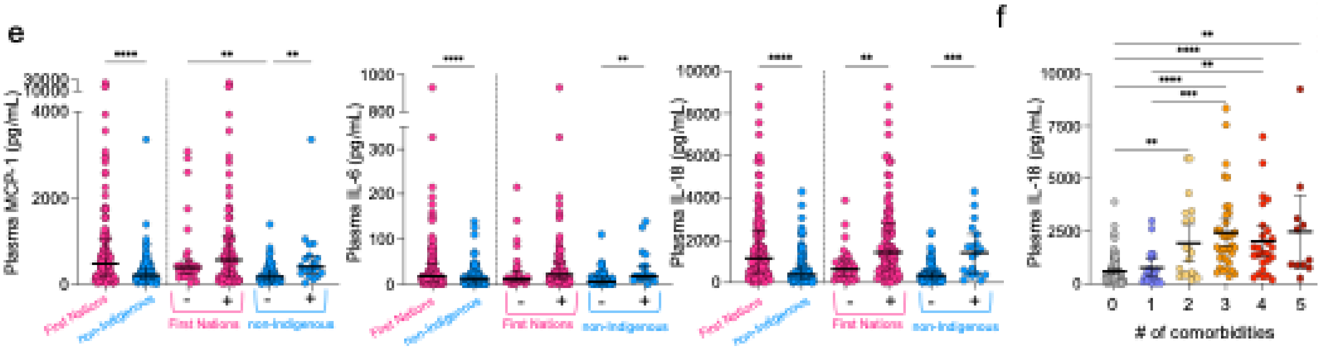

New Supplementary Figure 1a.

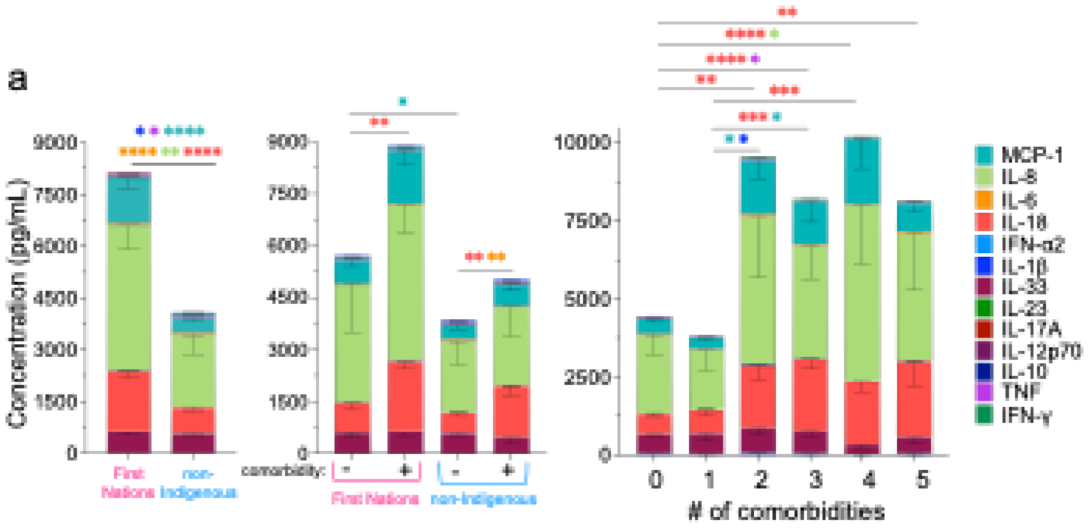

The results text for this section is now as follows (Page 5, lines 124-136; new additions in **bold**):

*"Since we previously linked high baseline inflammation levels in First Nations people with comorbidities with reduced antibody responses following COVID-19 vaccination (17), we first measured plasma inflammatory mediators prior to influenza vaccination in our cohort (**Fig.1e,f, Supplementary Fig.1**). **IL-6, IL-18 and MCP-1** were higher in First Nations people (Fig.1e), while IL-1β and TNF were higher in non-Indigenous participants (**Supplementary Fig.1a, b**). IL-18 was higher in both First Nations and non-Indigenous people with comorbidities (Fig.1e). Plasma concentrations of IL-18 were higher in participants with 2 or more comorbidities compared to those with 1 or no preexisting conditions (Fig.1f). Non-Indigenous participants with comorbidities also had higher concentrations of IL-6 compared to those without comorbidities. First*

Nations people without comorbidities displayed higher concentrations of MCP-1 compared to non-Indigenous participants without comorbidities (Fig.1e).”

Fig 1h/i: T cells that are single positive for PD-1 or CD38 are not necessarily activated and should not be portrayed as such. For example, T cells from younger people are also more likely to be CD38+ at steady state, further reinforcing my concerns about the influence of age on these analyses.

We thank the Reviewer for this comment and agree these single positive populations should not be portrayed as activated. To this end, we removed the stacked bar plots depicting combinations of protein marker expression on $CD4^+$ and $CD8^+$ T cells from Figure 1. We also removed all text referring to these single positive populations, which allows us to highlight the main finding of this analysis – which was that co- and multimorbidity affected individuals have increased proportions of highly activated T cells associated with hyperinflammatory disease states.

This results section on page 5, lines 143-149 is now as follows:

*“We detected a higher proportion of double-positive $CD38^+HLA\text{-}DR^+$ $CD8^+$ T cells, previously associated with influenza disease severity (20, 22, 24, 25), in First Nations with comorbidities compared to First Nations without comorbidities and non-Indigenous participants regardless of comorbidity status (**Fig.1g**). **Higher** frequencies of activated $CD38^+HLA\text{-}DR^+$ $CD4^+$ and $CD8^+$ T cells were found in participants with increasing number of comorbidities, with **the highest** frequencies of these activated T cells detected in participants with 4 or 5 comorbidities compared to participants with no comorbidities (Fig.1h).”*

<u>New Figure 1g-j.</u>

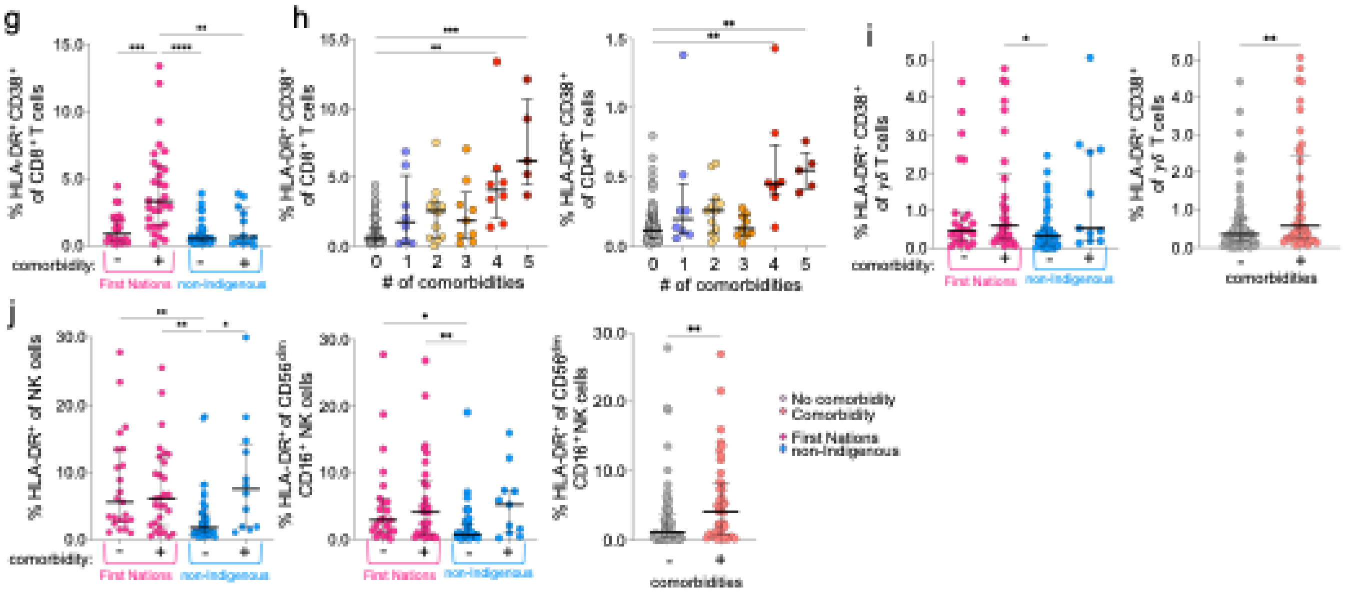

Fig 3: Results section title doesn't seem to agree with conclusions? (line 207) MBC and TFH section titles (lines 338, 392): Due to the above concerns about covariates with co-morbidities, I would revise these titles to avoid the implication of causation between co-morbidities and MBC/TFH frequencies.

We thank the Reviewer for this feedback. We have amended the Results section titles to better represent the data presented.

The Results section title on line 207 (now line 206-207) regarding HAI responses was changed to *"Similar HAI responses following vaccination in First Nations and non-Indigenous vaccinees despite comorbidities"*

The HA-specific B cell Results section title (line 338, now line 340-341) has been changed "to *"Prominent atypical $CD21^{-}$ $CD27^{-}$ phenotype of HA-specific IgD^{-} $CD10^{-}$ B cells in individuals with comorbidities"*

And the T_{FH} cell Results section title (line 392, now line 398) has been changed to *"Reduced activation of $cT_{FH}1$ cells during early IIV response in individuals with comorbidities".*

Reviewer #2 (Remarks to the Author):

This study has explored the immunological features of blood samples collected from a powerful cohort of participants from Australian First Nation and non-indigenous communities. In this way, this study can provide unprecedented insight into how co-morbidities among First Nation Australians may perturb the immune response to

vaccination. This provides unique, valuable and much-needed insight to an underrepresented area of research. Below I have listed in detail minor amendments or clarifications that I think would improve the manuscript.

We thank the Reviewer for their thoughtful comments and appreciation of the importance of our study in this high-risk population.

Given that the differential signature in chronic morbidity patients is complex, I would suggest avoiding the overall summary page 2 line 44, which states that the findings “display pro-inflammatory signatures post-IIV” and on page 14 line 453, which states that the vaccines “contributed to baseline inflammation”. Many of the overall changes were suggestive of a reduced response, rather than a pro-inflammatory response per se. Instead, I would suggest changing the wording in these sections to better reflect that the signature is perturbed in a multimodal manner.

We thank the Reviewer for these suggestions and have changed the manuscript to better reflect our data. On page 2, line 45, we removed the reference to our post-IIV features being pro-inflammatory (new additions in **bold**):

“Our findings thus reveal that vaccinees with multiple chronic diseases, both Australian First Nations and non-Indigenous participants, can mount antibody responses following influenza vaccination, although their cellular immune features, HA-specific IgD$^-$ B cells and cT$_{FH}$1 compartments, display ***features of perturbed humoral axis functionality*** *linked to multimorbidity-associated inflammation and IgG glycosylation patterns at baseline.”*

On page 14, line 466, the passage of text the Reviewer is referring to states that comorbidities contributed to baseline inflammation rather than vaccination itself. We edited this sentence to make this clearer (new additions in **bold**):

“These data further suggest that while First Nations and non-Indigenous vaccinees generated prototypical humoral immune responses to seasonal IIV, ***the occurrence of*** *comorbidities, which were highly prevalent in our First Nations cohort, contributed to baseline inflammation, agalactosylated G0 IgG antibodies, cellular hyperactivation, and atypical lineage CD27$^-$CD21$^-$ memory B cells following vaccination.”*

Define all abbreviations the first time they are used, e.g. HAI & HA, cTFH1, atBCs

We have amended the manuscript to ensure all abbreviations are defined in the introduction of the text or when first used.

Page 9- Line 294. Suggest re-wording “non-indigenous participants overcame their lower baseline” to replace "overcame" with a more descriptive word, which less mechanistic implication.

This sentence (Page 9, line 293-296) has been replaced with:

*"**Despite exhibiting** lower baseline B/Victoria titres, **non-Indigenous participants displayed** a faster B/Victoria-HAI doubling time (40.89 days) compared to First Nations vaccinees (90.65 days, p=0.03), leading to peak titres comparable to First Nations participants (Fig.4c)."*

The representative flow plots for Figure 5B T2 seem to show outliers with a much higher % of + cells compared to the mean data presented in C. Could the authors instead present a sample more representative of the population means?

We replaced the existing flow cytometry plots in Figure 5b with more representative plots of the population medians as displayed in Figure 5c-e.

New Figure 5b

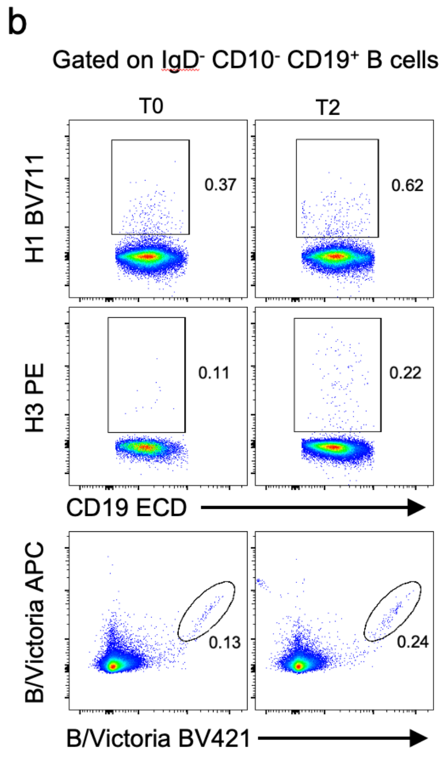

Figure 5 has gated antigen-specific B cells within the CD19+ IgD population. In the figure legend and main text, these are specified as just MBCs. For accuracy, could these please be referred to as IgD- B cells, as they are in the figure itself? Or specify in the text that this gate is "highly enriched" for MBCs, rather than simply referring to these cells as MBCs.

We thank the Reviewer for this feedback. We have changed all references to “MBCs” in the main text and figure legends to “IgD$^-$ CD10$^-$ B cells”. In addition, to improve the accuracy of our reporting, we have included additional details regarding the canonical markers used to identify these cells in the Results (Page 10, line 320-324, new additions in **bold**):

“To define influenza-specific cellular responses, we measured populations of HA-specific IgD$^-$ ***CD10$^-$ CD14$^-$ CD16$^-$ CD3$^-$ CD19$^+$ B cells (enriched for mature B cells that have undergone class-switch recombination, hereby referred to as IgD$^-$ CD10$^-$ B cells)*** *at T0 and T2 post-IIV using recombinant HA (rHA) probes directed against H1, H3 and B/Victoria vaccine components for 2022 and 2023 influenza vaccination seasons (Fig.5a, b; Supplementary Fig.7a; n=98 vaccinees).”*

The flow cytometry gating strategy for HA-specific IgD$^-$ CD10$^-$ B cells is also shown in Supplementary Figure 7A.

To explore atypical MBCs, the authors have excluded participants with <5 MBCs (Figure 5F). Could the authors please indicate in the text what proportion of the subset of participants was excluded through this strategy?

We thank the Reviewer for this suggestion and agree cohort and sample retention following this exclusion strategy should be reported for transparency. We added additional detail to the Results and Figure 5 to more accurately communicate this.

The following sentence was added to the Results (Page 11, lines 342-345, new additions in **bold**):

“To understand the impact of comorbidities on the phenotype of influenza rHA-specific ***IgD$^-$ CD10$^-$ B cells****, we measured expression of activation (CD21) and memory (CD27) phenotypic markers* ***(those with ≥5 rHA$^+$ IgD$^-$ B cells were included in the phenotypic analysis, which permitted 76-90% sample retention across antigen-specificities and timepoints)*** *(Fig.5F; Supplementary Fig.7b).”*

The following sentence with specific inclusion percentages for each probe-specificity and timepoint has been added to the Figure 5 legend (Page 25, lines 843-846):

“Participants that recorded ≥5 rHA probe$^+$ B cells were included this analysis, which allowed inclusion of 76-90% of participants across rHA-probe specificities and sampling timepoints ($H1_{T0}$ = 87/98 (88%), $H1_{T2}$ = 88/98 (90%), $H3_{T0}$ = 76/98 (78%), $H3_{T2}$ = 82/98 (84%), B/Vic_{T0} = 74/98 (76%), B/Vic_{T2} = 82/98 (84%)) to be assessed for phenotype.”

Could the authors please clarify in the discussion why they suspect there were no differences in ASCs, given the changes to serum titres in patients with co-morbidities?

Assessment of the rHA-specific ASCs would also help clarify this argument, although I appreciate that with the exclusion criteria of participants with <5 antigen-specific cells, a large proportion of the cohort may be excluded.

We thank the Reviewer for the opportunity to provide clarity here. We observed no difference in post-vaccination ASC frequency and HAI titres between people with and without comorbidities. Increases in both ASCs and HAI titres following vaccination align with previous publication by our laboratory (*Koutsakos et al. Sci Transl Med 2018, Hensen et al. PNAS 2021*). We clarified this in the Discussion (Page 16, line 517):

"Despite comparable antibody levels ***and ASCs*** *in vaccinees with or without comorbidities, we found that post-IIV, individuals with comorbidities had increased proportions of atypical phenotype influenza-specific memory B cells and suboptimal $cT_{FH}1$ responses, in terms of both frequency and $ICOS^+PD\text{-}1^+$ activation,* ***inferring potential modulation of the humoral axis that could be overlooked due to seroprotective post-IIV HAI titres."***

We agree with the Reviewer that assessment of rHA-specific ASCs would certainly be informative and thank the Reviewer for this suggestion. However, this experiment is not feasible by flow cytometry at the early post vaccination timepoint, as ASCs are highly activated and downregulate expression of surface immunoglobulin/BCR (switching from producing surface Ig to secreted Ig). Thus, rHA-probes do not bind effectively to ASCs as the majority of cells are BCR negative, as previously demonstrated (*Claireaux et al., Sci Adv 2025*).

We addressed the inability to analyse antigen-specific ASCs and cT_{FH} cells (Raised by Reviewer 3, Rebuttal letter Page 16) via flow cytometry as a limitation of our study (Page 19, lines 613-620):

"In addition, our interrogation of early post-vaccination immune responses (ASCs and cT_{FH} cells) was limited by measuring global populations rather than influenza antigen specific. Partially due to the reduced sample size and cell numbers at this optional timepoint, which made a HLA-restricted tetramer-based approach impractical, but also due to the challenges of accurately characterising highly activated rHA-specific antibody secreting B cells via flow cytometry. In the future studies, where sample size and cell numbers permit, measuring antigen-specific cT_{FH} cells and ASCs during early vaccination responses in comorbidity affected individuals would be particularly informative."

<u>Reviewer #3 (Remarks to the Author):</u>

Overall Assessment

This manuscript by Skinner et al. comprehensively analyzes the immune response of a cohort of First Nation Indigenous Australians and non-indigenous Australians with various comorbidity statuses over time after influenza vaccination. The study aims to interrogate immunological factors that lead to a predisposition of First Nation people to have more severe and lethal outcomes to respiratory virus infections. The principal finding of this study is that irrespective of ethnicity, vaccine recipients that harbor more comorbidities have an increased basal inflammatory immune landscape highlighted by increased IL-18, pro-inflammatory glycosylation on circulating antibody, and increased natural kill and T cell activation. The authors of this study demonstrate that major immunological differences in response to the vaccine were found not necessarily between ethnic groups (First Nation vs non-indigenous), but tied more to co-morbidities, which were notably more present in the First Nations cohort. Vaccinees with an increased comorbidity burden did not significantly differ in resultant HA+ antibody titers post vaccination but displayed reduced cTfh activation status and appeared to have irregular development of atypical memory B cells. The influenza specific antibody titers waned in a similar timeframe in all groups, suggesting that ethnicity and comorbidity burden did not have substantial impact on the durability of the serological response. However, development of atypical memory B cells in higher comorbidity groups could affect protection against reinfection due to alternative cell fates driven by a more pro-inflammatory environment.

The extensive characterization work in this manuscript is laudable and will provide a potentially useful dataset to inform public health for the demographics analyzed. However, there are several factors and concepts in this manuscript that should be improved and clarified before I can recommend publication. My specific comments are listed below. After sufficient revision, I feel comfortable endorsing this manuscript for publication.

We thank the Reviewer for these comments and for acknowledging the importance of our study to the public health.

Specific comments
Major Weaknesses:
1) The title of the work feels mismatched to the data presented. The differences in high-comorbidity First Nation individuals vs high-comorbidity non-indigenous individuals were minimal. This suggests that immunologically, these groups are equally ‘high-risk’. Other factors that predispose the First Nation people to be high risk are not sufficiently clarified in the text to make this unilateral claim.

We gratefully thank the Reviewer for this comment and have changed our manuscript title to better represent our findings and cohort. We have now changed the title to:

“Atypical B cells and inflammatory profiles delineate immunity to influenza vaccination in First Nations and non-Indigenous people with chronic multimorbidity”

2) Insufficient definition of what a memory B cell is in this paper. What is the flow cytometric definition used in this work?

We thank the Reviewer for this feedback. To improve the accuracy of our reporting, and address similar concerns raised by Reviewer 2 (Rebuttal letter Page 12) regarding our definition of memory B cells, we included additional detail and rationale of our flow cytometry gating strategy on page 10, lines 320-324 of the Results section (new additions in **bold**):

"To define influenza-specific cellular responses, we measured populations of HA-specific IgD^{-} ***CD10^{-} CD14^{-} CD16^{-} CD3^{-}*** *CD19^{+} B cells* ***(enriched for mature B cells that have undergone class-switch recombination, hereby referred to as IgD^{-} CD10^{-} B cells)*** *at T0 and T2 post-IIV using recombinant HA (rHA) probes directed against H1, H3 and B/Victoria vaccine components for 2022 and 2023 influenza vaccination seasons (Fig.5a, b; Supplementary Fig.7a; n=98 vaccinees)."*

The flow cytometry gating strategy for HA-specific IgD^{-} CD10^{-} B cells is also shown in Supplementary Figure 7A.

3) No stated reason as to why IgD- B cells were the focus of analysis compared to bulk CD19+ B cells. Understanding the frequency of Ag specific B cells in these populations and how they compare between groups at T0 and how they change through the response could be elucidating.

We thank the Reviewer for this question. We chose to focus our analysis to IgD^{-} CD10^{-} B cells as to exclude immature (CD10^{+}) and naïve (IgD^{+}) B cells from our analysis of rHA-specific cells. This is due to a previous study by our laboratory which showed high incidence of non-specific rHA-probe binding by IgD^{+} B cells compared to IgD^{-} populations (*Koutsakos et al. Sci Transl Med 2018*).

4) What exactly is a 'Mature IgD- B cell'? Are the authors focusing on class-switched cells, or on some IgM+, IgD- memory/naive cell? This needs to be clarified.

We thank the Reviewer for seeking this clarification We have now removed all language referring to our Ag-specific B cells as "mature IgD^{-} B cells" and instead call them "IgD^{-} CD10^{-} B cells" as per our flow cytometric definition now in the Results (Page 10, lines 320-322) and flow cytometry gating strategy (Supplementary Fig. 7a). While we specifically gated on IgD^{-} CD10^{-} B cells to enrich for mature and class-switched cells, this strategy allowed us to retain IgM^{+} B cells. IgM^{+} B cells are not class-switched, however they represent a population that upon further activation can produce IgG or IgA and therefore are important part of the memory B cell repertoire in the context of secondary antigen exposures.

5) While antigen specific B cells are assessed in this study, specific T cells are not. Are the cTfh observed in this study antigen specific?

We thank the Reviewer for this consideration. The cT_{FH} cell proportions and activation assessed in this study represent a surrogate for productive early vaccination response in conjunction with increased antibody secreting B cells. Although we assessed these cell populations globally, we believe they likely represent influenza-specific cells. Previous studies from our laboratory and others have shown the majority of cT_{FH} cells produced 7 days post influenza vaccination are influenza antigen-specific and correlated with development of protective antibody titres at later timepoints (*Bentebibel et al. Sci Transl Med 2013, Koutsakos et al. Sci Transl Med 2018, Hensen et al. PNAS 2021*).

In addition, the early timepoint (T1) of this study was optional for participants and therefore limited in the sample and cell numbers available for this analysis. We prioritized examining changes in antigen-specific B cells over a HLA-restricted tetramer-based approach which would be required for antigen-specific T cell analysis. In future studies where sample size and cell numbers permit, measuring antigen-specific cT_{FH} cells during early vaccination responses in comorbidity affected individuals would be informative. We have discussed this along with the topic of antigen-specific ASCs (Raised by Reviewer 2, Rebuttal letter Page 13) in the Limitations section (Page 19, lines 613-620):

"In addition, our interrogation of early post-vaccination immune responses (ASCs and cT_{FH} cells) was limited by measuring global populations rather than influenza antigen specific. Partially due to the reduced sample size and cell numbers at this optional timepoint which made a HLA-restricted tetramer-based approach impractical, but also due to the challenges of accurately characterising highly activated rHA-specific antibody secreting B cells via flowcytometry. Perhaps, in future studies where sample size and cell numbers permit, measuring antigen-specific cT_{FH} cells and ASCs during early vaccination responses in comorbidity affected individuals would be particularly informative."

6) Authors do not reconcile on the fact that despite a lower cTfh activation profile, there is no significant difference in antibody titer between high and low comorbidity groups. The observation is stated, but this apparent discrepancy is not discussed.

We thank the Reviewer for this observation. HAI titres provide quantitative data only, and while they serve as a correlate of protection endorsed by the WHO, they provide limited information about the quality and effectiveness of the humoral responses (i.e. isotype, subclass, neutralisation, Fc function). Therefore, our findings of atypical B cell phenotypes, lower $cT_{FH}1$ activation and an increased failure to seroconvert (4-fold HAI titre increase) highlight a potential modulation of the humoral immune axis that is

obscured by seroprotective post-IIV HAI titres. Future studies into this high-risk cohort to investigate if they have distinct qualitative antibody responses and memory B cell repertoire would be of great interest.

We further commentated on this discrepancy in the Discussion (Pages 15-16, new additions in **bold**):

Page 15, lines 516-524:
"Despite comparable antibody levels ***and ASCs*** *in vaccinees with or without comorbidities, we found that post-IIV, individuals with comorbidities had increased proportions of atypical phenotype influenza-specific memory B cells and suboptimal* $cT_{FH}1$ *responses, in terms of both frequency and* $ICOS^{+}PD\text{-}1^{+}$ *activation,* ***inferring potential modulation of the humoral axis that could be overlooked due to seroprotective post-IIV HAI titres. Indeed, HAI titres are the gold-standard correlate of protection endorsed by the WHO. However, it is a largely quantitative measure that provides limited information about the quality and effectiveness of humoral responses (isotype, subclass, neutralisation, Fc function). Therefore, we believe our findings of atypical memory phenotypes and altered*** $cT_{FH}1$ ***responses warrant further investigation."***

Page 16, lines 533-535:
"Future studies into these high-risk populations to investigate the quality and functionality of antibody responses in addition to the memory B cell repertoire would be of great interest to public health."

Minor Weaknesses:
1) Line 254: 'This definition, however, does not consider baseline titers'. The meaning of this is unclear, as the definition is set to 4x the baseline titer so by definition considers it. Rephrase to more accurately convey the specific point you want addressed as 'this definition does not sufficiently characterize the population at baseline' or to some effect.

We thank the Reviewer for this suggestion and have edited this sentence to more accurately convey our sentiment (Page 8, lines 250-251):

"Historically, responders to IIV have been defined as exhibiting a 4-fold increase in HAI titres post-IIV (44). This definition, however, ***does not sufficiently characterise the physiological relevance of antibody titres at baseline****. We thus defined Low Non-Responders as individuals with baseline HAI titres <40 who did not seroconvert (<4-fold increase)."*

2) Line 387: 'Corroborating an altered differentiation status with distinct gene features of memory B cells' This paper does not include any gene feature data. You do correctly state at the end of this paragraph that you see differences at the protein level, but in

no way did you confirm that the cells you are reporting as atypical memory B cells have genetic profiles similar to those observed in previous publications.

We thank the Reviewer for this comment and agree we have not examined the transcriptional features of these cells. This sentence has therefore been edited to remove the reference to genes (Page 12, lines 391-394):

*"Thus, our data demonstrate, for the first time, increased prevalence of atBCs within influenza HA-specific IgD$^-$ CD10$^-$ CD19$^+$ B cell populations in individuals with comorbidities, further corroborating an altered differentiation status **with distinct features** (45, 46, 56–58, 62, 63) of memory B cells, including HA-specific B cells, in people with chronic diseases."*

3) Minor typos, such as, in line 662, "recombvinant" is used instead of recombinant.

We thank the Reviewer for pointing out this typographical error. We corrected spelling errors and typographical errors in the revised manuscript.

4) Error in line 324: "First Nations participants had a lower frequency of H3 rHA-specific B cells (median 0.12%) compared to non-Indigenous participants (0.0087%, p=0.0195)". Median values do not represent data illustrated in Fig 5c or claim in the text.

We thank the Reviewer for highlighting this inconsistency and have corrected the values in the text (Page 10, line 324-326):

*"First Nations participants had a lower frequency of H3 rHA-specific IgD$^-$ B cells **(median 0.087%)** compared to non-Indigenous participants **(0.12%, p=0.0195)** prior to vaccination (Fig.5c)."*

5) Figure 5B – the tick marks on the flow cytometry plots have been removed. This hides the compression level applied in flowjo, assuming it is bi-ex display. This must always be displayed for transparency.

We thank the Reviewer for highlighting the missing tick marks in Figure 5b. We corrected this and made a new Figure 5b to address Reviewer 1's concern that our flow cytometry plots should more accurately represent the median values shown in Figure 5c-e.

New Figure 5b

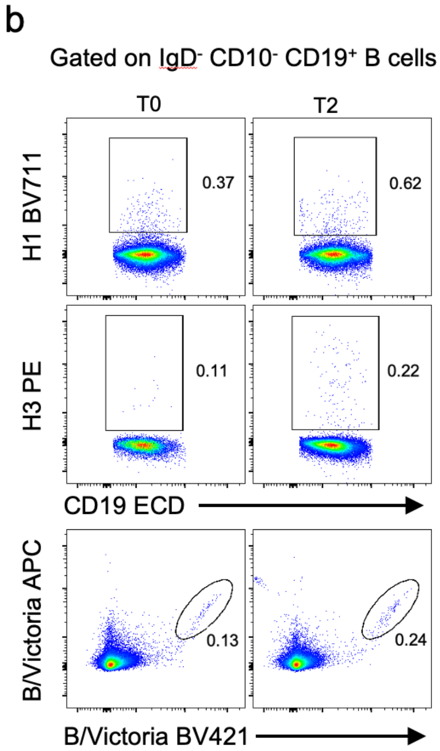

Supplement: Supplementary file 2 — Peer Review file [file 41467_2026_73988_MOESM2_ESM.pdf]
